# Rapid Formation of Vinylene-Linked Covalent Organic Frameworks Promoted by Dipole Moment

**DOI:** 10.1021/acs.chemmater.4c03161

**Published:** 2025-02-18

**Authors:** Clara Ponte, Tania Prieto, Alberto López-Magano, Alicia Moya, Karol Strutyński, Laura Estévez, Soraia P. S. Fernandes, Iñaki Misa, Mariana Sardo, Laura Rodriguez-Lorenzo, Oleg I. Lebedev, Yury V. Kolen’ko, Manuel Melle-Franco, Rubén Mas-Ballesté, Laura M. Salonen

**Affiliations:** † 246702 International Iberian Nanotechnology Laboratory (INL), Avenida Mestre José Veiga, Braga 4715-330, Portugal; ‡ CICECO—Aveiro Institute of Materials, University of Aveiro, Campus Universitário de Santiago, Aveiro 3810-193, Portugal; § CINBIO, Universidade de Vigo, Department of Organic Chemistry, Vigo 36310, Spain; ∥ Department of Inorganic Chemistry (Módulo 7), 16722 University Autónoma of Madrid, Madrid 28049, Spain; ⊥ Universidade de Vigo, Departamento de Química Física, Vigo 36310, Spain; # 69240 Laboratory CRISMAT, UMR 6508, CNRS-ENSICAEN, Caen 14050, France; ∇ Institute for Advanced Research in Chemical Sciences (IAdChem), University Autónoma of Madrid, Madrid 28049, Spain

## Abstract

Vinylene-linked covalent organic frameworks (COFs) are typically prepared via the Knoevenagel reaction under acid- or base-catalyzed solvothermal or benzoic anhydride-catalyzed solid-state conditions under reaction times spanning from 3 to 5 days. Herein, we show that the incorporation of a dipole moment into the COF building block accelerates the formation of ordered material. Under solvent-free Knoevenagel conditions, 3,8-dimethyl-4,7-phenanthroline (Phen) in combination with C_3_-symmetric aldehydes gives access to ordered hexagonal vinylene-linked COFs. A detailed study using triformylbenzene as counterpart evidences the formation of crystalline framework within hours. Theoretical and experimental studies indicate that the fast formation stems from favorable intermolecular interactions of the π-extended Phen building block due to the presence of a dipole moment, which also endows the material with excellent mechanical stability under ball milling. In addition, all prepared materials exhibited broad visible light absorption and narrow band gap energies of ≤2.48 eV and revealed photocatalytic activity for the degradation of dyes.

## Graphical abstract

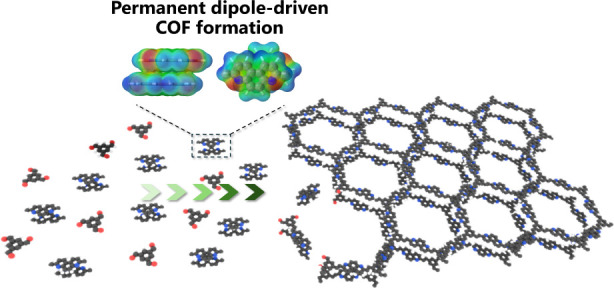

## Introduction

The development of long-range ordered organic 2D materials with a precise periodicity of π-conjugated systems has raised great interest for optical and electronic applications.^1–3^ Moreover, such materials are interesting for photocatalysis due to their extended π-electron delocalization that narrows the conduction and valence energy levels (band gap). Specifically, covalent organic frameworks (COFs) offer a unique opportunity to gain access to ordered π-conjugated materials,^4^ as they allow for atomically precise arrangement of monomers in 2D layers that further extend to a 3D matrix through mainly aromatic interactions, forming ordered crystalline porous high-surface-area materials.^5–12^ In particular, the recently emerged 2D vinylene-linked COFs are gaining considerable attention in the literature due to their attractive characteristics:^13–16^ high chemical stability even under strong alkaline and acidic conditions, notable π-electron delocalization over the 2D lattice, and unique optoelectronic properties, like narrow band gap and rapid charge-carrier mobility.^17–19^ Consequently, pioneering studies underscore the potential of vinylene-linked COFs for several critical applications, including energy storage,^20,21^ electrocatalysis,^22,23^ and metal-free photocatalysis.^24–29^

Early reports of vinylene-linked COFs focused on cyano-functionalized derivatives, which exhibited enhanced photocatalytic activity and stability compared to their imine-linked counterparts, primarily due to their π-conjugated vinylene framework.^30–33^ More recently, great interest has been paid to unsubstituted vinylene-linked COFs synthesized mainly via the Knoevenagel reaction using methyl-bearing active hydrogen compounds, such as trimethyltriazine,^34–39^ pyrazine,^40^ pyridine,^19,41,42^ benzoxazole,^43^ and benzothiazole.^44^

The synthesis of unsubstituted vinylene-linked COFs is typically carried out under a controlled atmosphere using freeze–pump–thaw techniques and solvothermal conditions, which are time-consuming and hinder scalable synthesis. Recently, a solid-state route using benzoic anhydride (Bz_2_O) as a catalyst, first reported by Zhang et al., has proven to be an effective and sustainable solvent-free approach to high-quality vinylene-linked materials,^40^ with typical reaction times of 3–5 days.

Substantial efforts have been dedicated to accelerating COF crystallization by exploring alternative energy sources (e.g., microwave,^45,46^ ultrasound,^47,48^ mechanical,^49–52^ and light^53^) or a careful choice of experimental conditions^54–58^ (e.g., catalyst, solvent) to increase the dynamic error correction during COF formation. Additionally, structural design of monomers has been used to minimize errors and enhance bond reversibility, thus promoting faster crystallization: bifunctional monomers^59^ with amino and formyl groups have been used to ensure ideal stoichiometry and solvent tolerance, yielding highly crystalline imine-linked COFs in 24 h in an array of solvents. Interestingly, amine monomers masked as imines^60^ (e.g., *N*-aryl benzophenone imines) offer superior solubility and stability and enable COF growth via dynamic imine-exchange reactions rather than direct imine condensation, giving access to high-quality imine- and β-ketoenamine-linked COFs within only 5 h. Rapid synthesis of crystalline acylhydrazone COFs^61^ was attributed to favorable interlayer hydrogen bonding and dipole-induced antiparallel stacking, which effectively minimized errors from bond rotation and random stacking, thereby increasing long-range order and significantly reducing reaction times. To date, fast crystallization has been achieved for boronate ester, imine, acylhydrazone, and β-ketoenamine-linked COFs. Recently, the first example of rapid synthesis of vinylene-linked COFs was demonstrated using polyoxometalates as the catalyst, resulting in high-surface-area crystalline COF materials within 12 h.^62^

Given the challenge posed by the low reversibility of the vinylene linkages, our approach focused on accelerating the formation of crystalline COF through favorable intermolecular interactions. We envisioned the target building block to undergo favorable intermolecular interactions prior to and during COF self-assembly, thus compensating for the limited error-correction dynamics of the vinylene linkages and promoting rapid crystallization. Rigid phenanthroline moieties are known for their efficient π-conjugation, strong electron-deficient character, and higher charge-transfer mobility as compared to their bipyridine counterparts with free rotation.^63^ In addition, such large aromatic monomers with permanent dipole moments could promote ordered stacking and undergo efficient intermolecular π–π interactions, allowing for preorganization during COF crystallization.^64–67^

Herein, we report the rapid synthesis of three novel 2D vinylene-linked COFs based on 3,8-dimethyl-4,7-phenanthroline (Phen) via the Knoevenagel reaction. Our studies of COF formation, using 1,3,5-triformylbenzene (TFB) as a counterpart, revealed that the material forms rapidly, with a crystalline framework observed already within 30 min. This was attributed to favorable intermolecular interactions of Phen revealed by nuclear magnetic resonance (NMR) experiments, the synthesis of control COFs, and structure simulations. All Phen-COFs showed light absorption in the visible range with suitable optical band gaps for visible light harvesting applications and exhibited photocatalytic activity in dye degradation.

## Experimental Section

### Synthesis of Phen-COFs

Phen-COFs (TFB-Phen, TFPB-Phen, and TFPT-Phen) were synthesized in 6 mL DURAN culture tubes (borosilicate glass tube, 100 mm × 12 mm) flushed with argon. The aldehyde-based building block (0.31 mmol, 1.0 equiv) was added to Phen (96.8 mg, 0.46 mmol, 1.5 equiv) and benzoic anhydride (157 mg, 0.70 mmol, 2.25 equiv). The mixture was sonicated for 10 min and placed in an oven at 180 °C for the specified reaction time. Thereafter, the monolithic solids were left to cool to room temperature, broken with a hammer, and ground with a mortar and pestle. The solids were soaked in 20 mL of aq 1 M NaOH solution for 20 min and then washed several times with deionized water until pH ≈ 7. Thereafter, to remove unreacted building block molecules, the solids were washed by soaking and decanting DMF (TFB-Phen) or DMA (TFPB-Phen and TFPT-Phen) for 5 times at 80 °C, 2× with water (TFB-Phen) or 5× with THF (TFPB-Phen and TFPT-Phen) at 70 °C, 7× with methanol, 3× with CH_2_Cl_2_, and 3× with hexane. The resulting solids were dried overnight under N_2_ at 90 °C to give TFB-Phen (122.5 mg, 94%) as a green solid, TFPB-Phen (145.0 mg, 72%) as a dark red solid, and TFPT-Phen (158.0 mg, 78%) as a dark orange solid.

### Ab Initio Studies

The Tight Binding (TB) calculations were used to explore the potential energy surface and optimize structures of investigated systems. The xTB Hamiltonian includes D4 dispersion corrections.^68^ All TB calculations were done using the DFTB+ program.^69^ The CREST program^70^ with mixed GFN-FF/GFN2-xTB approach was used to explore possible conformations of the hexagonal analogue. More precise DFT calculations with PBE functional^71,72^ augmented with Many Body Dispersion corrections were applied to compute the COF models and the dipole moments.^73,74^ All DFT calculations were performed using the FHI-AIMS program^75–77^ with “light” or “light_194” numerical orbitals, using the frozen core approximation with a −200 eV cutoff value. As the PBE functional is not suited for band gap calculations, they were calculated using the B3LYP functional.^78–80^

### Photodegradation of Dyes

In the general procedure, in 7 mL vials, 2 mg of ball-milled material, the product of the 72 h synthesis, was added to 2.5 mL of a 10 ppm solution of Methylene Blue (MB) or Para Red (PR) in distilled water or a mixture of CH_3_CN/H_2_O 4:1, respectively. The reactions were sealed through a PTFE-rubber septum, and a needle was used to ensure the air flux. Before the photodegradation tests, adsorption experiments were performed, stirring in dark conditions for 2 h (MB) or 4.5 h (PR). The photodegradation experiments were carried out by stirring under the irradiation of blue light LEDs with λ = 465 nm and *I* = 18 W/m^2^ for the same period of time. Then, the material was collected by filtration, and the supernatants were directly analyzed by UV–vis spectroscopy owing to the intense absorption bands of the dyes at 664 nm (MB) and 483 nm (PR). The photodegradation kinetics were measured after each 20 min in the case of MB dye and 30 min for the PR dye. No degradation was observed when the dye solutions were irradiated in the absence of the photocatalysts. In the mechanistic assays, for the photodegradation experiments under an argon atmosphere, the same general procedure was followed for MB and PR, except that after the reactions were sealed through a PTFE-rubber septum, 3-vacuum freeze–pump–thaw cycles were performed. In the quencher scavenger assays for PR, 5 equiv of the corresponding quencher (DABCO or benzoquinone, dissolved in MeOH) was added. In the desorption experiments of MB, TFB-Phen_BM_ was collected by centrifugation, and 20 mL of distilled water was added. The mixture was sonicated for 4 h at 30 °C. The solution was concentrated to a final volume of 2.5 mL and analyzed by UV–vis spectroscopy. In the recyclability experiments, after the reaction, the material was collected by centrifugation, washed several times with distilled water and MeOH, and dried under vacuum for 1 h before the next catalytic run.

## Results and Discussion

We first tested the reactivity of Phen (Scheme 1) in the Knoevenagel condensation by preparing a small molecule model system through reaction with benzaldehyde, testing both benzoic anhydride (Bz_2_O) and trifluoroacetic acid as catalysts [for details, see Section S2]. After heating for 24 h at 180 °C, both reactions successfully yielded the Phen model system with isolated yields of 63% and 49%, respectively (Figures S1–S3). Due to the higher yield, Bz_2_O was chosen as the catalyst for the COF synthesis.

**Scheme 1:**
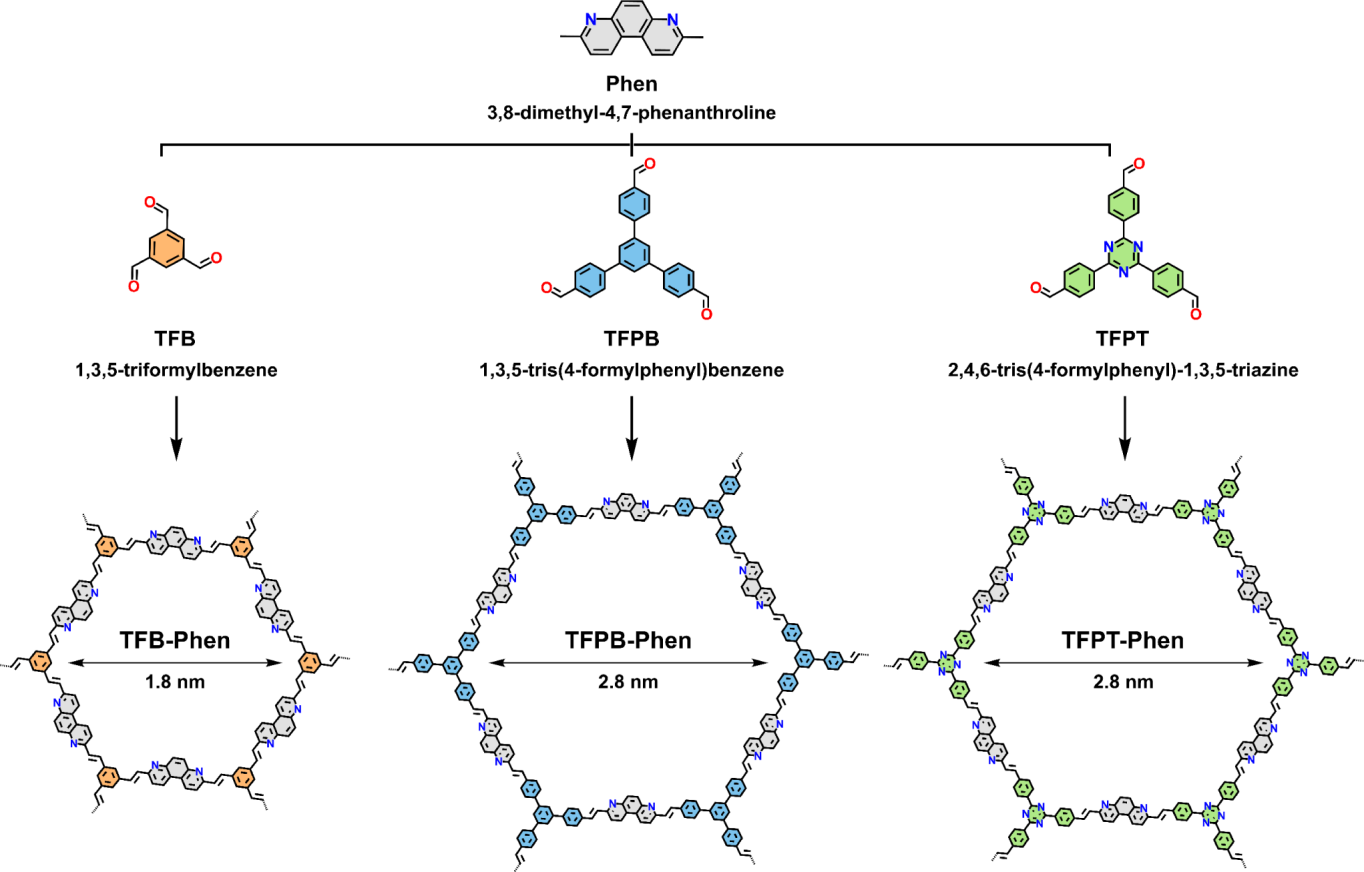
Synthesis of Three 2D Vinylene-Linked Phen-COFs Using Bz_2_O as a Catalyst at 180 °C: TFB-Phen, TFPB-Phen, and TFPT-Phen

To synthesize vinylene-linked COFs, 3-fold-symmetric TFB was reacted with Phen in the presence of Bz_2_O at 180 °C for 72 h. The obtained monolithic solid was broken into smaller fragments, neutralized with aq 1 M NaOH solution, and treated sequentially with *N*,*N*-dimethylformamide, deionized water, methanol, dichloromethane, and hexane to remove unreacted monomers and/or oligomers to afford TFB-Phen in 94% yield as a green solid.

To gain insight into the chemical connectivity of the obtained material, a Fourier-transform infrared spectroscopy (FTIR) analysis was carried out. In the IR spectrum (Figure S4), a significant attenuation was found in the intensity of the characteristic C=O stretching band at 1690 cm^–1^, as well as the appearance of a new band at 1631 cm^–1^, corresponding to the C=C stretching of the vinylene linkages, as also observed in the model system. These findings evidence the successful formation of alkene bonds in the condensation reaction between Phen and TFB. Solid-state ^13^C cross-polarization magic-angle-spinning (CP-MAS) NMR spectrum confirmed the presence of peaks corresponding to the carbon atoms of the Phen moiety at about 145 and 155 ppm (Figure S5).

The collected powder X-ray diffraction (PXRD) pattern (Figure 1a) of the material revealed long-range structural order, featuring four prominent reflections at 2θ = 3.5°, 6.0°, 6.9°, and 9.2°, corresponding to the (100), (110), (200), and (210) diffraction planes, respectively. In addition, a broad Bragg reflection at 2θ = 27.0° was attributed to the graphite-like interlayer interactions at ≈3.3 Å.

**Figure 1:**
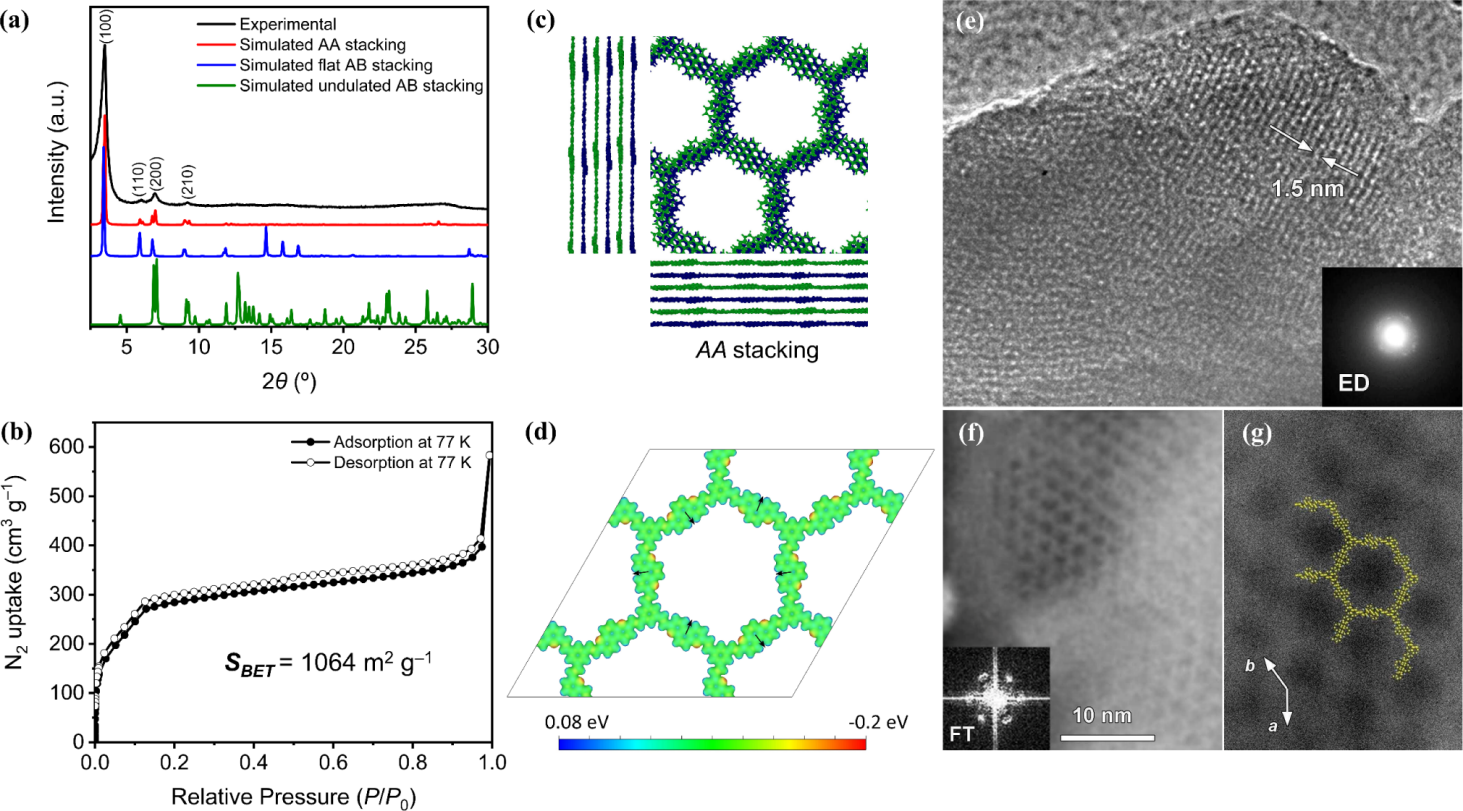
(a) Experimental and simulated *AA*, flat *AB*, and undulated *AB* stacking PXRD patterns of TFB-Phen. (b) Nitrogen physisorption isotherms of TFB-Phen. (c) Side and top views of simulated *AA* stacking. (d) Electrostatic potential in eV of TFB-Phen monolayer projected onto an isodensity surface of 0.02 e^–^/Bohr^3^, arrows highlight the dipole moment on each Phen moiety. (e) Bright-field HRTEM micrograph of TFB-Phen and the corresponding ED pattern. (f) HAADF-STEM image and the corresponding Fourier transform (FT) pattern reflecting the honeycomb structure. (g) Magnified HAADF-STEM image, together with overlaid TFB-Phen structural model.

Computational models were applied to gain a greater understanding of the structure of TFB-Phen. For this, tight-binding models on a simplified molecular analogue were first constructed (see Section S1), and a plethora of potential conformations for single-layer TFB-Phen was obtained. Based on the results of a comprehensive conformational analysis on a hexagonal molecular analogue,^70^ 12 distinct periodic structures for a single-layer material, each representing various conformations, were built and minimized using FHI-AIMS with a PBE/light_194 Hamiltonian.^75–77^ Energy differences ranging from 19 to 172 meV per unit cell were estimated (Table S1 and Figure S6), with the lowest-energy conformation corresponding to a highly ordered hexagonal 2D unit cell with 3 nm length, where all the neighboring Phen moieties are oriented at 120°, with a *C*_3_-symmetry arrangement of TFB-vinylene-Phen (Figure 1d). From this, different layer stackings, *AA* and *AB*, were built and minimized to address the bulk material. This yielded two different layered motifs, planar and undulated (Figures 1c, S7, Tables S2 and S3). Undulated structures have smaller 2D unit cells, larger density, reduced porosity, and a slight thermodynamic advantage (0.1 eV/Phen), yet they showed poor resemblance to the experimental PXRD pattern (Figure 1a), thus fitting previous observations in a similar system.^81^ In contrast, flatter layers, stacked in both *AA* and *AB* arrangements, reproduce the experimental PXRD pattern (Figures S8 and S9), and hence, we deduced that their formation may be favored by kinetic and/or entropic effects.

In addition, different Phen stacking arrangements were explicitly explored, yielding a stabilizing value of ≈0.1 eV/Phen for all antiparallel stackings as compared with the parallel ones (Table S4, Figures S10 and S11). Furthermore, very good agreement between the experimental and simulated antiparallel *AA* stacking PXRD patterns was obtained. Pawley refinement^82^ of the model against the experimental data fitted well to a hexagonal 2D unit cell of 2.9 nm length (Figure S12), confirming the successful preparation of the novel vinylene-linked COF with hexagonal pores.

Next, the pore accessibility of TFB-Phen was analyzed using nitrogen physisorption at 77 K (Figure 1b). A Type I isotherm was found with substantial N_2_ uptake at low relative pressures (*P*/*P*_0_ < 0.05), indicative of microporous character, with a Brunauer–Emmett–Teller (BET) surface area (*S*_BET_) of 1064 m^2^ g^–1^ (Figure S13). The experimental total pore volume (*V*_p_) of 0.58 cm^3^ g^–1^ was determined from the adsorption–desorption isotherms at *P*/*P*_0_ = 0.95. The pore size distribution derived from the quenched-solid density functional theory method based on a slit/cylindrical pore model on carbon revealed a bimodal microporous distribution with maxima at 1.1 and 1.7 nm (Figure S14). Notably, the largest value fits well with a pore size of 1.9 nm obtained from the full hexagonal structure with *AA* stacking (Figure 1c and Table S2). High-resolution bright-field transmission electron microscopy (HRTEM) and high-angle annular dark-field scanning TEM (HAADF-STEM) also confirmed the presence of ordered hexagonal pores of ≈1.5 nm in diameter (Figure 1e–g). The scanning electron micrographs (SEM) unveiled the presence of agglomerated particles with a sponge-like appearance (Figure S15). Thermogravimetric analysis (TGA) revealed TFB-Phen to feature excellent thermal stability up to 350 °C under an inert argon atmosphere (Figure S16).

**Figure 2:**
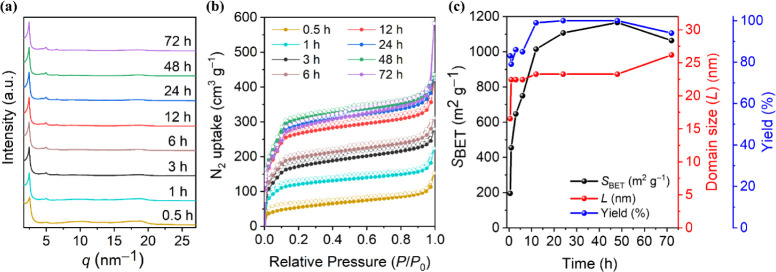
(a) SAXS patterns and (b) nitrogen physisorption isotherms of TFB**-**Phen synthesized at different reaction times. (c) Comparison of *S*_BET_, domain size, and yield for each reaction time.

Motivated by the successful Bz_2_O-catalyzed formation of TFB-Phen, the same reaction conditions were probed using two other 3-fold-symmetric compounds, namely, 1,3,5-tris­(4-formylphenyl)­benzene (TFPB) and 2,4,6-tris­(4-formylphenyl)-1,3,5-triazine (TFPT) (Scheme 1). As for TFB-Phen, the FTIR analysis showed significantly attenuated C=O absorption bands at 1699 cm^–1^, while new vibrational bands appeared at 1630 and 954 cm^–1^ associated with the stretching and bending of the C=C and =C–H bonds for both synthesized materials (Figures S17 and S19). Solid-state ^13^C CP-MAS NMR spectra confirmed the presence of peaks related to the carbon atoms of the Phen moiety at about 145 and 155 ppm, and for TFPT-Phen, an additional peak at 170 ppm was ascribed to the triazine ring (Figures S18, S20, and S21). The PXRD pattern obtained for TFPB-Phen showed Bragg reflections at 2θ = 2.51° and 4.95°, and the one for TFPT-Phen at 2θ = 2.31°, and a broad Bragg reflection centered at 2θ = 25° for both, attributed to the π–π interlayer stacking interactions, indicating the successful formation of layered ordered structures (Figures S22, S23; see also Figures S24, S25 for the simulated PXRD patterns). Both COFs were thermally stable under an inert atmosphere of argon up to 450 °C with total weight losses of 28% for TFPB-Phen (Figure S26) and 40% for TFPT-Phen (Figure S27). SEM images revealed a layered appearance, similar for both materials (Figures S28 and S29). However, despite the observed crystalline order of the materials by PXRD and contrary to what was found previously for TFB-Phen, neither showed discernible porosity during nitrogen physisorption, most likely due to pore collapse upon activation of large-pore COFs.

Intrigued by the high order of crystallinity and surface area of TFB-Phen, we wanted to gain more insight into the formation of the COF material. To understand the evolution of TFB-Phen over time, we carried out the synthesis at different reaction times (0.5, 1, 3, 6, 12, 24, and 48 h), analyzed the structural and textural properties of the synthesized materials, and compared the results to those obtained by the initial 72 h synthesis. Successful formation of the material was confirmed for all reaction times, with the characteristic vibrational band of the vinylene bond at 1631 cm^–1^ evident in all the collected FTIR spectra (Figure S30). Interestingly, all reaction times yielded crystalline products in over 80% yield, indicating a rapid reaction rate (Figure 2a,c blue, respectively). In merely 0.5 h of reaction time, the (100) reflection plane was already visible in the respective small-angle X-ray scattering (SAXS) pattern of TFB-Phen, and after 1 h, all four reflections of TFB-Phen were observed at *q* = 2.48, 4.29, 4.96, and 6.57 nm^–1^. The latter three became more defined after 3 h of reaction, after which no significant changes were observed until 72 h (Figure S31). To quantitatively compare the quality of the obtained COF materials, the domain size (*L*) was determined for each reaction time, as it is directly correlated to the COF crystallinity (for more details, see Section S1). The lowest *L* value of 16.5 nm was observed after 0.5 h of reaction time (Figure 2c red). With a reaction time of 1 h, the *L* value showed a steep increase to 22.4 nm, after which merely minor increases were found with increasing reaction time.

The nitrogen physisorption data of the samples were collected to gain insight into the evolution of the surface area of the material as a function of reaction time (Figure 2b,c black). After 30 min, *S*_BET_ = 196 m^2^ g^–1^ was estimated, correlating with the lower long-range order, as indicated by the lower *L* value. Thereafter, *S*_BET_ increased steadily with increasing reaction time, from 455 m^2^ g^–1^ at 1 h to 647 m^2^ g^–1^ at 3 h, 750 m^2^ g^–1^ at 6 h, and 1015 m^2^ g^–1^ after 12 h, after which minor increases were found, indicating near-complete COF crystallization after 12 h. These results highlight the rapid formation of TFB-Phen; more specifically, no significant changes in the crystallinity of the material were found after 3 h, and after this time, *S*_BET_ was already ≈60% of the highest surface area obtained, and as high as that of several reported vinylene-based COFs catalyzed by Bz_2_O using reaction times ≥72 h.^43,44,83–85^ A similar rapid reaction time was found under polyoxometalate-catalyzed conditions.^62^

Building on our findings, we also tested the synthesis of TFPB-Phen and TFPT-Phen for 12 h, and the PXRD analysis revealed the formation of crystalline materials (Figures S33 and S34). Nitrogen physisorption evidenced *S*_BET_ values of 52 and 47 m^2^ g^–1^ for TFPB-Phen_12 h_ and TFPT-Phen_12 h_, respectively, with bimodal pore size distributions in the microporous and mesoporous ranges (Figures S35–S40).

Next, we strived to gain more insight into the reactivity of the Phen building block by studying small molecule model systems (see Sections S4 and S5). The progress of the reaction of Phen with benzaldehyde as truncated TFB mimic (Scheme S1) in the presence of Bz_2_O at 180 °C was studied at different reaction times (0.5, 1, 3, 6 h), and, after workup, the obtained mixtures were analyzed by ^1^H nuclear magnetic resonance (NMR) spectroscopy (Figure S41) and mass spectrometry (MS). After 0.5 h of reaction time, MS indicated that one of the methyl groups had reacted with benzaldehyde, and after 1 h, the characteristic peaks of the fully formed product were evident in the NMR spectrum, with the corresponding mass detected. The product was completely formed after 3 h in a yield of 75%. Interestingly, the mass 681.3011 was identified after 3 h, which could correspond to an intermediate formed through an attack of a monosubstituted Phen to a disubstituted Phen system (Figure S42), indicating a possible error correction mechanism in Phen-containing COFs through a similar Michael–addition–elimination pathway as previously shown for cyanovinylene COFs.^86^

To understand if the rapid formation of crystalline Phen-COFs stems from dynamic error correction of the vinylene linkages, we next investigated the interconversion of two model systems, namely, (i) the Phen model system formed by reacting Phen with benzaldehyde, and (ii) the 2-methylpyridine (Py) model system prepared by the condensation of Py with benzaldehyde (Scheme S2). First, the Py model system was subjected to the COF formation conditions together with free Phen during 16 h (Scheme S3), and after workup, the resulting mixture was studied by NMR spectroscopy. Interestingly, no traces of the Phen model system were found in the NMR spectrum (Figure S43). Similar results were obtained when the Phen model system was subjected to free Py (Figure S44), with little evidence found of the formation of the Py model system. The addition of 1 equiv of water to the reaction mixture did not alter the results, contrary to what was found for cyanovinylene linkages.^86^

The fact that the reaction intermediate was observed by MS only after 3 h of reaction time and that interconversion was not observed in the NMR spectra in the model system studies after 16 h indicates that the Phen system is not highly dynamic in nature. Hence, it is unlikely that the previously observed rapid crystallization of TFB-Phen stems from the interconversion of the vinylene linkages under Bz_2_O-catalyzed conditions but should instead be ascribed to the favorable intermolecular interactions of Phen. The rigid, π-extended nature of Phen with a computed dipole moment of 3.44 D (Table S5, Section S6), guiding favorable antiparallel arrangement affords rapid preorganization of the compound in a manner that promotes ordered COF formation, and together with small and planar TFB, a crystalline material is thus rapidly formed. Furthermore, in TFB-Phen, the Phen moieties are closer to one another than in the synthesized TFPB-Phen and TFPT-Phen, and in the monolayer of TFB-Phen, the lowest energy conformation is found for a highly ordered monolayer in which all neighboring Phen moieties have dipoles oriented at 120° and *C*_3_ symmetry. In the case of TFPB and TFPT, the increased degrees of freedom of the building blocks and the larger distance between the Phen moieties, together with the propensity for layer slipping and pore collapse upon solvent removal of large-pore COFs,^87,88^ could be fundamental factors for the lower long-range order found for these materials.

To test our hypothesis and elucidate the influence of dipole moment on rapid COF formation, we reacted – under TFB-Phen formation conditions for comparison using 3 h as reaction time – TFB with two isomeric small active hydrogen compounds: 2,5-dimethylpyrazine (PZ)^40^ with nitrogen atoms at *para*-position with no dipole moment and 3,6-dimethylpyridazine (DZ)^89^ in *ortho-*position with a dipole moment of 4.20 D (Table S5). While PZ with no dipole moment gave an amorphous material in 3 h (Figures 3 and S45a), although FTIR confirmed the successful formation of the vinylene linkages (Figure S46), the material obtained from TFB and DZ in 3 h featured a clearly much higher degree of crystalline order (Figures 3 and S45b), supporting the hypothesis that the presence of sizable fixed dipole moments can induce a certain prealignment, promoting rapid crystallization. On the other hand, the material obtained from the reaction of TFB and 6,6′-dimethyl-3,3′-bipyridine (BiPy),^90^ the nonplanar bipyridine counterpart of Phen, showed much less order after 3 h than our TFB-Phen (Figures 3 and S45c). In fact, in addition to the nonplanarity, DFT calculations reveal that, additionally to a Phen-like conformation with a strong dipole of 3.61 D, there is a competing nearly isoenergetic conformation with a smaller perpendicular dipole of 1.31 D (Figures 3 and S45d), which may hinder the stacking kinetically, thus negatively affecting the COF formation.

**Figure 3:**
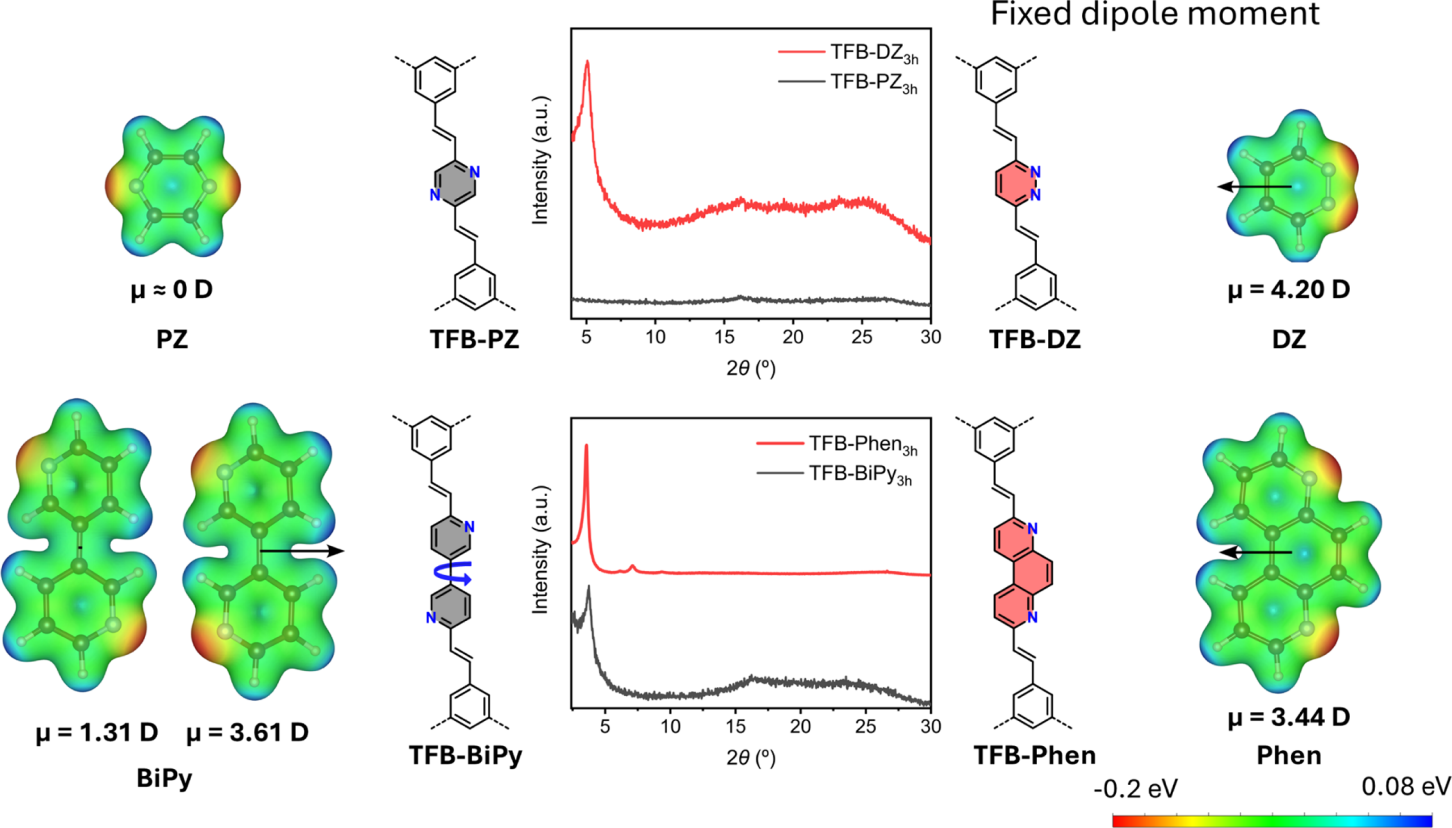
PXRD patterns of the 3 h reaction of TFB with compounds with no permanent dipole moment, PZ and BiPy, and with fixed dipole moment, DZ and Phen. The electrostatic surface potential of nitrogenated molecular analogues are shown with arrows highlighting the dipole moment (1 D = 1 Å) from PBE-MBD/light level DFT simulations.

Next, the optical properties of TFB-Phen, TFPB-Phen, and TFPT-Phen were characterized by ultraviolet–visible diffuse reflectance (UV–vis DR) spectroscopy. All materials showed broad absorption bands covering both UV and visible light regions (Figure S50). The optical band gap energies (*E*_g_) were determined according to the Kubelka–Munk function as 2.48, 1.60, and 1.70 eV for TFB-Phen, TFPB-Phen, and TFPT-Phen, respectively (Figure S51), indicating that the materials are suitable photocatalysts in the visible light region. Furthermore, the computed band gap of TFB-Phen at the DFT B3LYP-light/PBE-MBD-light level gave a value of 2.69 eV (3.18 eV for the single layer), correlating well with the experimental optical band gap of TFB-Phen aligned with the high order of the material (Figure S52 and Table S6).

To allow for dispersion of the Phen-COF materials during photocatalysis, the COF monoliths, the product of solvent-free 72 h synthesis, were subjected to ball milling for 0.5 h at 3000 rpm (in the following, subscript BM will be used to indicate ball-milled samples; for characterization of the COFs after ball milling, see Section S8). UV–vis DR spectra of the ball-milled materials showed broad light absorption bands (Figure 4a), as previously observed for the pristine materials. After ball milling, TFB-Phen_BM_ retained its optical band gap at 2.48 eV (Figure 4b), while for TFPB-Phen_BM_ and TFPT-Phen_BM_, an increase was observed in the *E*_g_ values from 1.60 to 2.02 eV and from 1.70 to 2.16 eV, respectively, most likely due to the disruption of π–π interlayer interactions caused by mechanical exfoliation.^91,92^ Ultraviolet photoelectron spectroscopy (UPS) analysis was performed to determine the energy levels (vs vacuum level) of the valence band (EVB), showing values of ca. −6.47, −6.61, and −6.94 eV for TFB-Phen_BM_, TFPB-Phen_BM_, and TFPT-Phen_BM_, respectively (Figures 4c,d, and S72–S74). Considering the respective *E*_g_ values, the respective conduction bands (ECB) were calculated to be −3.99, −4.59, and −4.78 eV.

**Figure 4:**
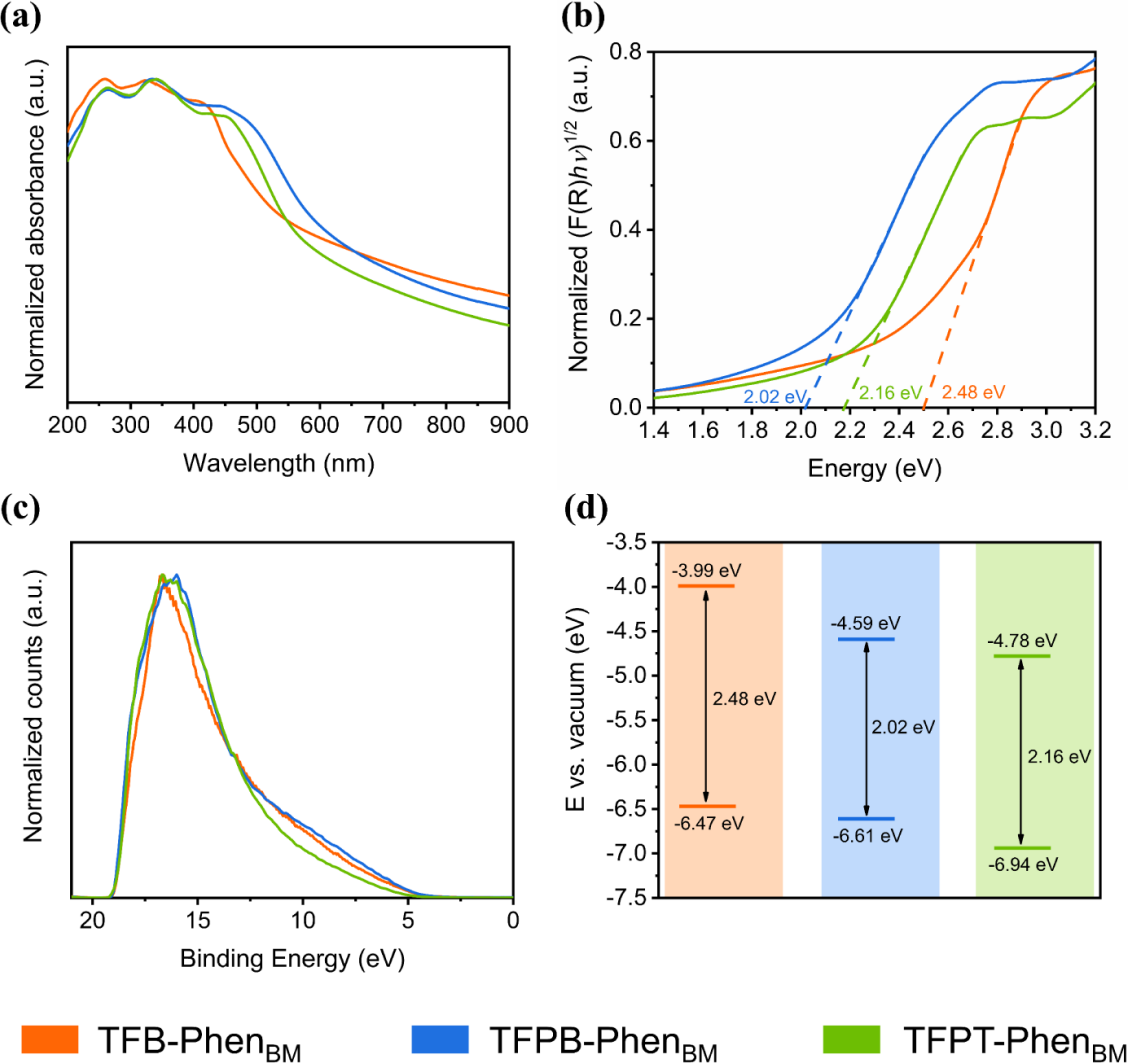
(a) UV–vis DR spectra, (b) optical band gaps estimated from the Kubelka–Munk-transformed UV–vis DR spectra, (c) UPS spectra, and (d) estimated energy band structures of TFB**-**Phen_BM_ (orange), TFPB**-**Phen_BM_ (blue), and TFPT**-**Phen_BM_ (green).

Having in hand novel and photoactive materials, we next investigated their photocatalytic properties. As a model reaction, the performance of the ball-milled Phen-COF materials was studied for the photodegradation of cationic phenothiazine compound Methylene Blue (MB) and neutral azo dye Para Red (PR) (Figure S75) as model organic contaminants (Section S9). Prior to the experiments, the isoelectric point of each material was determined by zeta potential analyses at different pH values (Figure S76), revealing that all materials shared the same isoelectric point of 4.2, which agrees with the acid dissociation constant of Phen.^93^ Adsorption tests ([COF] = 0.8 mg/mL; [dye] = 10 ppm; deionized water for MB, 2 h; CH_3_CN/H_2_O 4:1 for PR, 4.5 h) showed TFB-Phen_BM_ to have a high affinity for MB with 92% adsorbed, whereas TFPB-Phen_BM_ and TFPT-Phen_BM_ demonstrated lower adsorption capacities of 74% and 44%, respectively (Figures S77 and S78).

In contrast to MB, all materials showed limited adsorption efficiencies (<15%) for PR (Figures S77 and S79). Photodegradation experiments carried out under the same conditions with a light source of 465 nm demonstrated complete photodegradation of MB by TFB-Phen_BM_, whereas lower efficiencies of 69% and 19% were observed for TFPB-Phen_BM_ and TFPT-Phen_BM_, respectively (Figures S80 and S81). Desorption tests on both non-irradiated and irradiated samples confirmed that also adsorbed MB was efficiently photodegraded during photocatalytic testing (Figures S82 and S83). To understand if the high surface area of TFB-Phen_BM_ was the source of its superior photodegradation performance, adsorption–desorption and photocatalysis–desorption tests were carried out on TFB-Phen_BM_ synthesized in 1 h (*S*_BET_ = 290 m^2^ g^–1^) and in 6 h (*S*_BET_ = 685 m^2^ g^–1^) (Figures S84–S89), and the results were compared to those of TFB-Phen_BM_ (72 h; *S*_BET_ = 969 m^2^ g^–1^). Surface area was found to have a slight influence on the adsorption efficiency (TFB-Phen_BM1 h_ – 69%, TFB-Phen_BM6 h_ – 94%), but complete photodegradation of MB was achieved with samples regardless of their surface areas (Figures S90–S92), indicating that the surface area plays a secondary role in this case. A comparison of the MB degradation efficiency with literature-reported MOF and COF materials places TFB-Phen among the fast and efficient photodegrading materials (Table S7). On the other hand, 100% photodegradation efficiency of PR was displayed by TFPT-Phen_BM_, while TFPB-Phen_BM_ and TFB-Phen_BM_ showed lower efficiencies of 77% and 65%, respectively (Figures S80 and S93). No photodegradation of MB or PR was observed solely with light irradiation in the absence of the photocatalysts.

Subsequently, tests were carried out to shed light on the kinetics (Figures S94 and S95) and mechanism of photodegradation^94^ by TFB-Phen_BM_ and TFPT-Phen_BM_, which showed the highest degradation efficiency for MB and PR, respectively. Typically, MB is sensitive to various photocatalytic pathways, including direct energy transfer from the photosensitizer to the dye molecule.^95,96^ In contrast, azo dyes like PR typically undergo oxidative degradation, involving the formation of reactive oxygen species, such as singlet oxygen or superoxide radical anion intermediates.^97^ In the absence of oxygen, MB was completely photodegraded by TFB-Phen_BM_ with no dye recovered after desorption (Figures S96 and S97), suggesting that the degradation does not rely on the formation of reactive oxygen species and occurs through direct energy transfer from the excited TFB-Phen_BM_ photocatalyst to the adsorbed MB molecules. Conversely, PR photodegradation over TFPT-Phen_BM_ was inhibited under an anaerobic atmosphere, indicating its dependence on the presence of oxygen and the formation of reactive oxygen species (Figure S98), as expected for a conjugated organic material with triazine moieties.^98^ Benzoquinone (BQ), an O_2_•– scavenger,^99^ and 1,4-diazabicyclo[2.2.2]­octane (DABCO), a singlet oxygen quencher,^100^ both acted as quenchers in PR photooxidation, with BQ exhibiting a more pronounced effect (Figure S99). Hence, the preferred mechanism for PR photooxidation involves the electron transfer pathway leading to the production of superoxide radical anions as the active oxidant. Nonetheless, the possibility of singlet oxygen generation through energy transfer cannot be fully ruled out. The photodegradation activity of TFB-Phen_BM_ for MB was preserved without loss of efficiency for at least four consecutive runs (Figures S100 and S101), and PXRD (Figure S102), FTIR analysis (Figure S103), Raman spectroscopy^101^ (Figure S104), and TEM and SEM analyses (Figures S105 and S106) after photocatalysis confirmed the preservation of the chemical structure, long-range order, and morphology of the TFB-Phen_BM_ material.

## Conclusions

In summary, we report the rapid formation of novel crystalline vinylene-linked Phen-COFs through a solvent-free Knoevenagel condensation. Favorable intermolecular interactions of the Phen building block, as concluded through experiments and calculations, reduce the synthesis time frame of vinylene-linked COFs from a few days to only a few hours, important from both efficiency and sustainability standpoints. The dipole moment of the building block is established to be the driving force for rapid vinylene-linked COF formation: Phen and DZ with strong permanent dipole moments of 3.44 and 4.20 D, respectively, promoted rapid crystallization and ordered COF formation within 3 h, compared to the nonpermanent dipole moment counterparts, BiPy and PZ, that resulted in less crystalline or amorphous products, supporting the hypothesis that dipole-driven prealignment accelerates COF assembly. We believe this strategy to be applicable for the fast synthesis of systems featuring strong dipole moments, and studies exploring the possibility of extending the approach to other intermolecular interactions are currently ongoing in our laboratory. Additionally, the Phen-COFs exhibited broad visible light absorption and narrow band gap energies ≤2.48 eV and showed activity in the photodegradation of model contaminant dyes.

## Supplementary Material



## Notes

Experimental procedures for synthesis of COFs and model systems, respective characterization regarding crystallinity (PXRD), chemical connectivity (FT-IR), additional nitrogen physisorption isotherms, SEM and additional TEM micrographs, optical and electronic properties (UV–vis DR and UPS) (PDF)
