# Supplementary material for: Rapid Formation of Vinylene-Linked Covalent Organic Frameworks Promoted by Dipole Moment
Source: Chem Mater. 2025 Feb 18;37(5):1923–34. doi: 10.1021/acs.chemmater.4c03161 (PMC12134979; doi:10.1021/acs.chemmater.4c03161)
Supplement: Supplementary file 1 [file cm4c03161_si_001.pdf]

## Supporting information

# Rapid Formation of Vinylene-linked Covalent Organic Frameworks Promoted by Dipole Moment

Clara Ponte,[#, ‡] Tania Prieto,[‖] Alberto López-Magano,[/] Alicia Moya,[/] Karol Strutyński,[‡] Laura Estévez,[|] Soraia P. S. Fernandes,[#] Iñaki Misa,[‖] Mariana Sardo,[‡] Laura Rodriguez-Lorenzo,[#] Oleg I. Lebedev,[ʃ] Yury V. Kolen'ko,[#] Manuel Melle-Franco,*[‡] Rubén Mas-Ballesté,*[/,⸆] Laura M. Salonen*[‖,#]

[#]International Iberian Nanotechnology (INL), Avenida Mestre José Veiga, 4715-330 Braga (Portugal)

[‡]CICECO—Aveiro Institute of Materials, University of Aveiro, Campus Universitário de Santiago, 3810-193 Aveiro (Portugal)

[‖]CINBIO, Universidade de Vigo, Department of Organic Chemistry, 36310 Vigo (Spain)

[/]Department of Inorganic Chemistry (Módulo 7), Universidad Autónoma de Madrid, 28049 Madrid (Spain)

[|]Universidade de Vigo, Departamento de Química Física, 36310, Vigo (Spain)

[ʃ]Laboratory CRISMAT, UMR 6508, CNRS-ENSICAEN, 14050 Caen (France)

[⸆]Institute for Advanced Research in Chemical Sciences (IAdChem), Universidad Autónoma de Madrid, 28049 Madrid (Spain)

# Table of Contents


1. General methods ........ 3
2. Synthetic procedures ........ 8
3. Characterization ........ 12
4. $^{1}$H NMR spectroscopy study on the formation of Phen model system ........ 36
5. Calculations on the model system ........ 44
6. Dipole moments ........ 47
7. Optical and electronic properties ........ 49
8. Characterization after ball milling ........ 51
9. Photodegradation of dyes ........ 63
10. References ........ 83

# 1. General methods

## Materials

All chemicals and solvents were purchased from commercial suppliers and used without further purification. The chemical reagents used in the synthesis of the materials were: 1,3,5 triformylbenzene ≥98.0% (TFB) from TCI Chemicals, 1,3,5-tris(4-formylphenyl)benzene ≥95.0% (TFPB) from Carbosynth, 2,4,6-tris(4-formylphenyl)-1,3,5-triazine ≥97.0% (TFPT), 6,6'-dimethyl-3,3'-bipyridine 95% (DMBP) and 3,6-dimethylpyridazine 97% (DZ) from BLD Pharmatech, 3,8-dimethyl-4,7-phenanthroline ≥95.0% (Phen) from Key Organics, benzaldehyde ≥99.0% from Alfa Aesar. 2,5-Dimethylpyrazine 98% (PZ), trifluoroacetic acid ≥99.0% (TFA), *N*,*N*-dimethylformamide ≥99.8% (DMF) and *N*,*N*-dimethylacetamide ≥99.0% (DMA) were purchased from Sigma-Aldrich. Tetrahydrofuran ≥99.0% (THF) and methanol ≥99.0% were obtained from Fisher Scientific. Benzoic anhydride ≥98.0% ($Bz_2O$), *n*-hexane ≥97.0% and dichloromethane extra dry ≥99.8% were bought from Acros Organics.

## Characterization techniques

**Nuclear magnetic resonance** (NMR) spectroscopy $^1H$ and $^{13}C$ NMR analyses were performed on a Bruker AVANCE DPX 400 (400 MHz) spectrometer at Centro de Apoio Científico-Tecnolóxico á Investigación (CACTI) of University of Vigo.

**Magic-angle spinning** (MAS) **solid-state nuclear magnetic resonance** (SS NMR) spectra were acquired on a Bruker Avance III 400 MHz spectrometer operating at a B0 field of 9.4 T, corresponding to a $^{13}C$ Larmor frequency of 100.6 MHz. $^{13}C$ NMR experiments were recorded at a spinning rate of 12 kHz using a triple-resonance 4 mm Bruker MAS probe and the $^{13}C$ chemical shifts are quoted in ppm from glycine (C═O at 176.03 ppm).

The $^1H$–$^{13}C$ cross-polarization (CP) MAS NMR spectra were acquired using a contact time of 2 ms, recycle delay of 5 s, $^1H$ and $^{13}C$ radio frequency (RF) field strength of 79 (50–100% RAMP-CP shape) and 61 kHz, respectively. During acquisition, a SPINAL-64 $^1H$ decoupling with a pulse length of 5.5 at a RF field strength of 83 kHz was employed.

**Mass spectrometry** (MS) analyses were carried out on a VG Autospec M. spectrometer.

**Small-angle X-ray scattering** (SAXS) analyses were performed on a SAXSess mc$^2$ instrument from Anton Paar operated at 40 kV and 50 mA. The samples were placed in a holder with Mylar windows for the measurements at 25 ºC and the data were collected with an image plate detector. All data are background corrected. The domain size ($L$) of the material was determined according to the following equation[1]:

$$L = \frac{\pi}{w}$$

where $w$ is the half-width at half-maximum of the (100) reflection ($HWHM_{100}$), calculated by adjusting the Lorentzian function to the baseline-corrected reflections using Origin Pro 2018 software. Materials with higher crystallinity present high $L$ values. Small variations in the mass of the analyzed samples could originate minor differences in the domain size.[2]

**Powder X-ray diffraction** (PXRD) measurements were performed on a PANalytical X'Pert PRO MRD diffractometer operating with a Cu radiation sources ($\lambda$ = 1.5405980 Å) at 45 kV and 40 mA. Intensity data were collected in transmission mode by a step-counting method (step 0.03º) in continuous mode in the $0.5 \leq 2\theta \leq 30$º range.

**Ultraviolet-visible diffuse reflectance spectroscopy** (UV-vis DRS) measurements were performed on a UV-VIS JASCO V-780 using the integer sphere JASCO ISN-901i in reflectance mode. The UV-Vis reflectance spectra were collected in 900–190 nm range (step 0.5 nm, scan speed 200 nm/min, bandwidth 2 nm).

**Fourier-transform infrared spectroscopy** (FTIR) analyses were performed on a VERTEX 80v FT-IR spectrometer (Bruker) in attenuated total reflectance (ATR) mode. The FTIR spectra were collected in 400–4000 cm$^{-1}$ range using a resolution of 4 cm$^{-1}$ and 64 scans. The FTIR data is background corrected and reported in frequency of absorption (cm$^{-1}$).

**Thermogravimetric analysis** (TGA) measurements were performed using a TGA/DSC 1 STARe system (Mettler Toledo). The powdered CQDs were heated from 30 to 900 °C at 3 °C/min under a continuous argon flow.

**Nitrogen physisorption** isotherms were recorded with Quantachrome Autosorb and Autosorb iQ instruments at 77 K. The samples were degassed for 12 h at 90 °C under high vacuum prior to the measurements. Surface areas of the obtained powders were estimated by the multipoint Brunauer–Emmett–Teller (BET) method using ASIQwin™ and BETSI[3] softwares. Pore size distributions were calculated using quenched solid density functional theory (QSDFT) method ($N_2$ 77 K on carbon).

**Ultraviolet photoelectron spectroscopy** (UPS) analyses were carried out on a ESCALAB 250Xi, Thermo Scientific equipment. The energy level of the valence bands ($E_{VB}$) was determined by subtracting the UPS width from the excitation energy (He I, 21.22 eV). The energy level of the conduction bands ($E_{CB}$) was calculated by following the equation:

$$E_{CB} = E_{VB} + E_g$$

where $E_{VB}$ is the energy level of the valence band and $E_g$is de optical band gap energy.

**Scanning electron microscope** (SEM) micrographs were acquired on FEI Quanta 650 FEG under high vacuum through an Everhart-Thornley detector (ETD), with an acceleration voltage of 3.00 kV at a working distance of 10 mm and a beam spot size of 3.0.

**Transmission electron microscopy** (TEM) micrographs including electron diffraction (ED), bright field high-resolution TEM (HRTEM) and high angle annular dark field scanning TEM (HAADF–STEM) experiments was performed using an aberration image and probe-corrected cold FEG JEOL ARM200F microscope operated at 80 and 200kV, equipped with a large collection angle CENTURIO EDX detector, Orius Gatan CCD camera and GIF Quantum spectrometer. TEM samples were prepared by mechanically grinding the material in an agate mortar together with ethanol and depositing the obtained suspension on Cu holey carbon grid.

**Raman** spectra were acquired using am Alpha 300 Access confocal Raman microscope (Witec, Germany) involving a 785 nm excitation laser line, a 20× objective, a high-resolution grating (300 gr/mm) and CCD camera for detection. The acquisition conditions for the Raman spectra were 4 s, 60 accumulations and 5 mW of laser power. The spectra were first processed with WITec Project software (i.e., average of 5 single spectra). Baseline correction and cosmic ray removal were performed in Grams AI (Version 9.3; ThermoFisher Scientific) and plotting in Origin 9.0.0 (OriginLab, USA).

Previous to the **photocatalytic** experiments, the materials were placed in a Tube drive (ST-20/50) and milled with stainless steel balls of 5 mm diameter for 30 min at 3000 rpm using an IKA Ultra-Turrax Tube Drive P control to homogenize the particle size of the materials.

After the photocatalytic test, **UV-visible absorption spectroscopy** of the (filtered) reaction media was performed in an Agilent 8453 spectrometer using quartz cuvettes of 1 cm width to determine the photocatalytic efficiency of the materials towards dye degradation.

The zeta potential of each COF at different pHs were measured by **electrophoretic scattering light** (ELS) carried out on a Nano Zetasizer ZS equipment from Malvern Instruments using DTS1070 cells at 25 ºC.

# Ab-initio Studies

## TFB-Phen COF conformational search

The TFB-Phen COF Monolayer (ML) has several possible conformers. For each arm, the orientation of Phen and two vinylene moieties connected from both sides could differ. The CREST-assisted conformational search for simple hexagon found >500 conformers. This ensemble was too large for reasonable comparison with DFT and adaptation to periodic conditions. Therefore, several monolayer structures with different Phen and vinylene group orientations were prepared by hand and analyzed using DFT. See Table S1 and Figure S6.

For selected ML structures the bulk conformers were tested, in various AA and AB stacking arrangements. Overall, around 30 conformations were investigated using PBE-MBD/light_194 Hamiltonian with 3x3x2 k-point grid. Selected systems were reevaluated at PBE-MBD/light at 3x3x4 k-point grid, including full geometry optimization (see Table S2, Table S3, and Figure S7). Note that AA structures show clearly separated layers. Contrarily, in the case of AB, in the interlayer arrangement the sheets are not clearly separated, the linkers bend and go into the pores created by the layer above/below. This also causes the AB structure to be roughly twice the density of the AA ones. The best AA structures are consistently produced by combining layers where Phen units in the layers are in antiparallel local arrangement. Similar strategy was employed in study of TFPB-Phen and TFPT-Phen. Figures S24 and S25 show the obtained simulated PXRD patterns.

## 2. Synthetic procedures

### Synthesis of model system 3,8-di((*E*)-styryl)-4,7-phenanthroline (1)

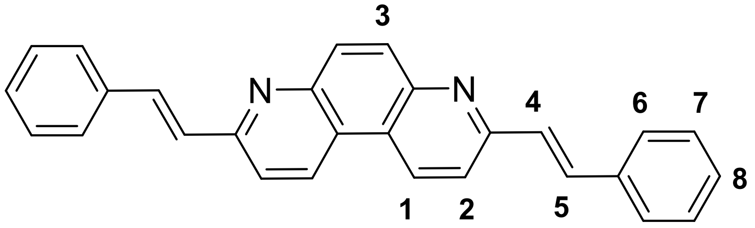


In a 6 mL DURAN® culture tube, **Phen** (100.0 mg, 0.48 mmol, 1.0 equiv.) was reacted with benzaldehyde (196 μL, 1.92 mmol, 4.0 equiv.) and benzoic anhydride (163 mg, 0.72 mmol, 1.5 equiv.) or trifluoroacetic acid (551 μL, 0.72 mmol, 1.5 equiv.) as catalyst under $N_2$ atmosphere. The reaction was sonicated before being placed in a pre-heated oven at 180 ºC for 24 h. Then, the reaction mixture was left to cool down to room temperature, neutralized with aqueous saturated $NaHCO_3$ solution and mixed with $CH_2Cl_2$. A liquid–liquid extraction was performed 3 times, and the aqueous phase was extracted with $CH_2Cl_2$. The organic phase was washed with brine, dried over $Na_2SO_4$, and evaporated to dryness. Purification by column chromatography ($SiO_2$; $CH_2Cl_2$→$CH_2Cl_2$/EtOAc 4:1) gave model system **1** as light brown solid ($Bz_2O$: 113 mg, 63%; TFA: 91 mg, 49%).

**$^1$H NMR (400 MHz, $(CD_3)_2SO$):** 9.26 (d, *J* = 8 Hz, 2H, H-C(1)), 8.17 (s, 2H, H-C(3)), 8.06 (d, *J* = 8 Hz, 2H, H-C(2)), 7.93 (d, *J* = 16 Hz, 2H, H-C(5)), 7.78 (d, *J* = 8 Hz, 4H, H-C(7)), 7.57 (d, *J* = 16 Hz, 2H, H-C(4)), 7.46 (t, *J* = 8 Hz, 4H, H-C(6)), 7.38 (t, *J* = 8 Hz, 2H, H-C(8)).

**$^{13}$C NMR (100 MHz, $(CD_3)_2SO$):** 155.6, 146.8, 136.3, 134.1,132.2, 131.9, 128.9, 128.8, 128.2, 127.3, 123.5, 120.8.

**FT-IR:** 3081, 3057, 3027, 2966, 1631, 1586, 1529, 1492, 1469, 1447, 1413, 1357, 1332, 1292, 1272, 1204, 1184, 1146, 1088, 1073, 1025, 958, 850, 813, 756, 729, 681, 604, 567, 550, 487.

**HR-ESI-MS:** ***m/z* (%):** 385.170082 (100, $[M+H]^+$, calculated for $C_{28}H_{21}N_2^+$: 385.169925).

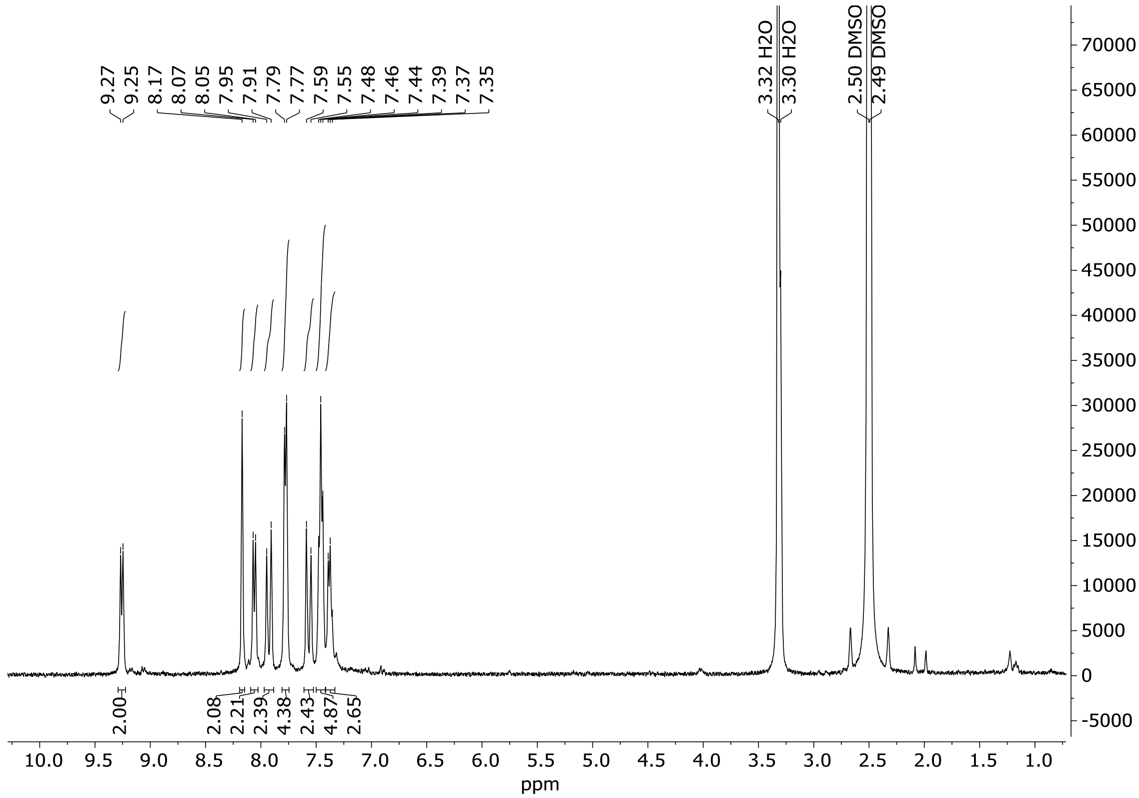


**Figure S1.** $^{1}$H NMR spectrum (400 MHz, $(CD_3)_2SO$) of model system **1**.

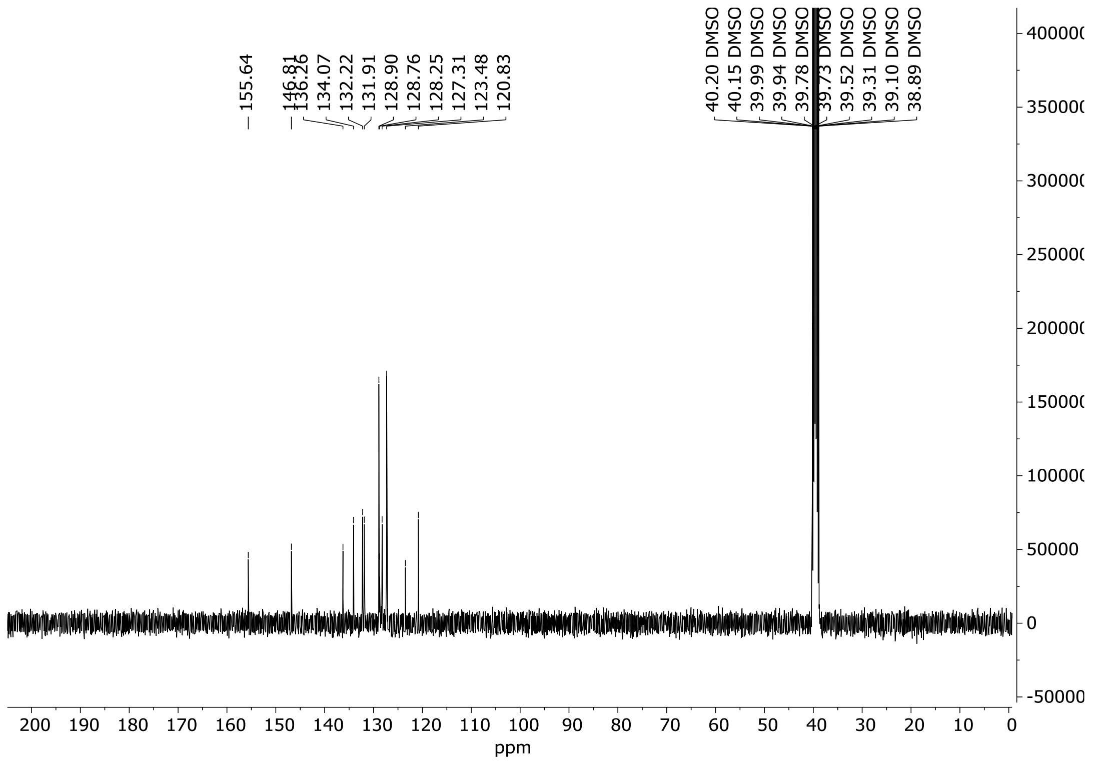


**Figure S2.** $^{13}$C NMR spectrum (100 MHz, $(CD_3)_2SO$) of model system **1**.

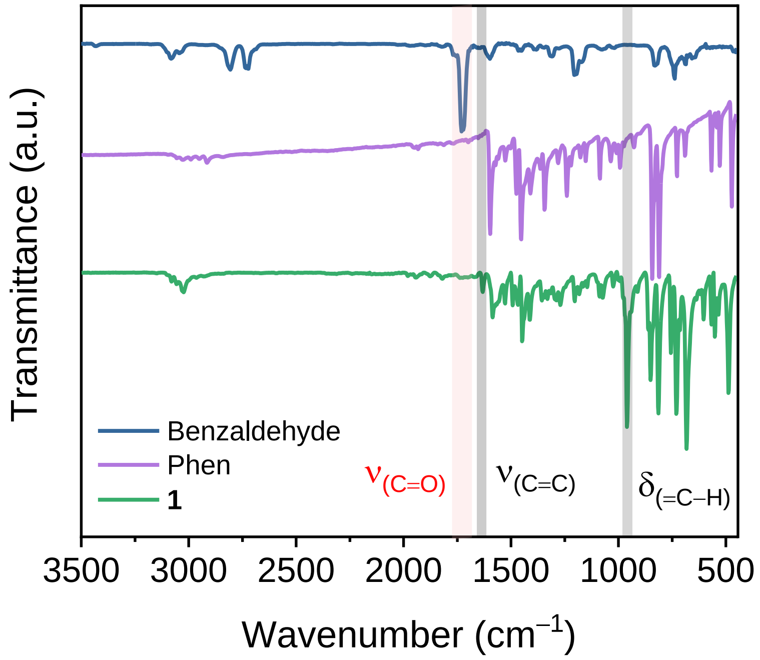


**Figure S3.** FT-IR spectra of model system **1** and the starting materials.

## Synthesis of TFB-PZ, TFB-DZ and TFB-BiPy COFs

Phen-COFs (TFB-PZ, TFB-DZ and TFB-BiPy) were synthesized in 10 mL DURAN® culture tubes (borosilicate glass tube, 98 mm x 16 mm) flushed with argon. TFB (50.3 mg, 0.31 mmol, 1.0 equiv.) was added to **PZ, DZ or BiPy** (0.46 mmol, 1.5 equiv.) and benzoic anhydride (157 mg, 0.70 mmol, 2.25 equiv.). The mixture was sonicated for 10 min and placed in the oven at 180 ºC for 3 h or 72 h. Thereafter, the monolithic solids were left to cool down to room temperature, broken with a hammer, and ground with a mortar and pestle. The solids were soaked in 20 mL of aq. 1 M NaOH solution for 1 h, and then washed with deionized water until pH ≈ 7. Thereafter, to remove unreacted building block molecules, the solids were washed by soaking and decanting 5x with THF, 3x with $CH_2Cl_2$, and 3x with hexane. The resulting solids were dried overnight under $N_2$ at 60 °C.

## 3. Characterization

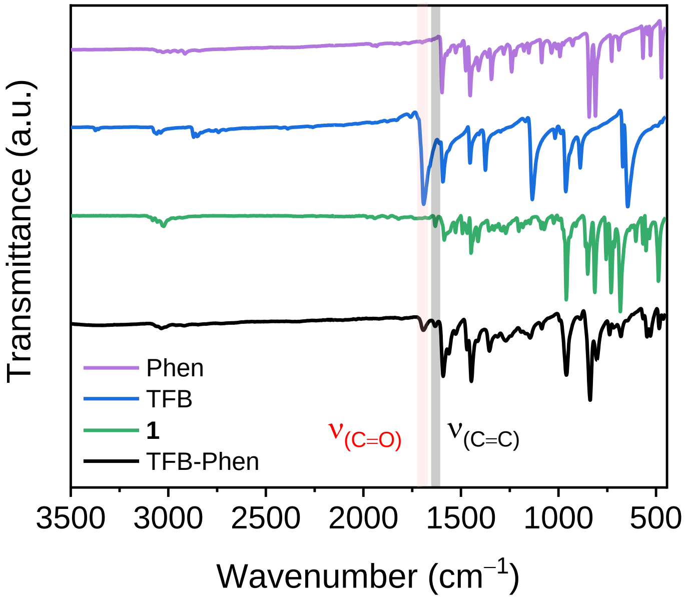


**Figure S4.** FT-IR spectra of TFB-Phen, model system **1**, and the starting materials.

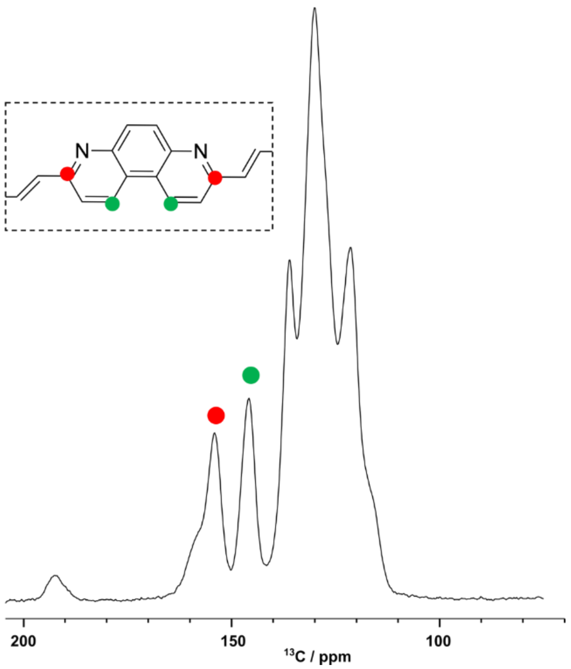


**Figure S5.** $^{13}C$ CP-MAS spectrum of TFB-Phen, acquired at a MAS spinning rate of 12 kHz on a 9.4 T spectrometer. Simplified fragment of the Phen moitey is shown. The peaks between 110–140 ppm are attributed to the remaining aromatic carbons present in TFB-Phen, and the resonance at 190 ppm is ascribed to unreacted aldehyde moieties of TFB.

**Table S1.** DFT Energies and cell parameters of various TFB-Phen COF monolayer conformers. Optimized using PBE/light_194 Hamiltonian.

| Minimum # | Energy (eV) | dE (meV) | a (Å) | b (Å) | Gamma (º) |
|---|---|---|---|---|---|
| 1 | -71686.64669 | 0 | 30.020 | 30.080 | 119.94 |
| 2 | -71686.62764 | 19.05 | 31.490 | 29.960 | 124.68 |
| 3 | -71686.52514 | 121.55 | 30.110 | 30.180 | 119.81 |
| 4 | -71686.52161 | 125.08 | 30.230 | 30.220 | 120.35 |
| 5 | -71686.50639 | 140.3 | 30.220 | 31.000 | 122.76 |
| 6 | -71686.50526 | 141.43 | 30.040 | 31.660 | 124.83 |
| 7 | -71686.4932 | 153.49 | 30.910 | 29.440 | 120.08 |
| 8 | -71686.49259 | 154.1 | 30.250 | 28.560 | 115.32 |
| 9 | -71686.47613 | 170.56 | 30.220 | 30.210 | 119.97 |
| 10 | -71686.47544 | 171.25 | 30.270 | 30.280 | 120.36 |
| 11 | -71686.47481 | 171.88 | 30.350 | 30.280 | 120.61 |
| 12 | -71686.47435 | 172.34 | 30.170 | 30.140 | 119.59 |

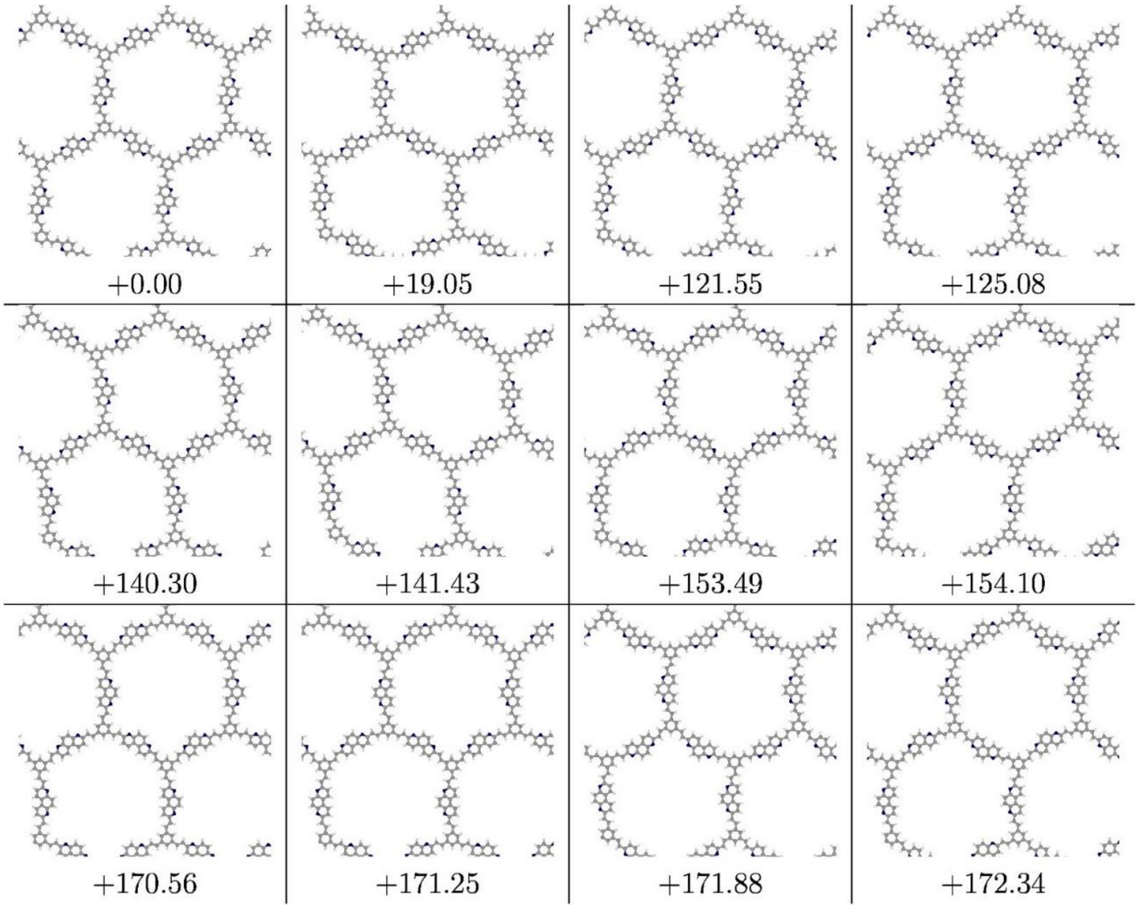


**Figure S6**. Various TFB-Phen COF monolayer conformers with their relative energies (meV). Optimized using the DFT PBE/light_194 Hamiltonian.

**Table S2**. Two-layer bulk conformers of TFB-Phen. Optimized at PBE/light using 3x3x4 k-point for bulk and 3x3x1 k-point for monolayers. Bold font indicates reasonable reproduction of experimental PXRD pattern.

| Conformation | dE (meV) | BE (eV) | $BE_{Phen}$ (eV) | Density (g/cm³) | Pore limiting diameter (Å) | Surface area (m²/g) | Pore accessible volume (cm³/g) |
|---|---|---|---|---|---|---|---|
| AB undulated | 0 | -6.02 | -1.00 | 1.257 | 3.43 | 215 | 0.072 |
| **AA anti** | **703** | **-5.31** | **-0.89** | **0.529** | **19.13** | **2143** | **1.183** |
| **AB flat** | **3851** | **-2.17** | **-0.36** | **0.575** | **8.65** | **2203** | **0.970** |
| Monolayer | nan | 0.00 | 0.00 | 0.018 | NA | NA | NA |

**Table S3**. Unit cells of obtained conformations of TFB-Phen COF. Bold font indicates reasonable reproduction of experimental PXRD pattern.

| Conformation | Density (g/cm³) | a (Å) | b (Å) | c (Å) | Alpha (º) | Beta (º) | Gamma (º) |
|---|---|---|---|---|---|---|---|
| AB undulated | 1.257 | 29.61 | 28.83 | 8.55 | 83 | 45 | 123 |
| **AA anti** | **0.529** | **30.26** | **30.1** | **6.98** | **95** | **101** | **120** |
| **AB flat** | **0.575** | **30.22** | **29.86** | **6.23** | **90** | **90** | **120** |
| Monolayer | 0.018 | 30.04 | 30.07 | 99.78 | 90 | 90 | 120 |

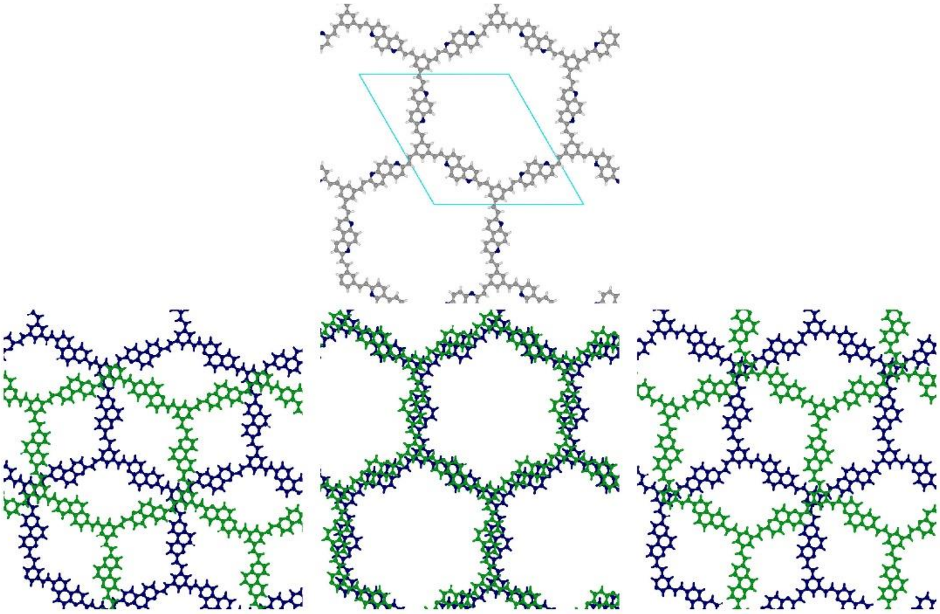


**Figure S7.** Example conformations of TFB-Phen.

## Simulated PXRDs

Most AA conformations reproduce the PXRD pattern reasonably well and only two investigated AB structures show simulated PXRD patterns similar to the experimental one. Figure S8 shows examples of PXRD pattern reproduction by theoretical structures; Figure S63 shows more detailed information. Both figures used a zero-point correction of 0.0772° obtained from Pawley refinement of the experimental PXRD.

Prominent reflections in the experimental pattern are $2\theta$ = 3.5º, 6.0º, 6.9º, and 9.2º. The different computed conformations of two layered structures show variations in the simulated PXRD pattern. From the Pawley refinement (Figure S12), the 3.5° corresponds to the (100) diffraction plane, 6.0° corresponds to (110) reflection, 6.9° attributed to the (200) reflection, and the peak near 9.2° corresponds to the (210) reflection plane.

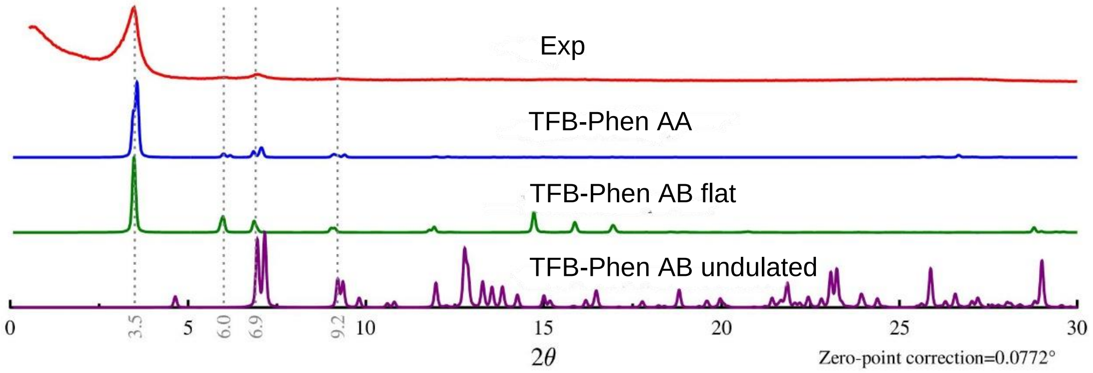


**Figure S8.** Simulated PXRD pattern of AA and AB flat conformations of TFB-Phen.

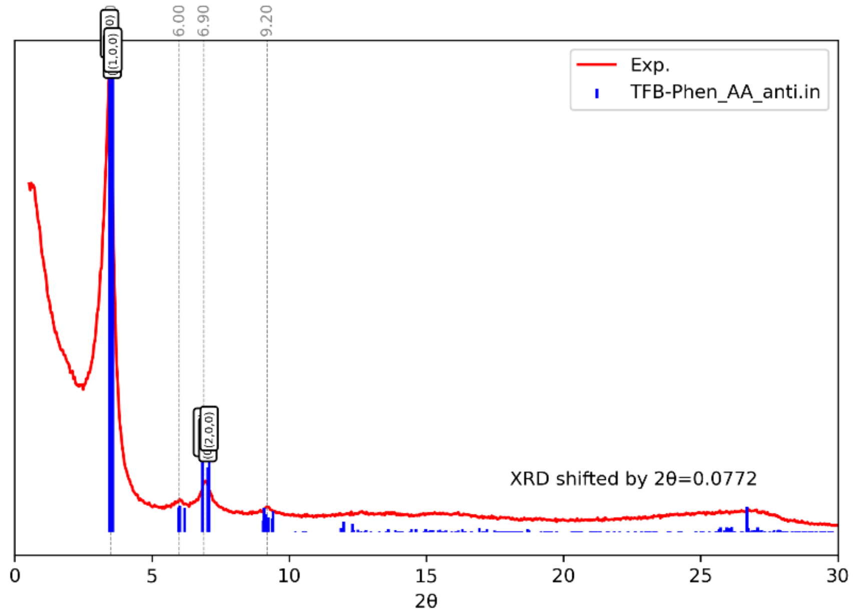


**Figure S9.** Simulated PXRD pattern of AA conformation of TFB-Phen.

## Phen Stacking

To study the structure and energetics of stacking of two Phen moieties, with and without periodic boundary conditions, were investigated with PBE-MBD/light_194 Hamiltonian. Figures S10 and S11 along with Table S4 show different optimized structures with their corresponding binding energies (BE).

**Table S4**. Energies and geometries of various stacking arrangements of Phen molecular units. All structures were optimized at PBE-MBD/light_194 level. The (1D) periodic columns of two Phen units are labelled "columnar", while two non-covalently interacting Phen are denoted as "dimer".

| Type | Conformation | Energy (eV) | dE (meV) | BE per Phen (eV) | c (Å) | Distance between planes (Å) | Distance between center of molecules (Å) |
|---|---|---|---|---|---|---|---|
| Columnar | AA anti | -31083.92513 | 0 | -0.51 | 7.73 | 3.279 | 4.534 |
| Columnar | AB anti | -31083.91215 | 12.98 | -0.50 | 7.70 | 3.268 | 4.444 |
| Columnar | AB anti | -31083.80107 | 124.06 | -0.45 | 7.90 | 3.13 | 5.204 |
| Columnar | AA | -31083.7235 | 201.63 | -0.41 | 8.00 | 3.351 | 3.791 |
| Columnar | Exp. | -31083.722 | 203.13 | -0.41 | 7.99 | 3.354 | 3.782 |
| Columnar | Exp. flat | -31083.70232 | 222.81 | -0.40 | 7.84 | 3.208 | 4.522 |
| Columnar | AB | -31083.69956 | 225.57 | -0.40 | 7.83 | 3.364 | 4.207 |
| Dimer | AA anti | -31083.3888 | 536.33 | -0.48 | 99.27 | 3.405 | 3.906 |
| Dimer | AB anti | -31083.38458 | 540.55 | -0.48 | 99.08 | 3.419 | 3.891 |
| Dimer | AB | -31083.28788 | 637.25 | -0.38 | 99.27 | 3.461 | 3.849 |
| Dimer | AA | -31083.18378 | 741.35 | -0.28 | 99.32 | 3.777 | 3.778 |
| Phen | molecular | -15541.45244 | NA | NA | 99.56 | NA | NA |

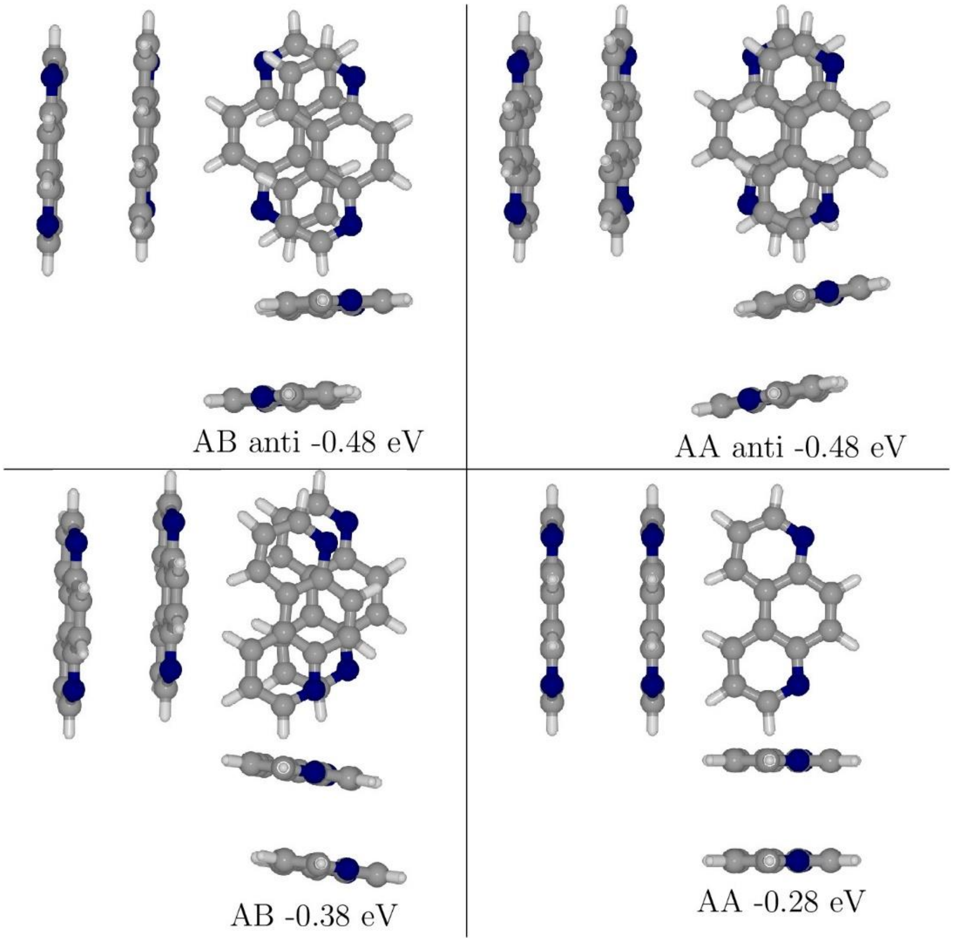


**Figure S10.** Structure of Phen dimer and binding energy per Phen in eV.

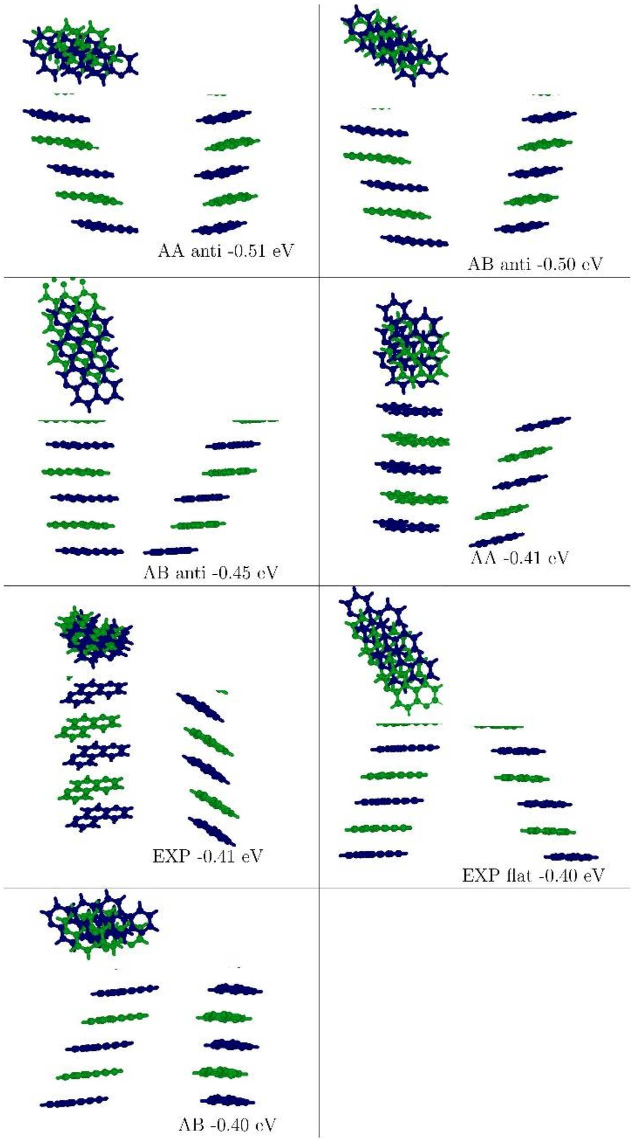


**Figure S11.** Structure of columnar stacking of Phen molecular stacks with 1D periodic boundary conditions and binding energy in eV.

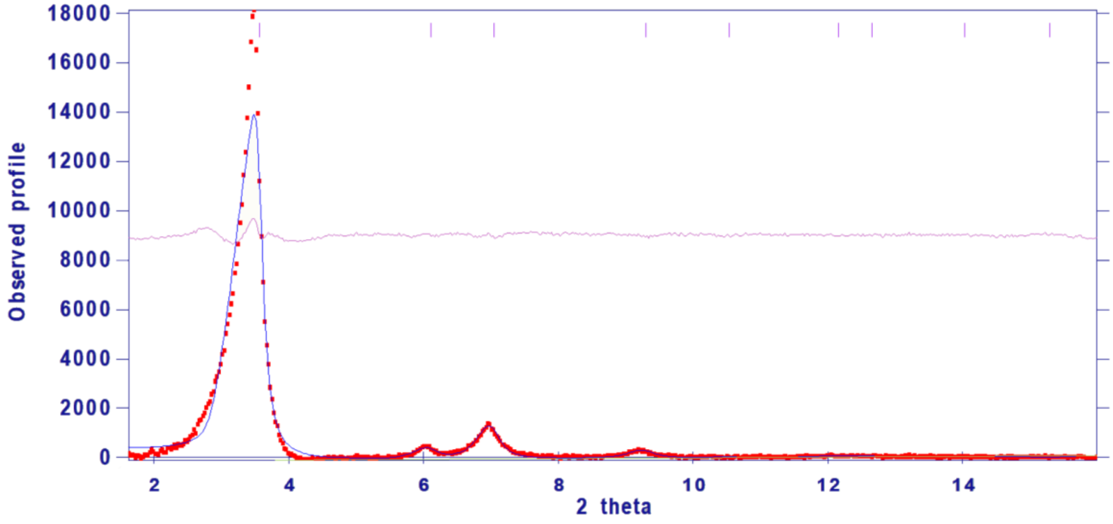


**Figure S12.** Pawley refinement of TFB-Phen.

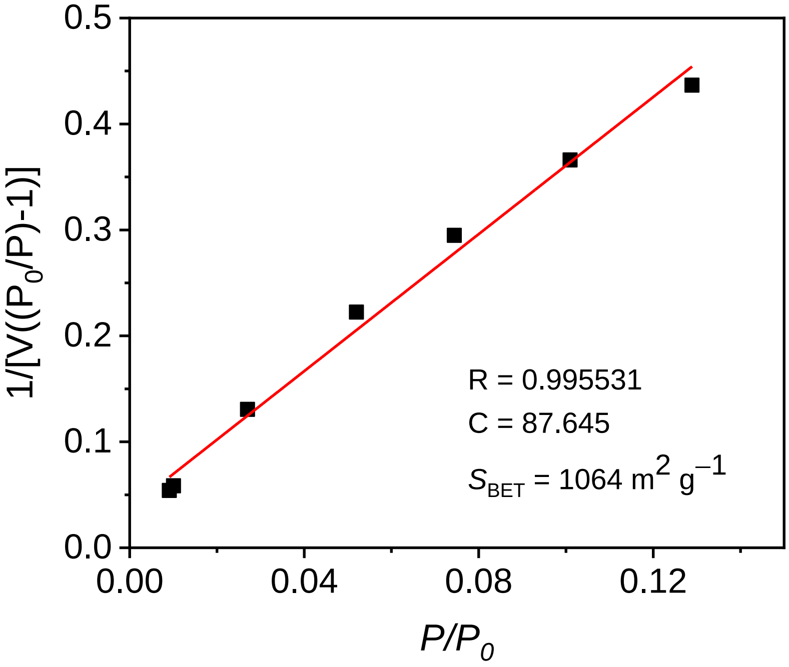


**Figure S13.** Multi-point BET plot and linear fit of TFB-Phen. Using BETSI software $S_{BET}$ = 904 m$^2$ g$^{-1}$ ($R^2$ = 0.995827, C = 219.354).

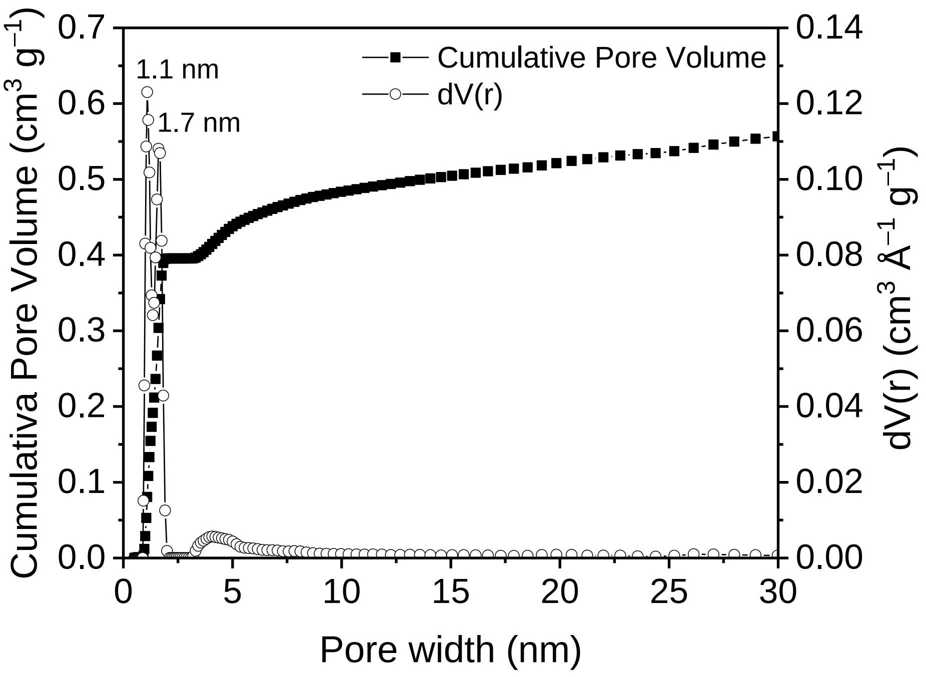


**Figure S14.** Pore size distribution (hollow spheres) and cumulative pore volume (filled spheres) profile of TFB-Phen. QSDFT model for slit/cylindrical pores, fitting error of 0.607%.

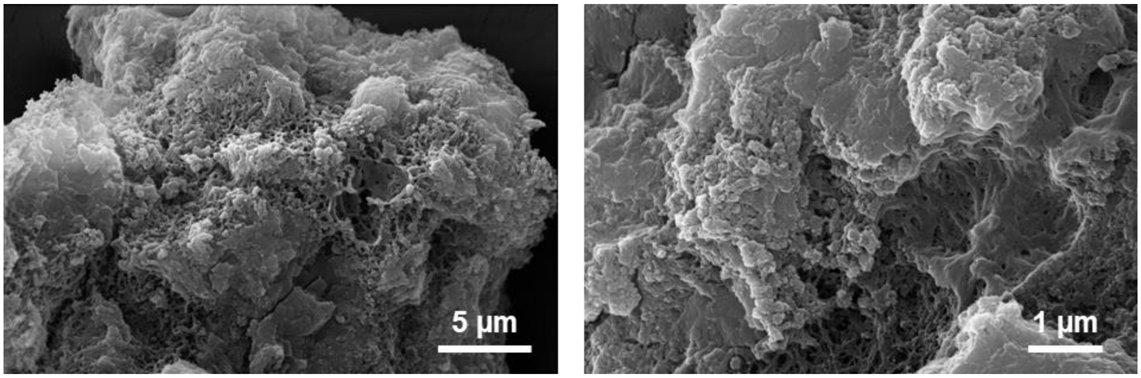


**Figure S15.** SEM micrographs of TFB-Phen.

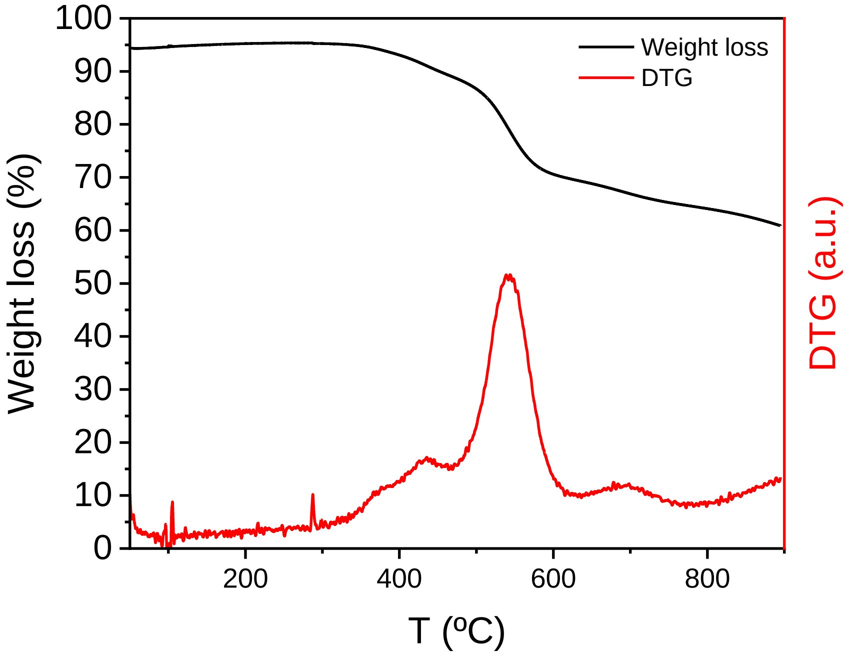


**Figure S16.** TGA and DTG of TFB-Phen.

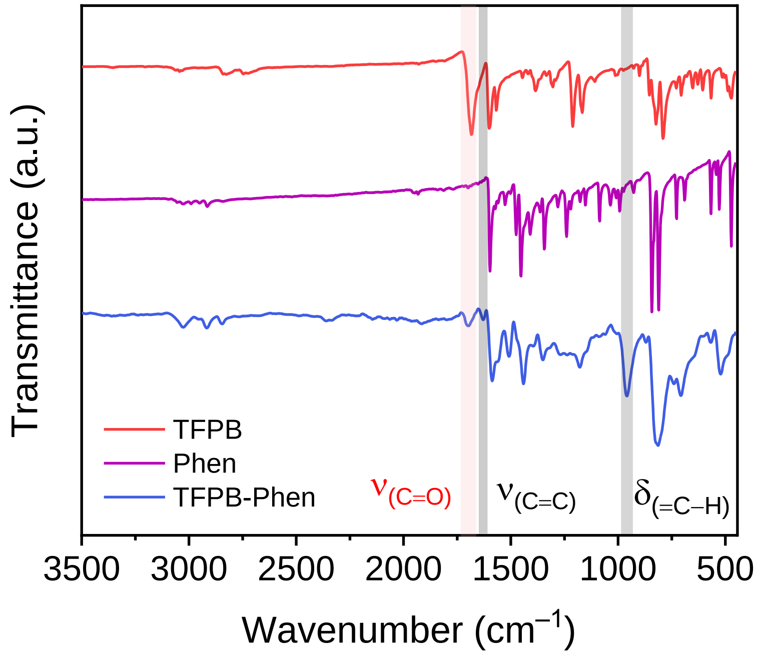


**Figure S17.** FT-IR spectra of TFPB-Phen and the respective monomers Phen and TFPB.

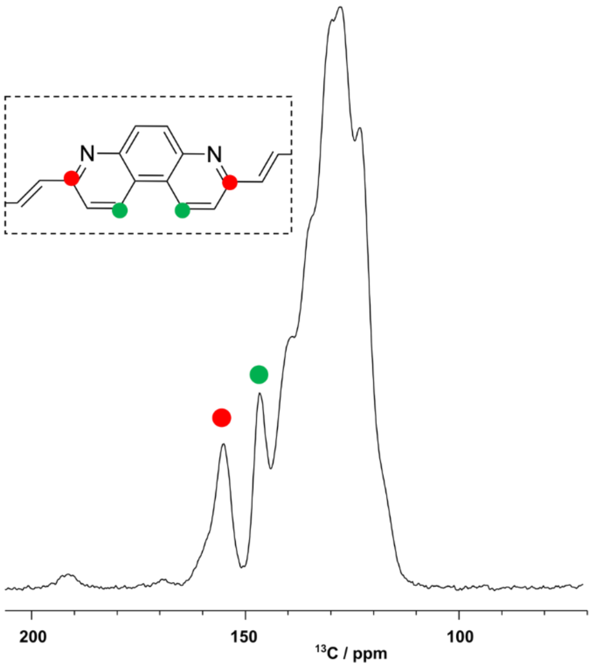


**Figure S18.** $^{13}C$ CP-MAS spectrum of TFPB-Phen, acquired at a MAS spinning rate of 12 kHz on a 9.4 T spectrometer. Simplified fragment of the Phen moiety is shown. The peaks between 110–140 ppm are attributed to the remaining aromatic carbons present in TFPB-Phen and the resonance at 190 ppm is ascribed to unreacted aldehyde moieties of TFPB.

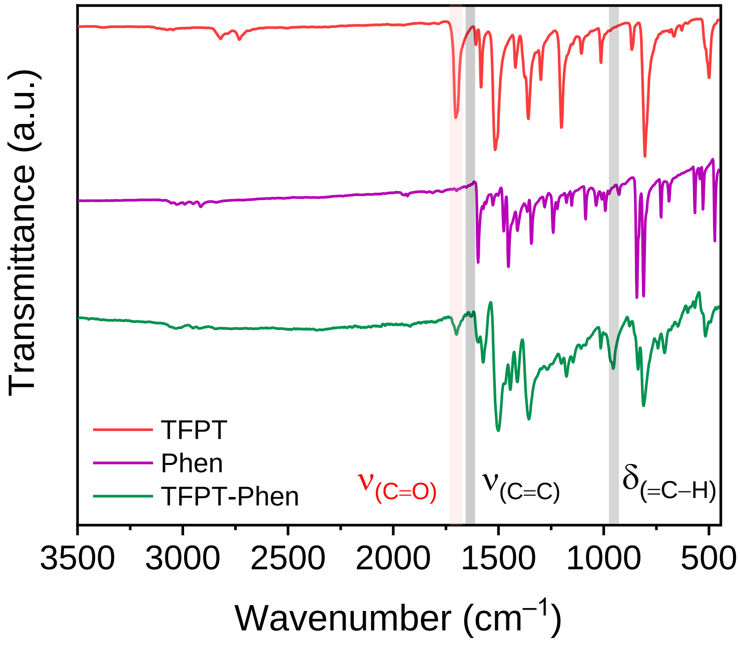


**Figure S19.** FT-IR spectra of TFPT-Phen and the respective monomers Phen and TFPT.

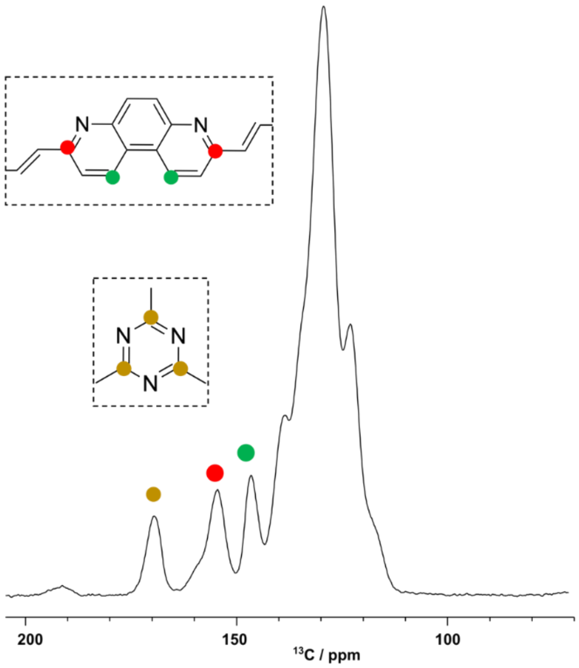


**Figure S20.** $^{13}$C CP-MAS spectrum of TFPT-Phen, acquired at a MAS spinning rate of 12 kHz on a 9.4 T spectrometer. Simplified fragments of some of the chemical functionalities within the structure are included. The peaks between 110–140 ppm are attributed to the remaining aromatic carbons present in TFPT-Phen and the resonance at 190 ppm is ascribed to unreacted aldehyde moieties of TFPT.

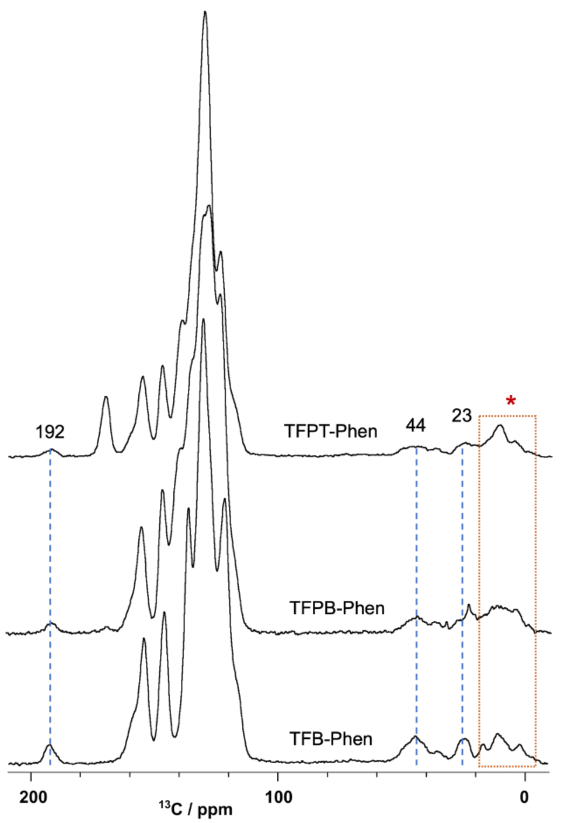


**Figure S21.** Full range $^{13}$C CP-MAS NMR spectra of the three Phen-COF samples. The peaks annotated at 192, 44, and 23 ppm can be ascribed to unreacted aldehyde and aliphatic carbons of the precursors. The peaks highlighted inside the orange square correspond to spinning sidebands.

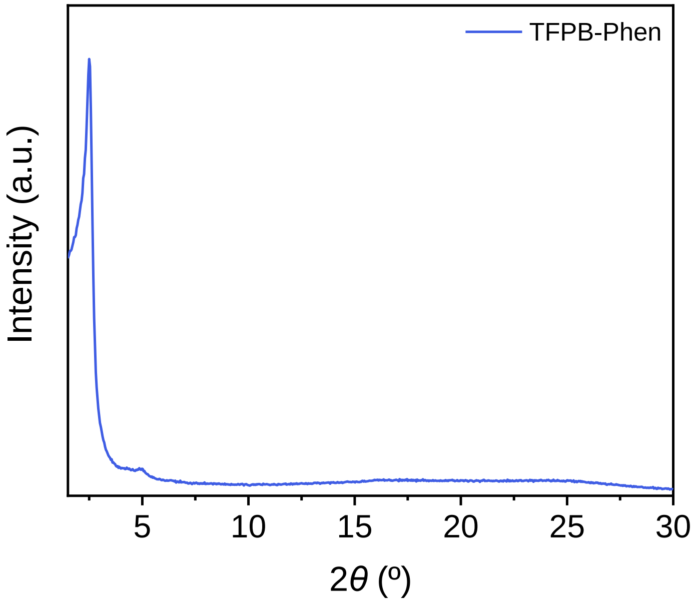


**Figure S22.** PXRD pattern of TFPB-Phen.

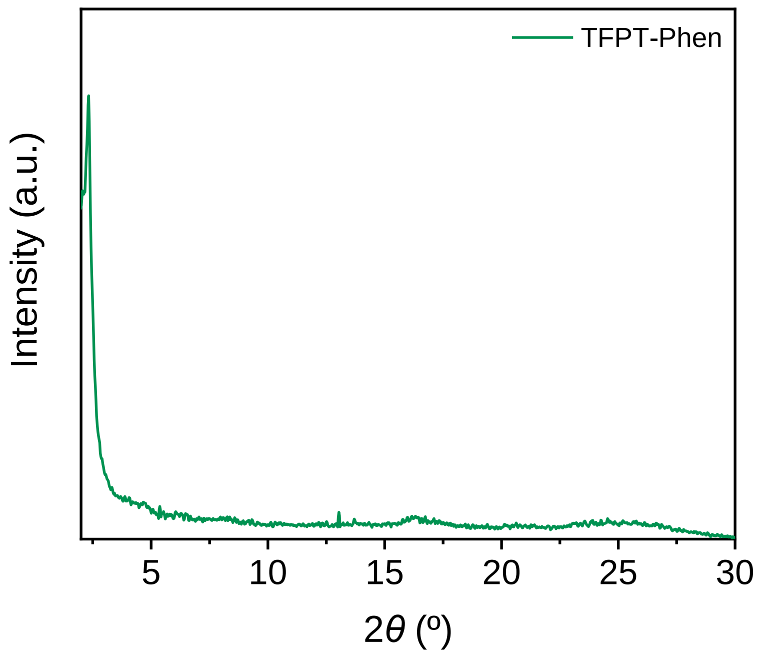


**Figure S23.** PXRD pattern of TFPT-Phen.

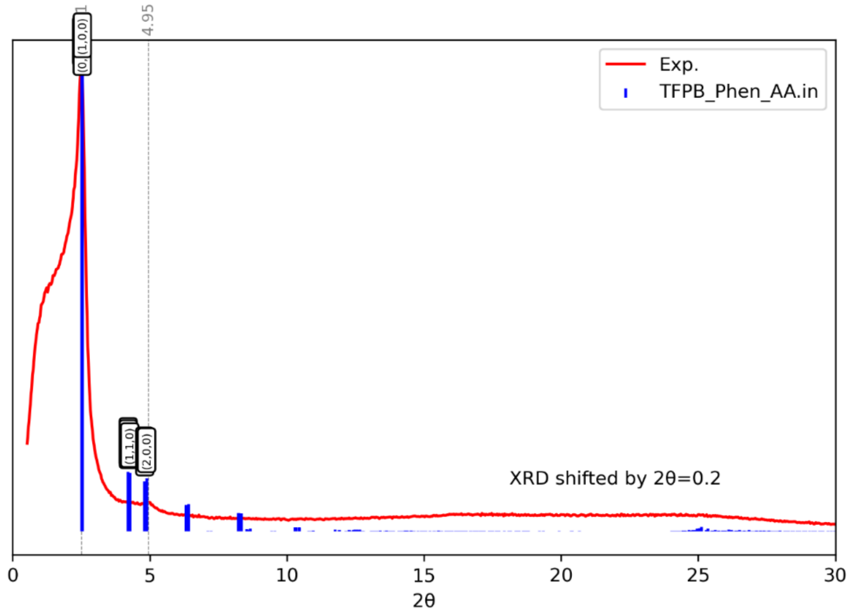


**Figure S24.** Simulated PXRD patterns of TFPB-Phen. The AA arrangement fits better with experimental pattern.

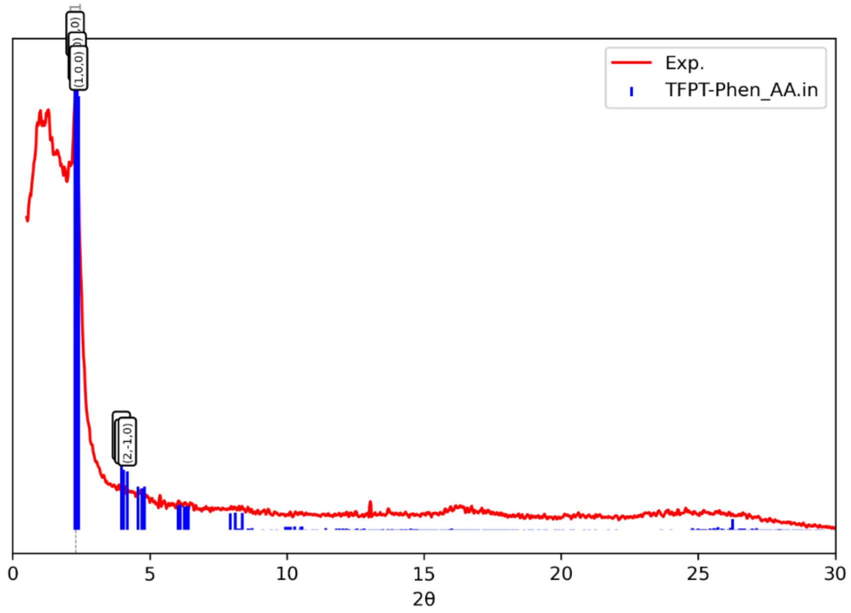


**Figure S25.** Simulated PXRD pattern of TFPT-Phen. The AA arrangement fits better with experimental pattern.

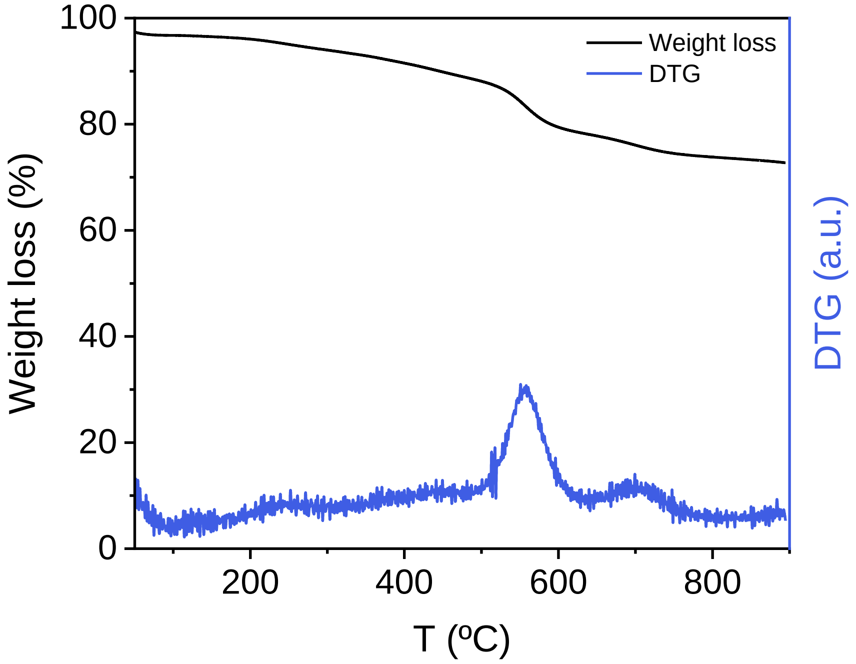


**Figure S26.** TGA and DTG profiles of TFPB-Phen.

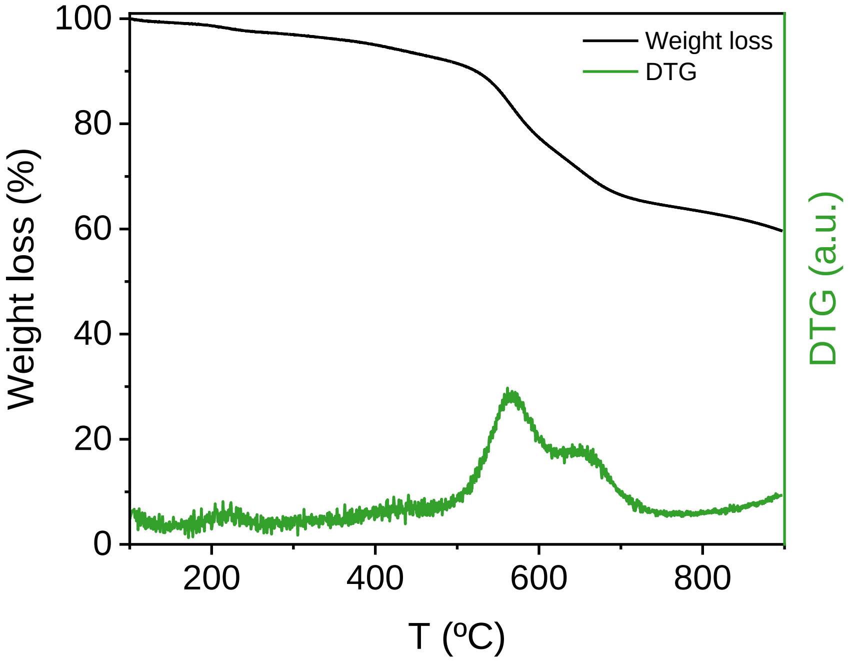


**Figure S27.** TGA and DTG profiles of TFPT-Phen.

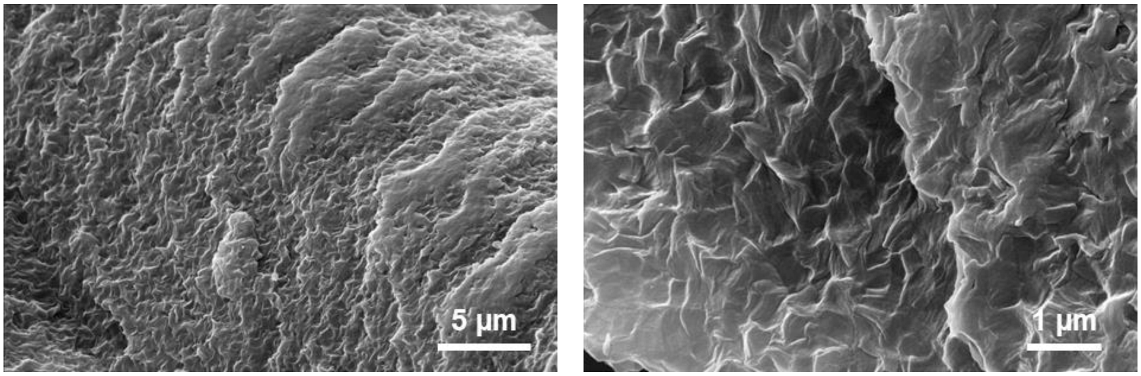


**Figure S28.** SEM micrographs of TFPB-Phen.

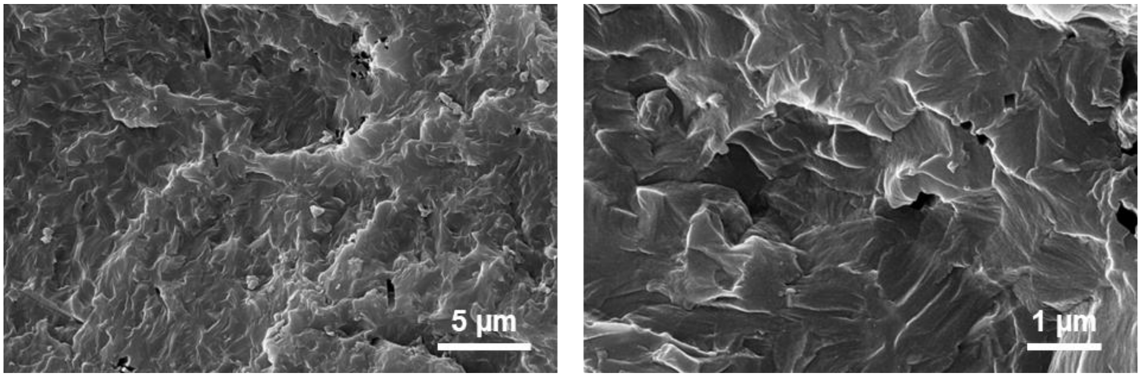


**Figure S29.** SEM micrographs of TFPT-Phen.

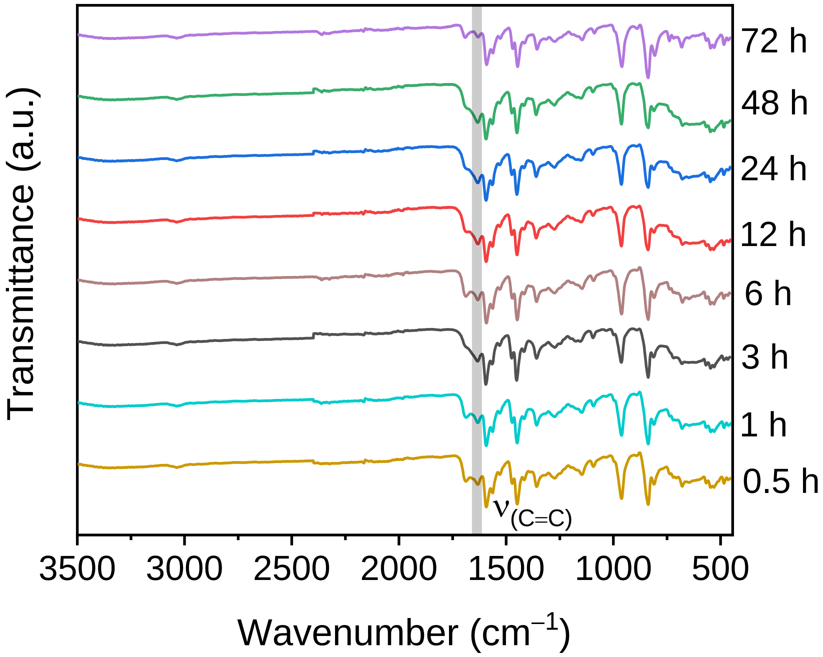


**Figure S30.** FT-IR spectra of TFB-Phen synthesized at different reaction times.

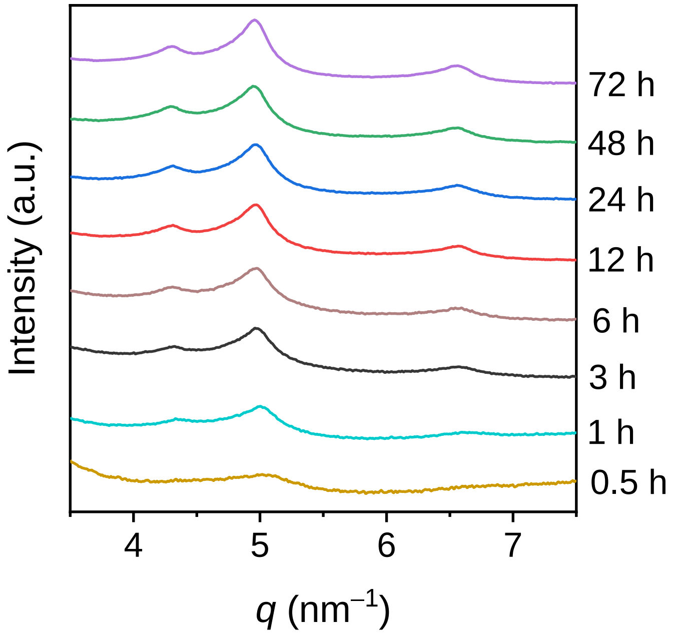


**Figure S31.** Close-up of the SAXS patterns of TFB-Phen synthesized at different reaction times in the 3.5–7.5 nm$^{-1}$ range.

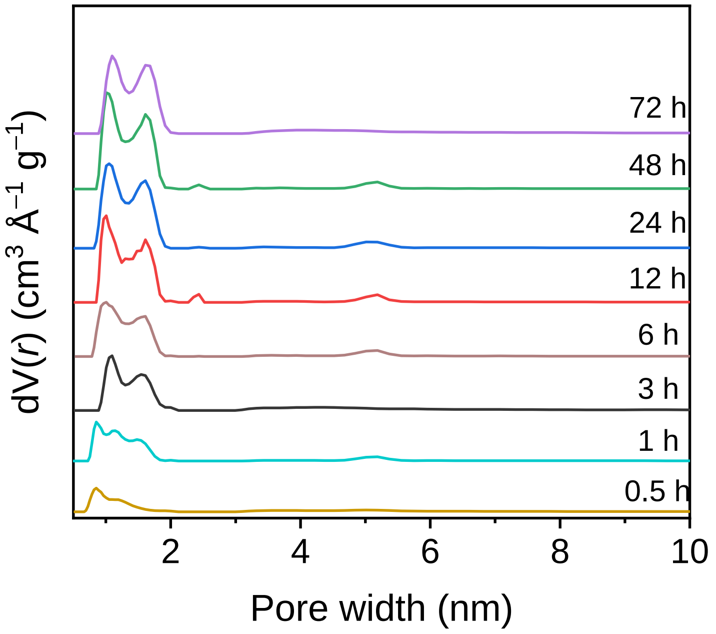


**Figure S32.** Pore size distribution of TFB-Phen synthesized at different reaction times. QSDFT model for slit/cylindrical pores, fitting error ranging from 0.3 to 1.0%.

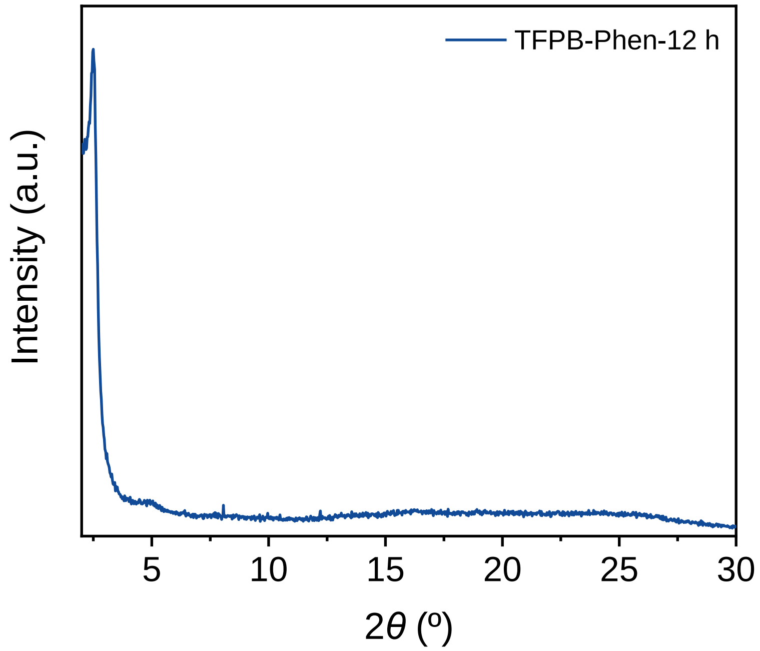


**Figure S33.** PXRD pattern of TFPB-Phen synthesized in 12 h.

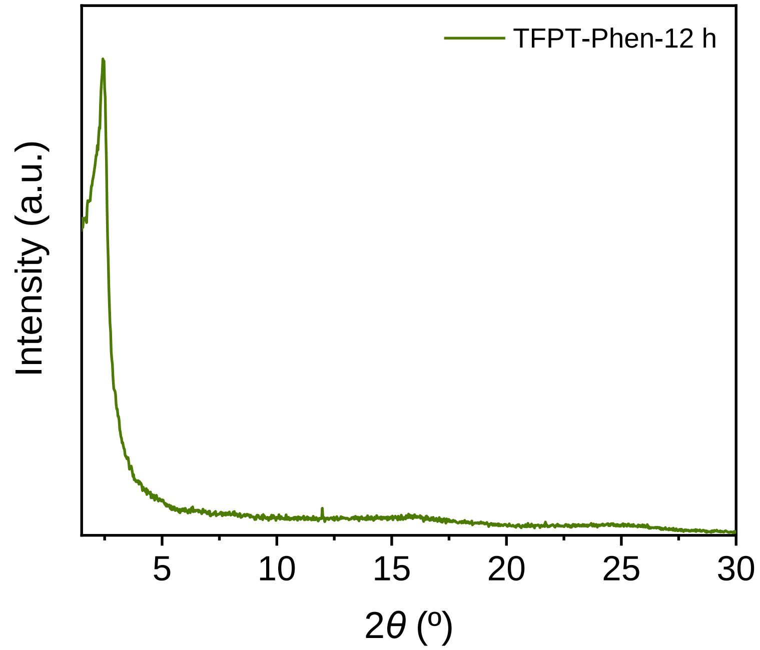


**Figure S34.** PXRD pattern of TFPT-Phen synthesized in 12 h.

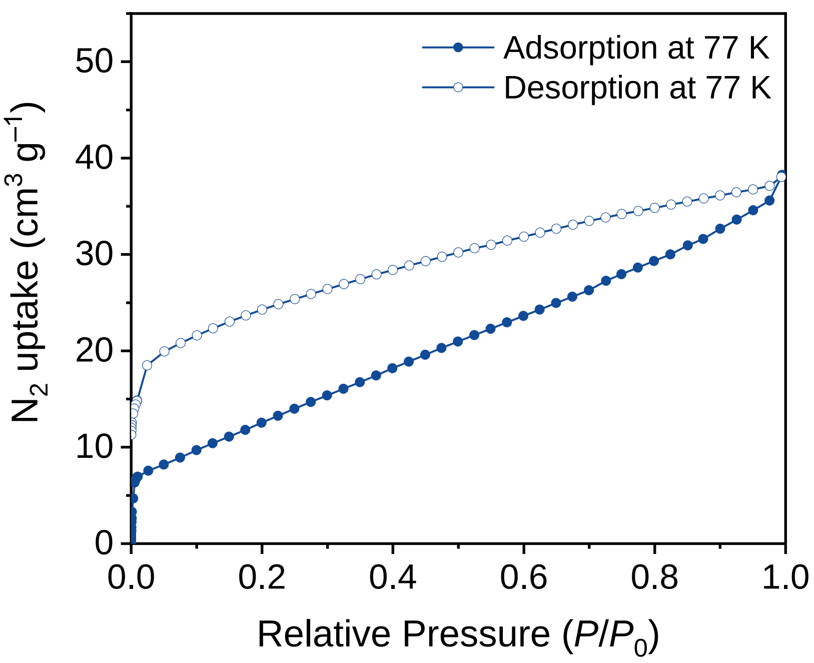


**Figure S35.** $N_2$ sorption isotherms of TFPB-Phen synthesized in 12 h.

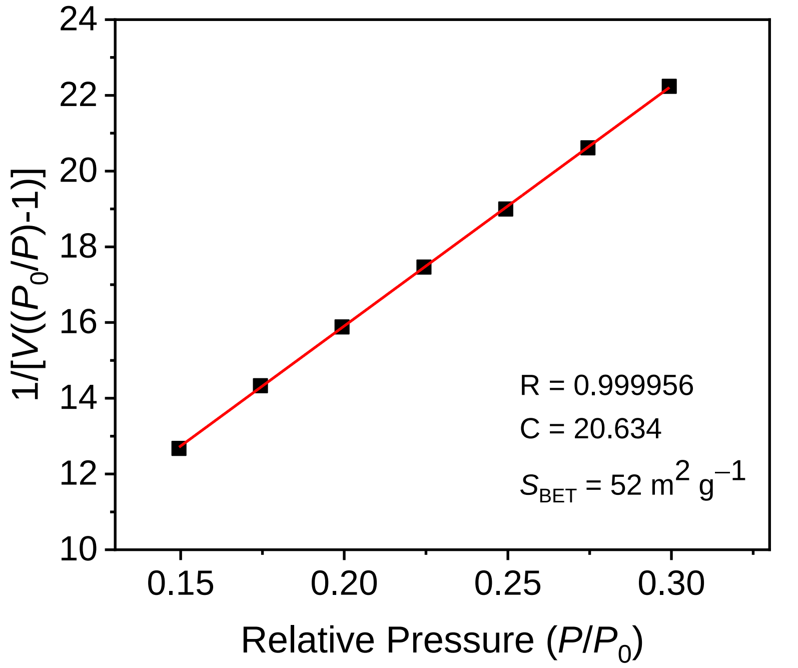


**Figure S36.** Multi-point BET plot and linear fit of TFPB-Phen synthesized in 12 h. Using BETSI software $S_{BET}$ = 51 $m^2$ $g^{-1}$ ($R^2$ = 0.999476, C = 25.053).

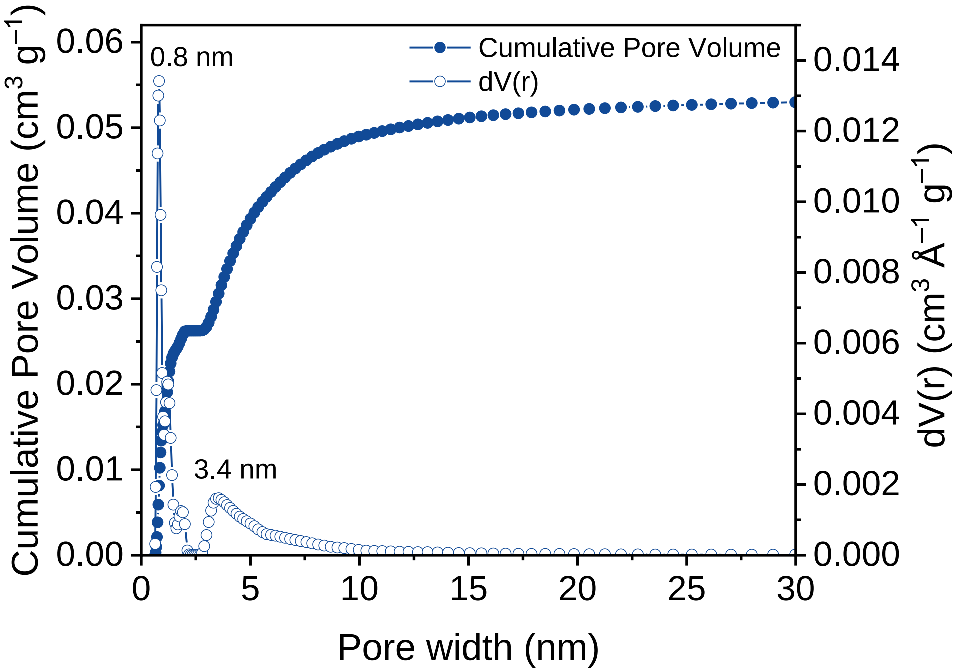


**Figure S37.** Pore size distribution (hollow spheres) and cumulative pore volume (filled spheres) profile of TFB-Phen. QSDFT model for slit/cylindrical pores, fitting error of 1.467%.

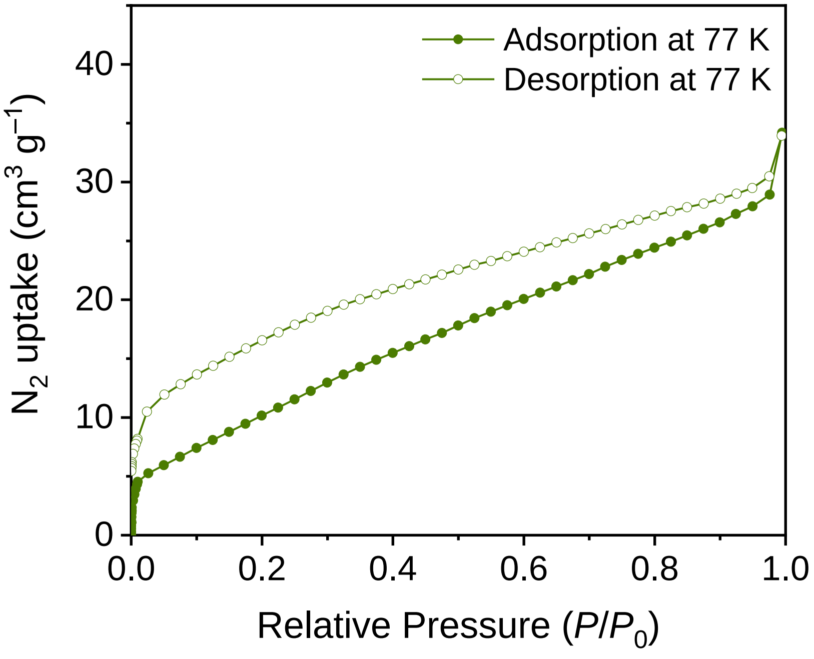


**Figure S38.** $N_2$ sorption isotherms of TFPT-Phen synthesized in 12 h.

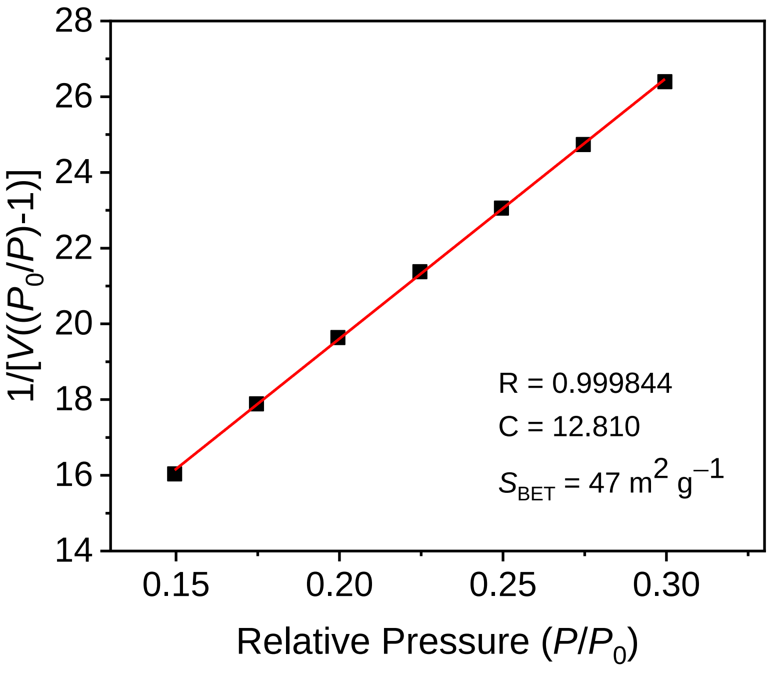


**Figure S39.** Multi-point BET plot and linear fit of TFPT-Phen synthesized in 12 h. Using BETSI software $S_{BET}$ = 46 m$^2$ g$^{-1}$ ($R^2$ = 0.999218, C = 13.739).

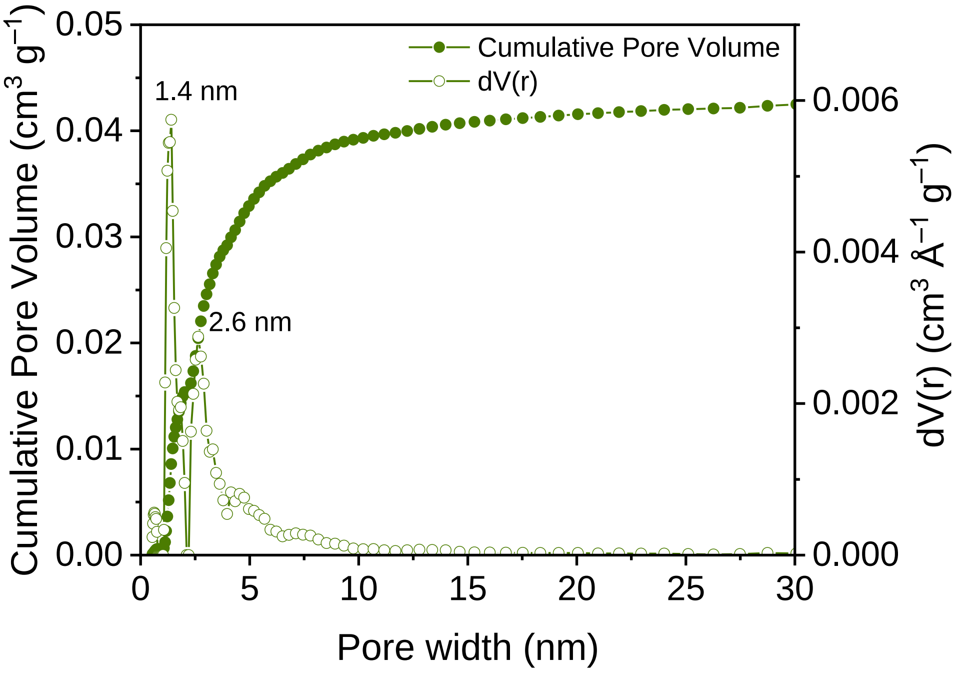


**Figure S40.** Pore size distribution (hollow spheres) and cumulative pore volume (filled spheres) profile of TFB-Phen. NLDFT model for slit pores, fitting error of 1.017%.

# 4. $^1$H NMR spectroscopy study on the formation of Phen model system

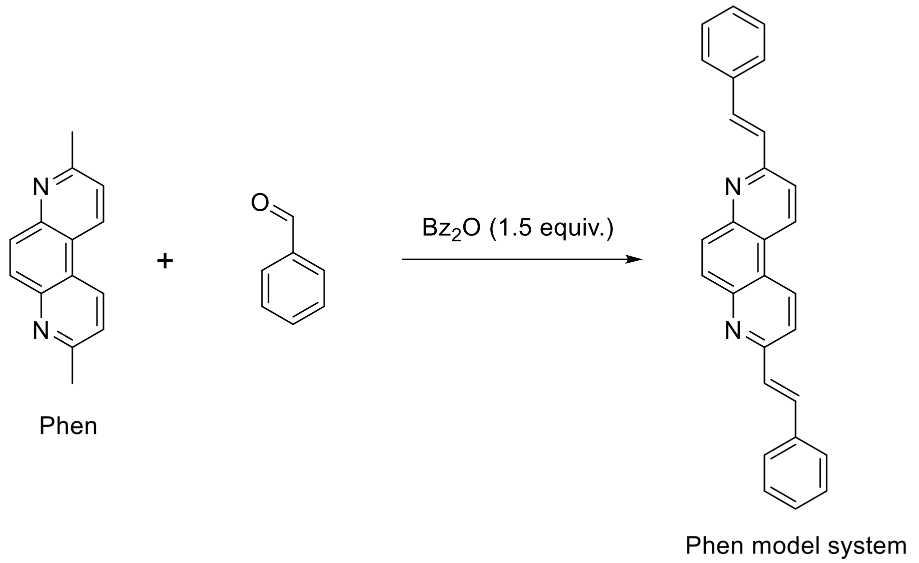


**Scheme S1.** Synthesis of Phen model system **1**.

In a 6 mL DURAN® culture tube, 3,8-dimethyl-4,7-phenanthroline (19.2 mg, 0.092 mmol, 1.0 equiv.) was placed with benzaldehyde (18.7 µL, 0.184 mmol, 2.0 equiv.) and benzoic anhydride (31.9 mg, 0.141 mmol, 1.5 equiv.) as catalyst under $N_2$ atmosphere. The reaction was sonicated 10 min before being placed in an oven at 180 ºC for 0.5, 1, 3, 6, or 24 hours. Then, the reaction mixture was left to cool down to room temperature and neutralized with aq. 1 M NaOH solution during 20 min. A liquid–liquid extraction was performed with $CH_2Cl_2$, the organic phase was collected, evaporated to dryness, and subjected to analysis by NMR spectroscopy and mass spectrometry.

Mass spectra was acquired after all the tests, the most relevant was at 6 h: HR-ESI-MS (6 h): *m/z* (%): 385.1698 (100, $[M+H]^+$, calculated for $C_{28}H_{21}N_2^+$: 385.1699), 681.3011 (19.2, $[M+H]^+$, calculated for $C_{49}H_{37}N_4^+$: 681.3013) (see also Figure S42).

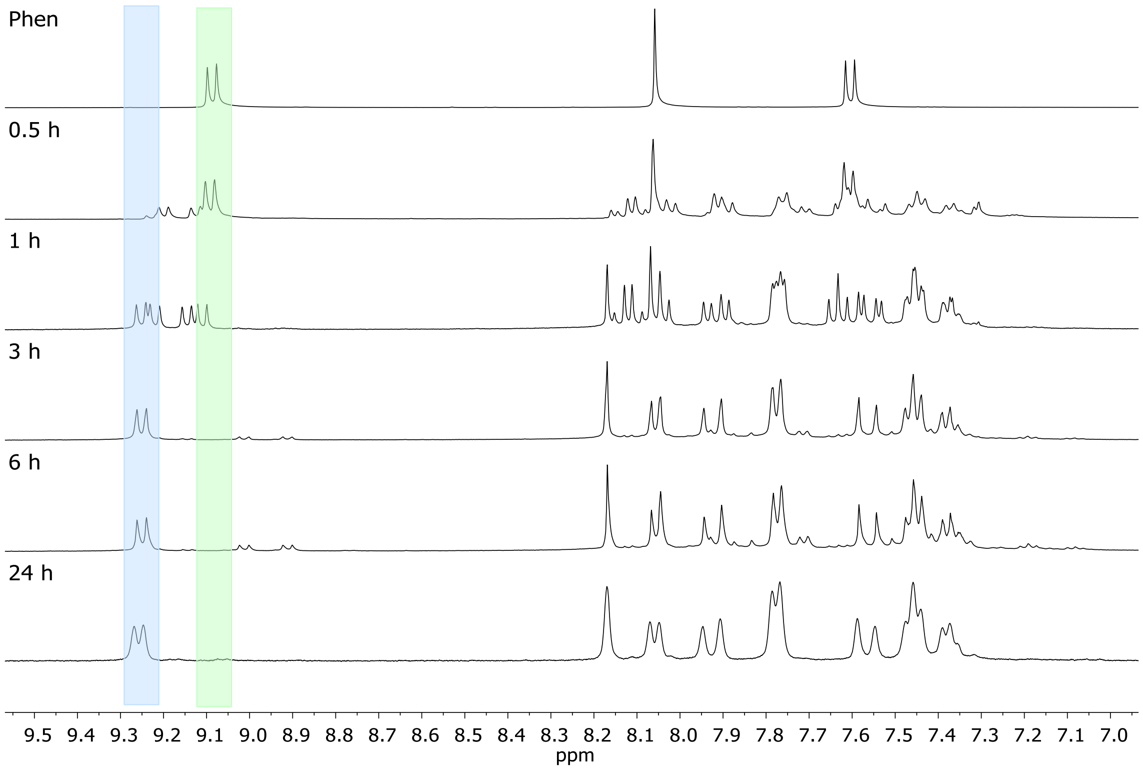


**Figure S41.** $^1$H NMR (400 MHz, $(CD_3)_2SO$) spectroscopy study on the formation of the Phen model system **1** over time. Comparation with the initial Phen spectrum and that of the model system synthesized in 24 h and purified by column chromatography.

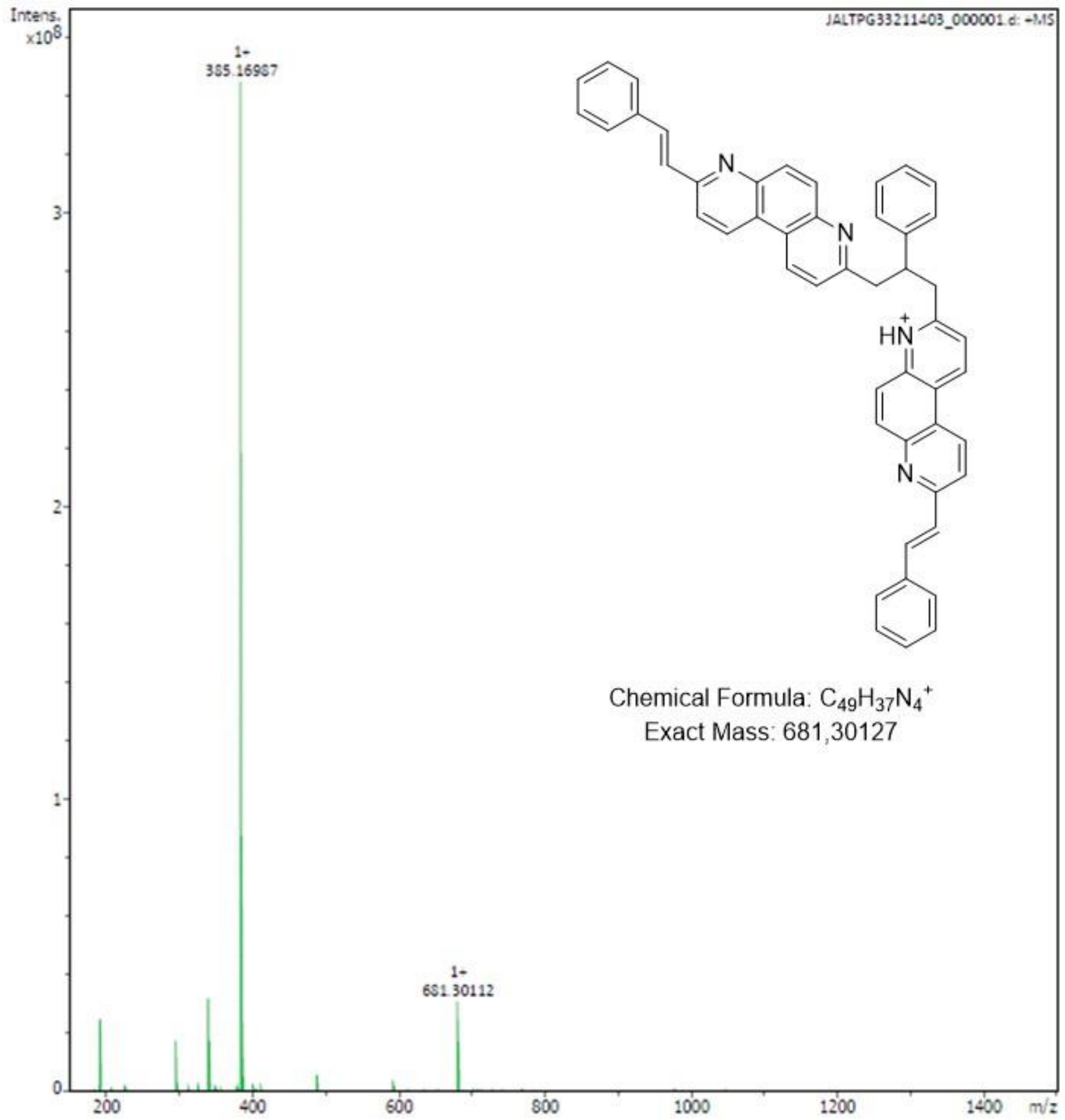


**Figure S42.** Mass spectrum with the proposed structure of an intermediate detected during the synthesis of the Phen model system at 3 h of reaction time.

**Synthesis of Py model system**

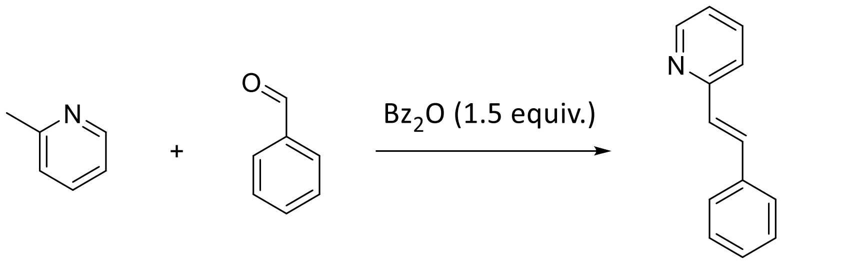


**Scheme S2.** Synthesis of Py model system.

In a 6 mL DURAN® culture tube, 2-methylpyridine, Py, (200.0 mg, 2.15 mmol, 1.0 equiv.) was placed with benzaldehyde (220.7 μL, 2.15 mmol, 1.0 equiv.) and benzoic anhydride (744.5 mg, 3.29 mmol, 1.5 equiv.) as catalyst under $N_2$ atmosphere. The reaction was sonicated 10 min before being placed in an oven at 180 ºC overnight. Then, the reaction mixture was left to cool down to room temperature and neutralized with aq. 1 M NaOH solution for 20 min. A liquid–liquid extraction was performed with $CH_2Cl_2$, the organic phase was collected and evaporated to dryness. Purification by column chromatography ($SiO_2$; $CH_2Cl_2$/hexane 1:1) gave Py model system as white solid (222 mg, 57% yield).

**$^1$H NMR (400 MHz, $(CD_3)_2SO$):** 8.57 (d, *J* = 4.8 Hz, 1H), 7.79 (t, *J* = 7.7 Hz, 1H), 7.72–7.63 (m, 3H), 7.55 (d, *J* = 7.9 Hz, 1H), 7.41 (t, *J* = 7.5 Hz, 2H), 7.36–7.22 (m, 3H).

**Reversibility of Py and Phen model systems**

a)

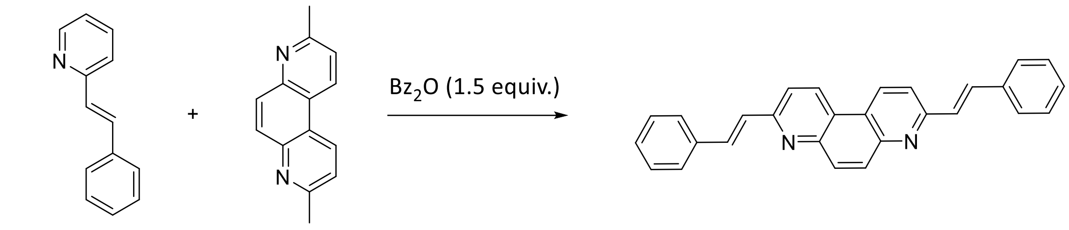


Py model system　Phen　Phen model system

b)

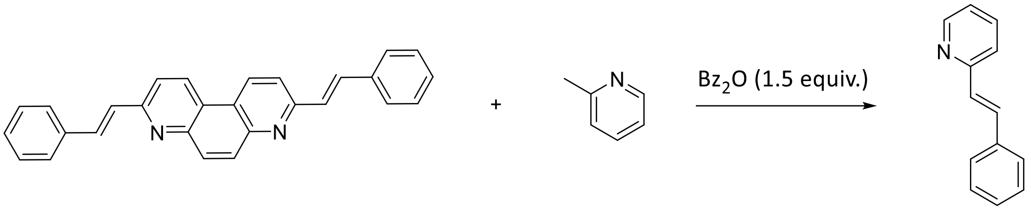


Phen model system　Py　Py model system

**Scheme S3.** Study of the reversibility of a) Py model system with Phen and b) Phen model system with Py.

In a 6 mL DURAN® culture tube, 0.2 mmol of the corresponding model system were reacted with 0.2 mmol of Phen or Py in the presence of 0.3 mmol of $Bz_2O$ as catalyst under $N_2$ atmosphere. The reactions were sonicated 10 min before being placed in an oven at 180 ºC for 16 h. Then, the reaction mixtures were left to cool to room temperature and neutralized with aq. 1 M NaOH solution during 20 min. A liquid–liquid extraction was performed with $CH_2Cl_2$, the organic phase was collected and evaporated to dryness. Both reactions were also carried out in the presence of 1 equiv. of water.

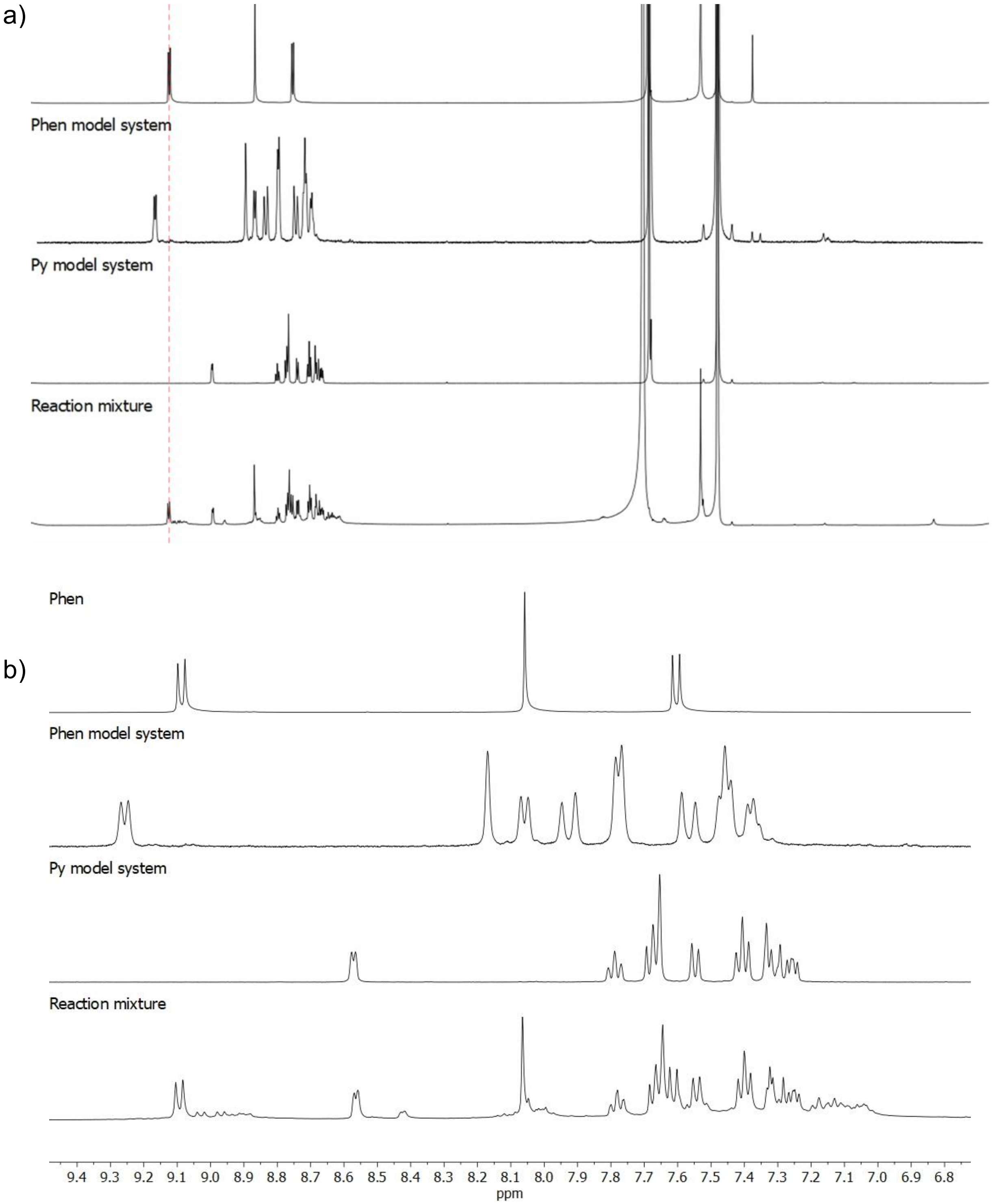


**Figure S43.** a) $^{1}$H NMR (400 MHz, $(CD_3)_2SO$) spectroscopy study on the formation of the Phen model system from the Py model system: from top to bottom Phen, Phen model system, Py model system, and the reaction mixture after 16 h after work-up. b) Zoom of the aromatic region.

a)

b)

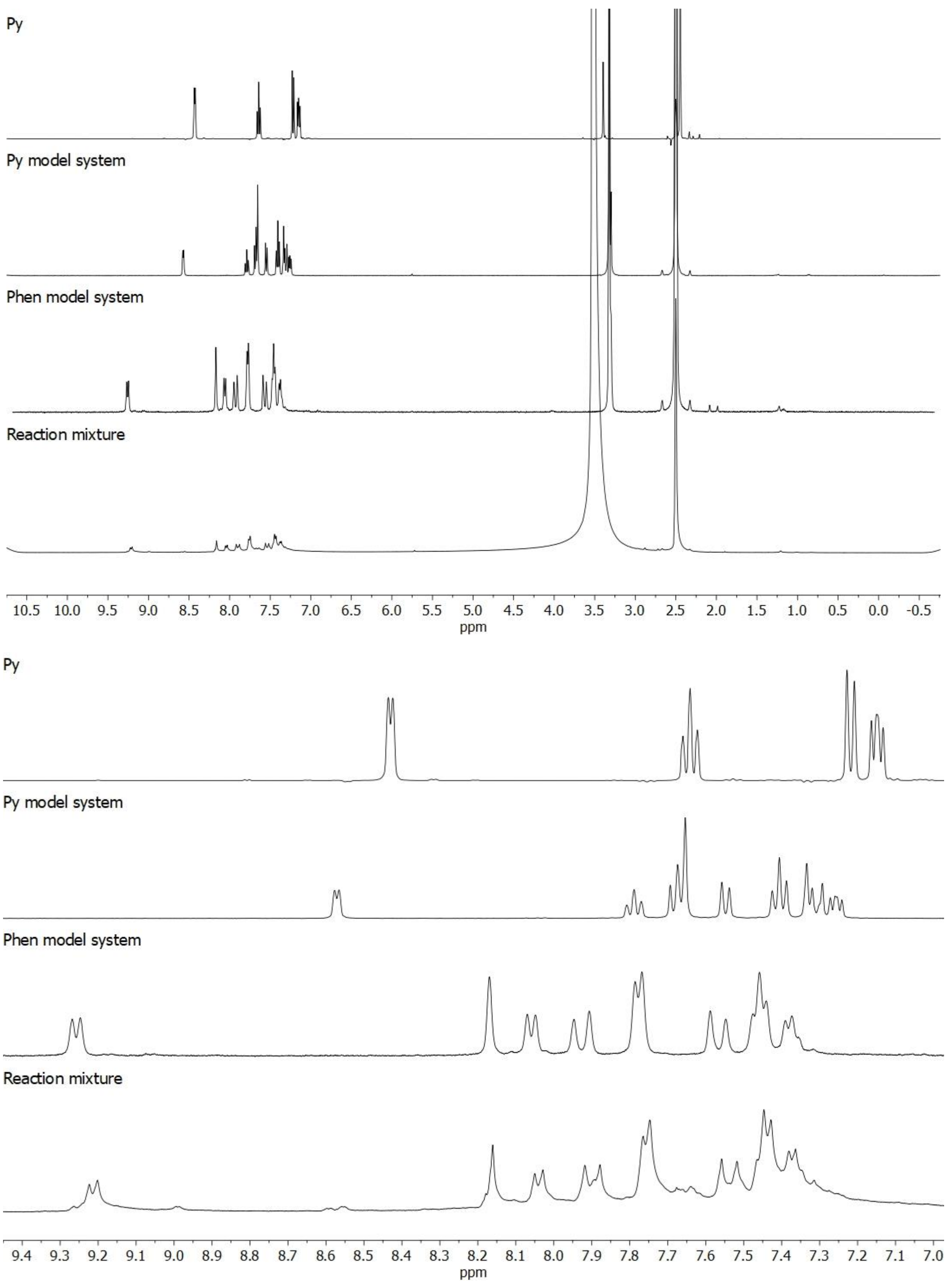


**Figure S44.** a) $^1$H NMR (400 MHz, $(CD_3)_2SO$) spectroscopy study on the formation of the Py model system from the Phen model system: from top to bottom Py, Py model system, Phen model system, and the reaction mixture after 16 h after work-up. b) Zoom of the aromatic region.

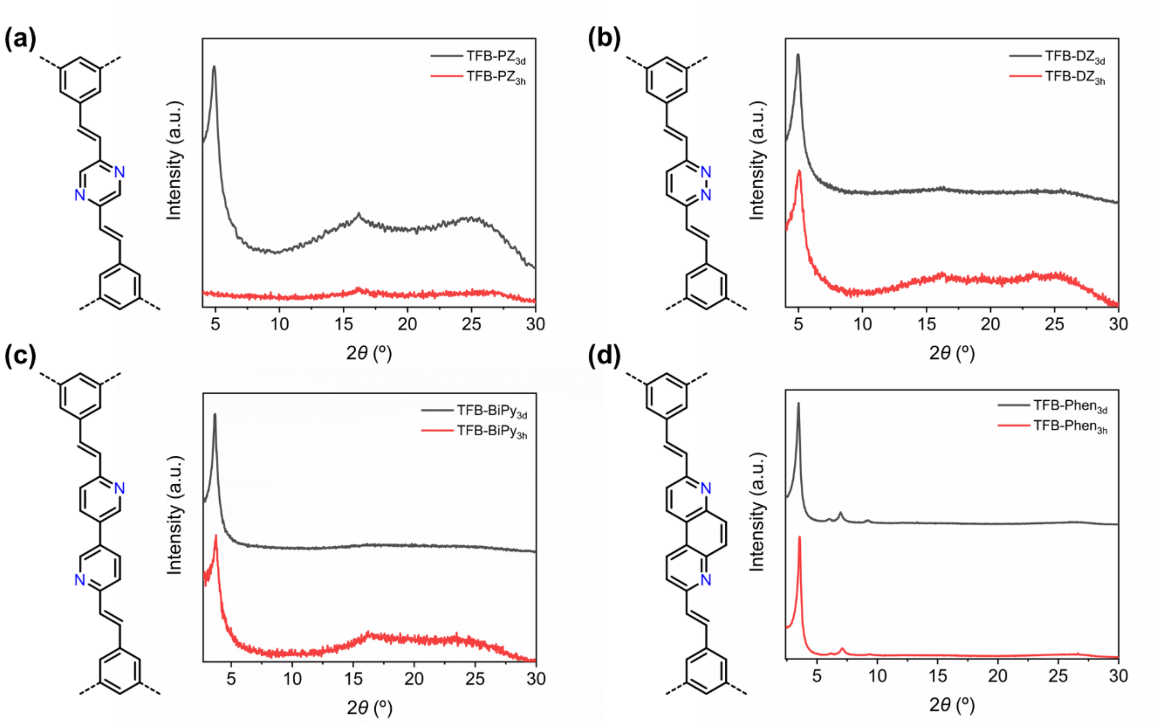


**Figure S45.** PXRD patterns after 3 h (red) and 3 d (grey) of reaction of TFB with (a) 2,5-dimethylpyrazine (PZ), (b) 3,6-dimethylpyridazine (DZ), (c) 6,6'-dimethyl-3,3'-bipyridine (BiPy) and (d) Phen, respectively.

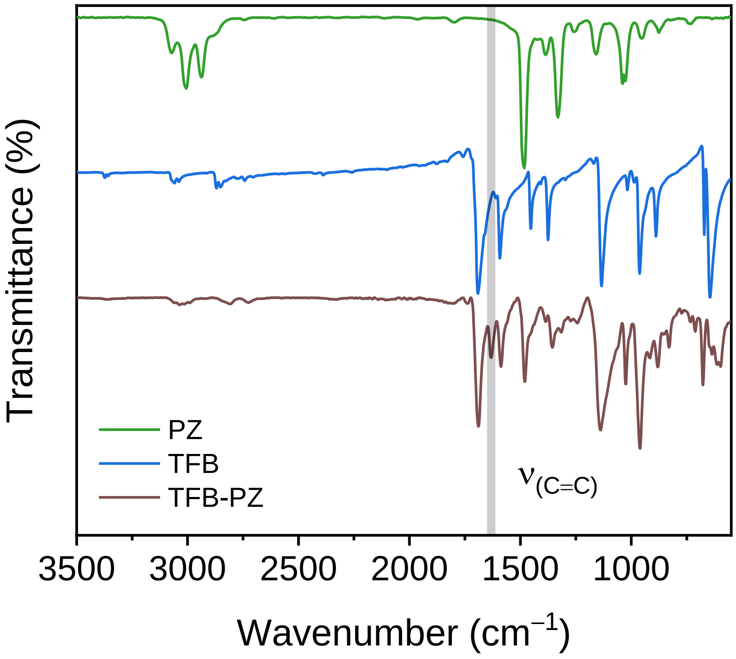


**Figure S46.** FT-IR spectra of PZ, TFB, and TFB-PZ synthesized in 3 h.

# 5. Calculations on the model system

Calculations were carried out with Gaussian 16 employing the B97D functional, which accounts for dispersion corrections together with the 6-31++g(d,p) basis set for mechanistic studies and the 6-311++g(2d,2p) 6d basis set to obtain stacking Gibbs energies.

### Evaluation of the reaction mechanism of the model system

The reaction starts with the activation of Phen by reaction with $Bz_2O$ as proposed earlier,[4] leading to the cationic species **2**, where the hydrogens of the closest methyl group become more positively charged, making deprotonation by $BzO^-$ easier. For comparison, deprotonation of the other methyl group at **2** (dashed curved arrow) was computed to be $\Delta G$ = 8.7 kcal/mol, being 20.6 kcal/mol less favorable. In the next step, **3** adds to the carbonyl group of the activated aldehyde leading to **4**, which after water elimination gives rise to the cationic species **5**. A second sequential deprotonation (less favorable than the first one), addition to the activated aldehyde and water elimination process finally leads to the final product **9**, recovering the catalyst.

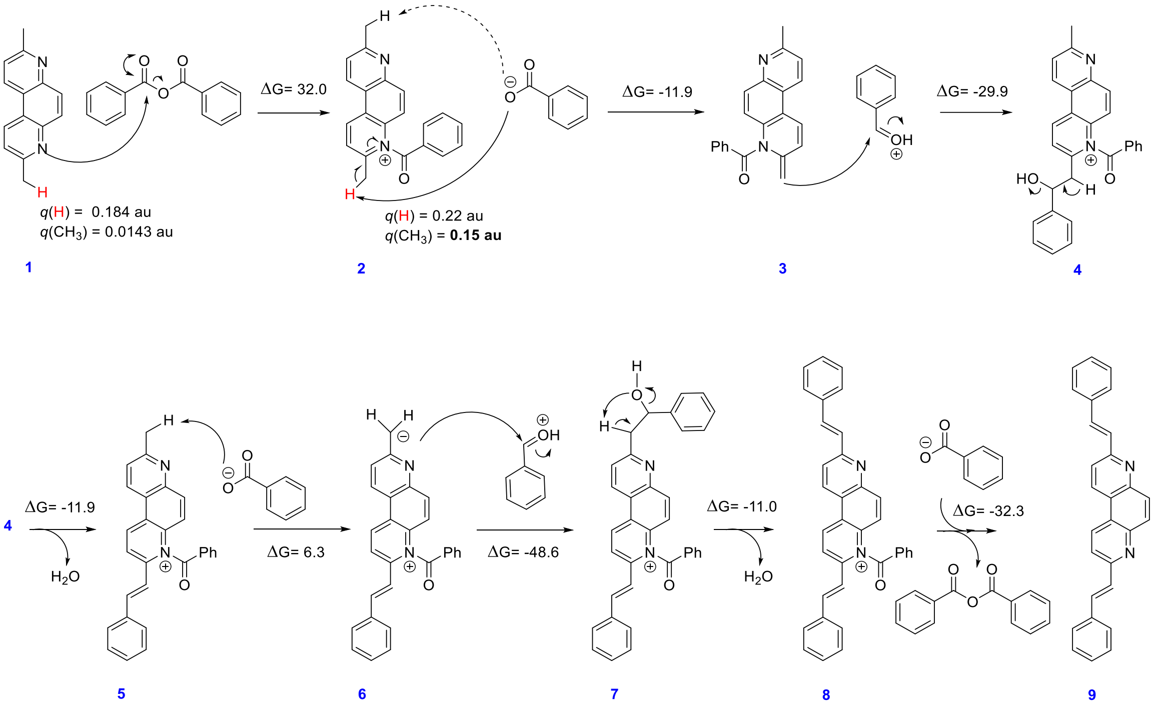


**Scheme S4.** Proposed mechanism for the formation of Phen model system.

## Evaluation of the crystallization process

The stacking interaction between monomers of the Phen model system were computed considering both the parallel and the antiparallel arrangement between monomers. Three Phen conformers (Figure S47) were considered. According to the values obtained, the most stable conformer of Phen is A1, displaying the largest N···H1 intramolecular distance, although the other conformers could also be present in the mixture.

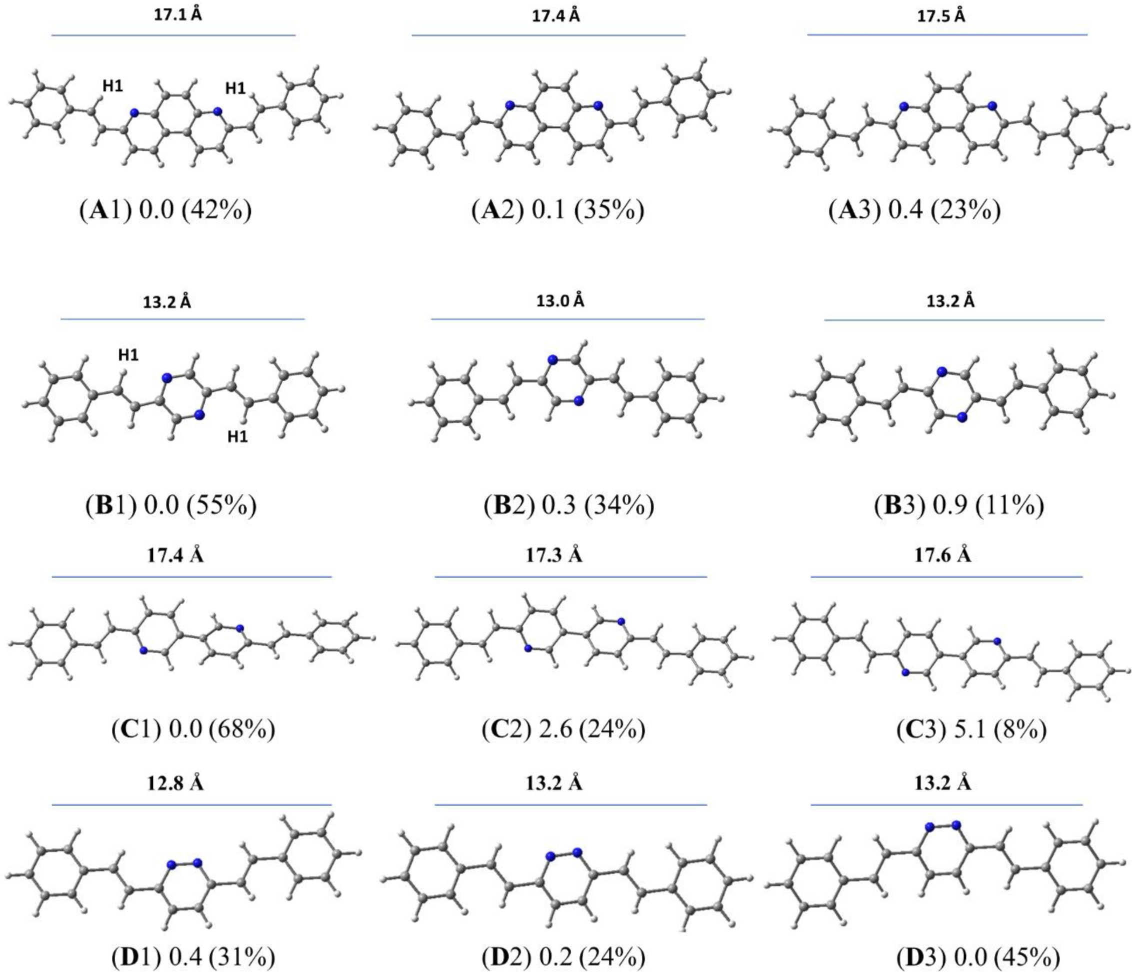


**Figure S47.** Possible conformers of Phen (A), pyrazine (B) bipyridine (C), and pyridazine (D). Relative Gibbs energy values in kcal·mol$^{-1}$ and percentage of relative population in brackets. It is noted that for bipyridine, other three conformers with nitrogen atoms in the same side, respectively, **C**4 ($\Delta G$=0.42 (31%)), **C**5 ($\Delta G$= 3.0 (11%)) and **C**6 ($\Delta G$= 5.7 (4%)) can be located but are not shown.

A1–A1, A2–A2, and A3–A3 dimers of three Phen model system were then constructed considering the stacking in parallel and antiparallel arrangements. An analogous study was carried out with the pyrazine and bipyridine model systems for comparison. First, the possible conformers of the model systems (Figure S47) were explored and then the stacking interactions of the dimers were considered. The most stable dimers are shown in Figure S48, highlighting the favorable nature of the Phen stacking interactions.

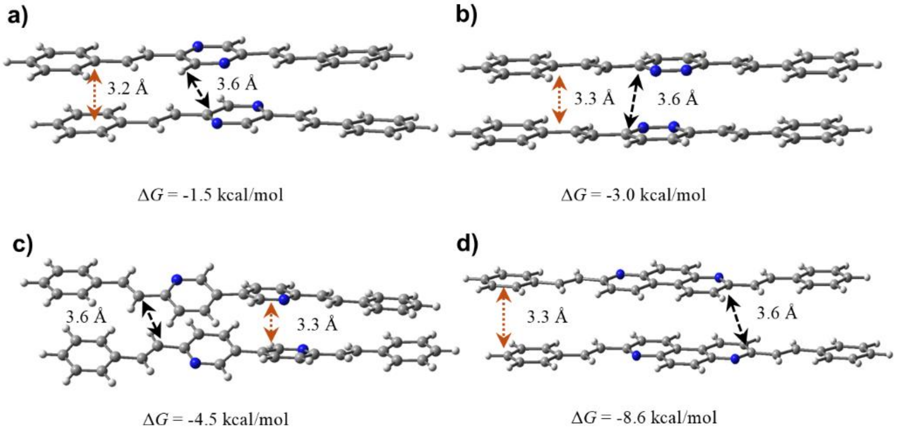


**Figure S48.** Example of dimers formed by self-assembly of a) pyrazine, b) pyridazine c) bipyridine, and d) Phen model systems. Stacking Gibbs energy together with intralayer and intermolecular distances are shown.

# 6. Dipole moments

Dipole moments were computed and validated against published values for simple molecular analogues of the COF linkers studied experimentally. The dipole moment was obtained at the PBE-MBD/light level, same as used in the COF models. For 3,3'-bipyridine, a full conformational search was performed revealing two almost isoenergetic conformers populated at room temperature with different dipole moments module and orientation, see Figure S49 and Table S5 and main text for discussion.

**Table S5.** Dipole moments of the different nitrogenated molecular analogues computed at the PBE-MBD/light level together with the literature-reported values.

| Molecule | Dipole moment (D) | |
|---|---|---|
| | **Computed** | **Literature** |
| Phen | 3.44 | 3.63[5] |
| BiPy conformation 1 | 1.33 | Not found |
| BiPy conformation 2 | 3.61 | Not found |
| pyridazine | 4.20 | 3.90[6], 4.22[7] |

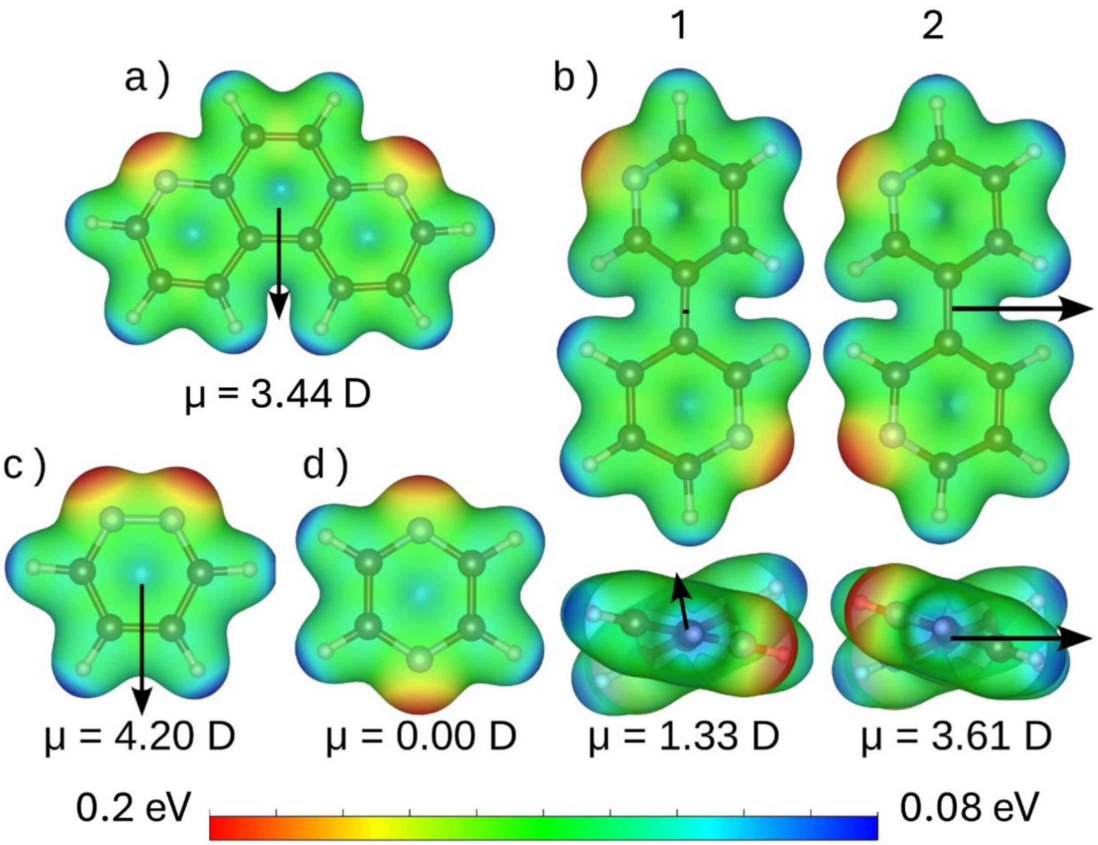


**Figure S49.** Calculated electrostatic potential mapped on an isodensity surface of 0.02 e/Bohr³ in eV and scaled dipole moments represented as arrows (1D = 1 Å) from PBE-MBD/light level calculations of: a) Phen, b) two conformations of 3,3'-Bipyridine, c) Pyridazine, and d) Pyrazine.

## 7. Optical and electronic properties

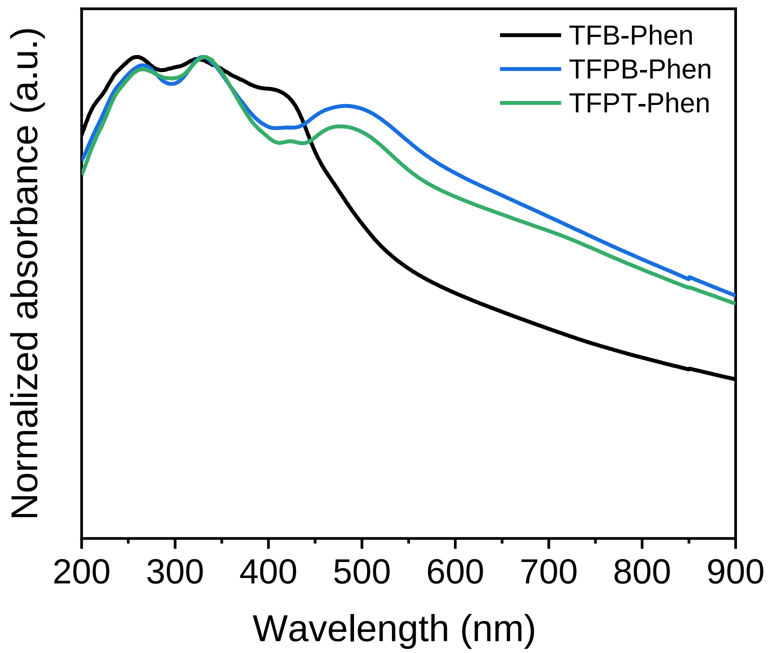


**Figure S50.** UV-vis DR spectra of TFB-Phen (black), TFPB-Phen (blue), and TFPT-Phen (green).

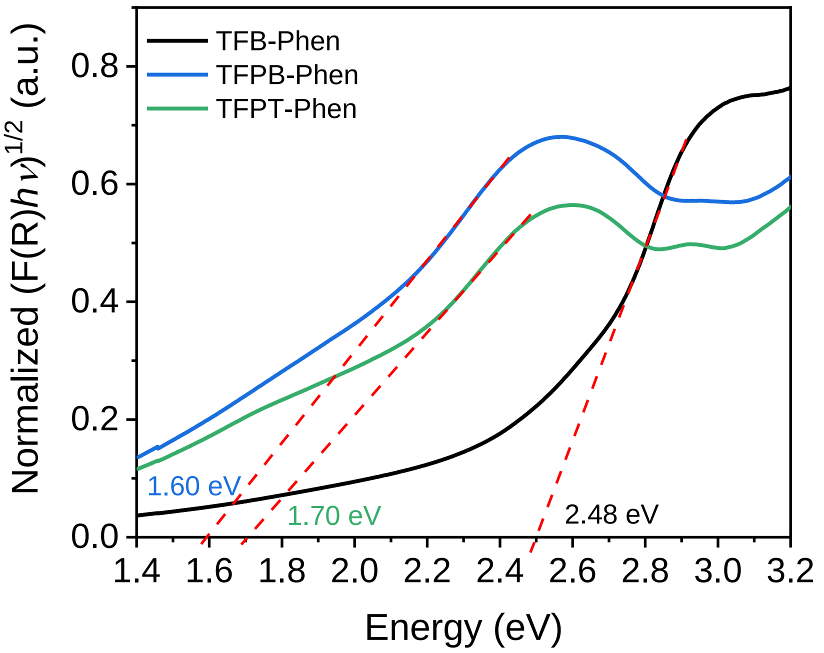


**Figure S51.** Optical band gaps determined from the K–M transformed reflectance spectra of TFB-Phen (black), TPPB-Phen (blue), and TFPT-Phen (green).

## Band Structure and band gap computation

To accurately calculate the band gaps, we used B3LYP functional, as it is well known to produce more realistic band gaps. Band structure and corresponding Density of States (DOS) of computed Phen-COFs are presented in Figure S52, while Table S6 shows the valence (HOCO) and conduction (LUCO) band energies.

**Table S6.** Valence and conduction band energies and gaps at the B3LYP/light level on a 3x3x1 and 3x3x4 k-point grid for monolayer and bulk structures respectively for the computed COFs.

| Material | Monolayer | | | Bulk | | |
|---|---|---|---|---|---|---|
| | Valence | Conduction | Band gap | Valence | Conduction | Band gap |
| TFB-Phen | -5.996 | -2.821 | 3.175 | -6.092 | -3.405 | 2.687 |
| TFPB-Phen | -5.857 | -2.722 | 3.135 | -5.795 | -2.931 | 2.864 |
| TFPT-Phen | -6.085 | -3.090 | 2.995 | -5.995 | -3.355 | 2.640 |

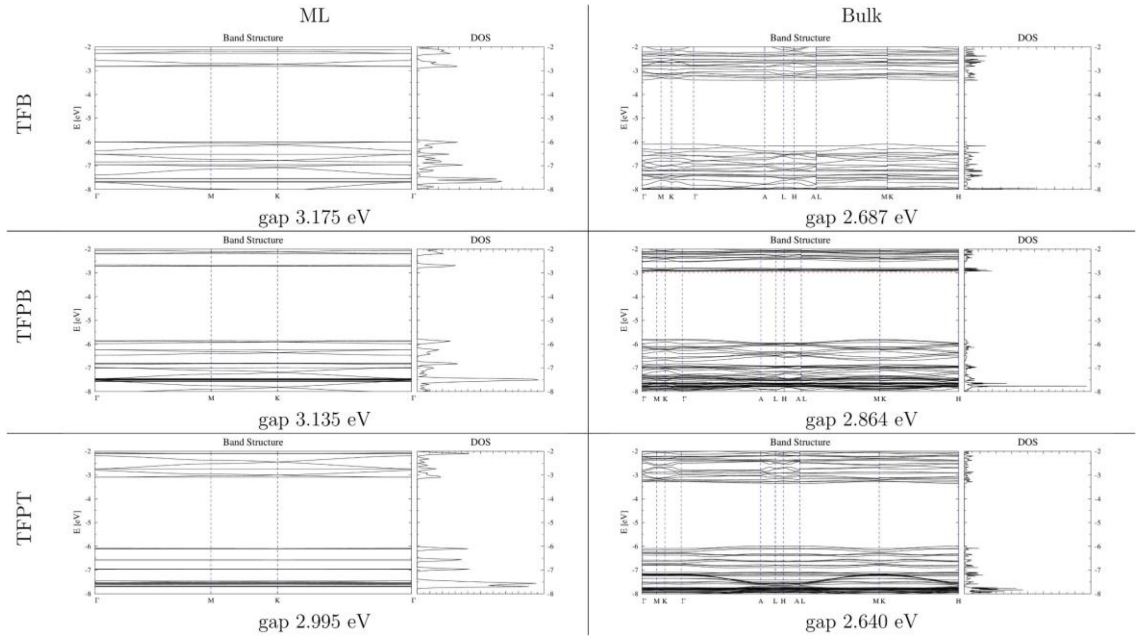


**Figure S52.** Band structure and corresponding Density of States (DOS) for TFB-Phen (top), TFPB-Phen (middle), and TFPT-Phen (bottom) monolayer (left) and bulk (right) COFs.

## 8. Characterization after ball milling

TFB-Phen showed remarkable mechanical stability, with all reflections retained in the PXRD pattern of TFB-Phen$_{BM}$ even after being subjected to harsh mechanical forces (Figure S53). FTIR analysis confirmed that the characteristic vibrational bands were conserved (Figure S54). Furthermore, nitrogen physisorption isotherm revealed <10% of loss in the SBET from 1064 to 969 m2 g$^{-1}$ after ball milling (Figure S55, S56), a small decrease in the $V_p$ from 0.58 to 0.50 cm$^3$ g$^{-1}$, while conserving the pore size distribution (Figure S57) of the pristine COF. TEM analysis revealed that TFB-Phen$_{BM}$ retained its ordered structure, while SEM analysis showed a slightly more granular morphology as compared to the as-synthesized material (Figures S58, S59). On the other hand, PXRD patterns of the ball-milled large-pore derivatives TFPB-Phen$_{BM}$ and TFPT-Phen$_{BM}$ revealed loss of structural order, in which the narrow diffraction peaks were replaced by broad reflections at $2\theta$ = 1.3 and 1.7°, respectively (Figures S60, S61), although FTIR analysis showed that both derivatives maintained their chemical connectivity (Figures S62, S63). SEM micrographs of the large-pore derivatives showed similar morphologies as the pristine materials (Figures S64, S65). The superior mechanical properties of the small-pore TFB-Phen further showcase the favorable intermolecular interactions within this COF.

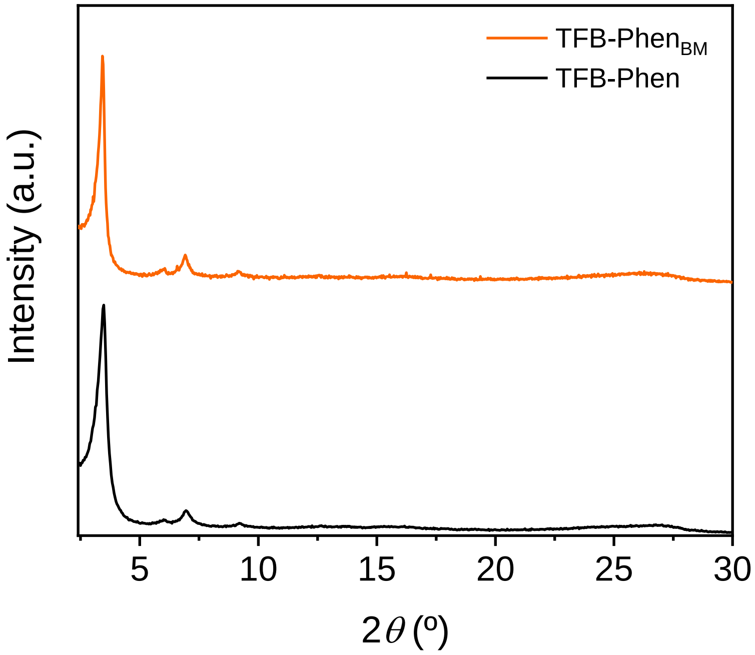


**Figure S53.** PXRD pattern of TFB-Phen and TFB-Phen$_{BM}$.

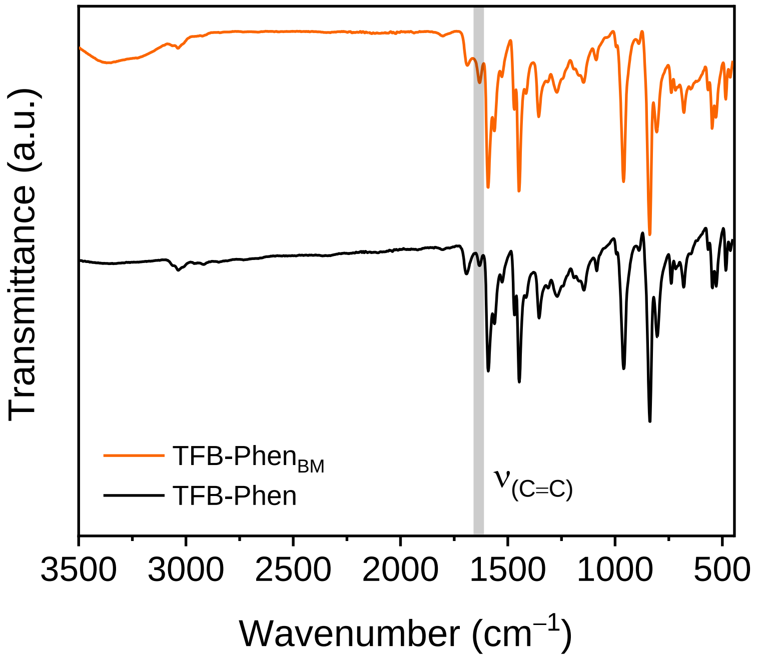


**Figure S54.** FT-IR spectra of TFB-Phen and TFB-Phen$_{BM}$.

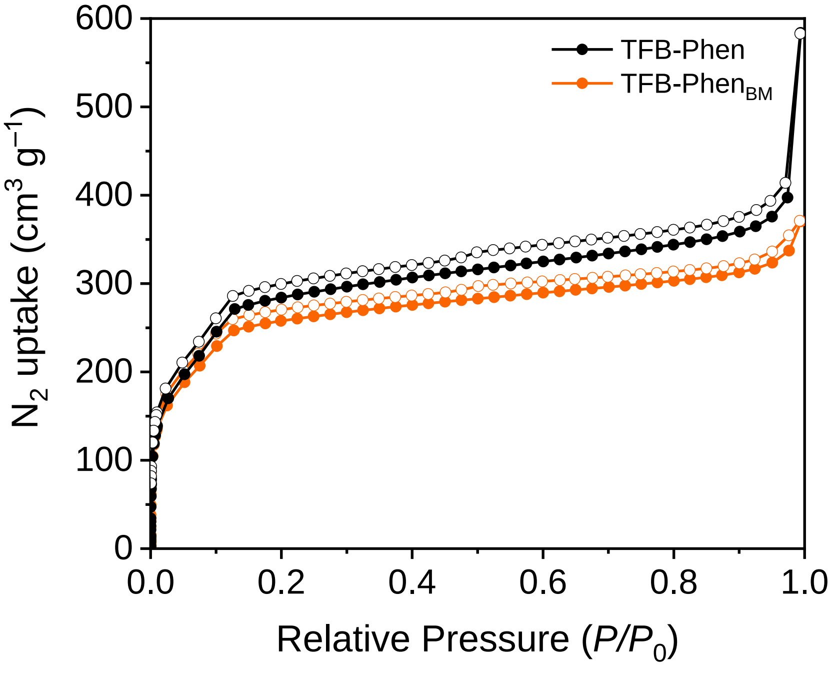


**Figure S55.** $N_2$ sorption isotherm profiles measured at 77 K of TFB-Phen and TFB-Phen$_{BM}$.

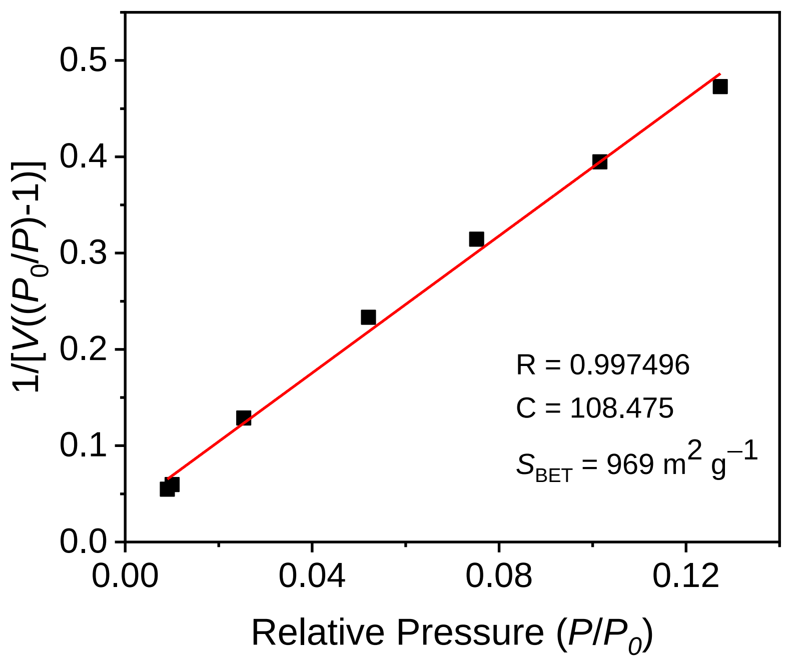


**Figure S56.** Multi-point BET plot and linear fit of TFB-Phen$_{BM}$. Using BETSI software $S_{BET}$ = 946 $m^2$ $g^{-1}$ ($R^2$ = 0.995024, C = 153.421).

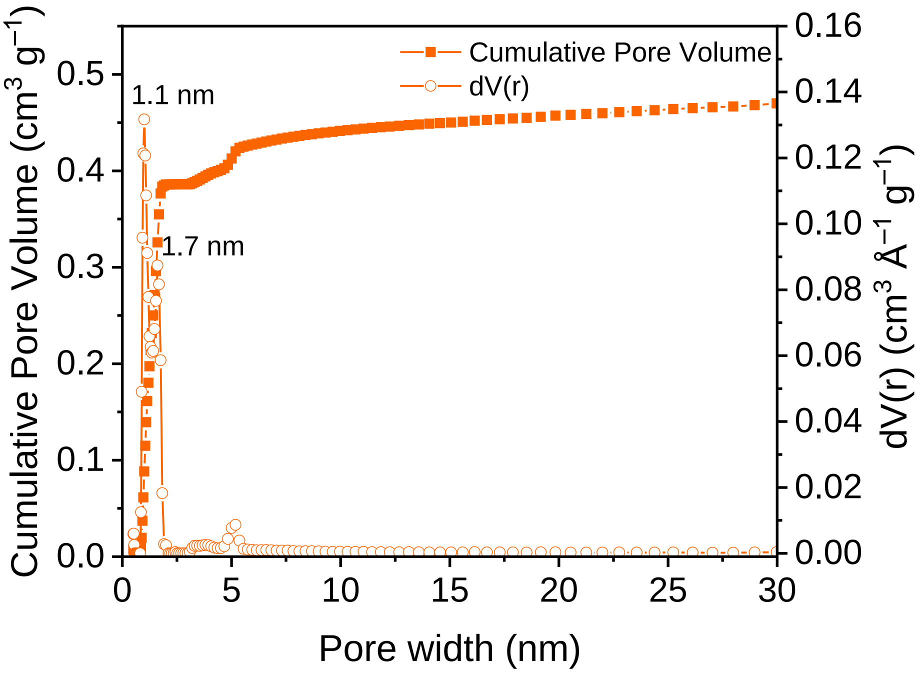


**Figure S57.** Pore size distribution (hollow spheres) and cumulative pore volume (filled spheres) profile of TFB-Phen$_{BM}$. QSDFT model for slit/cylindrical pores, fitting error of 0.384%.

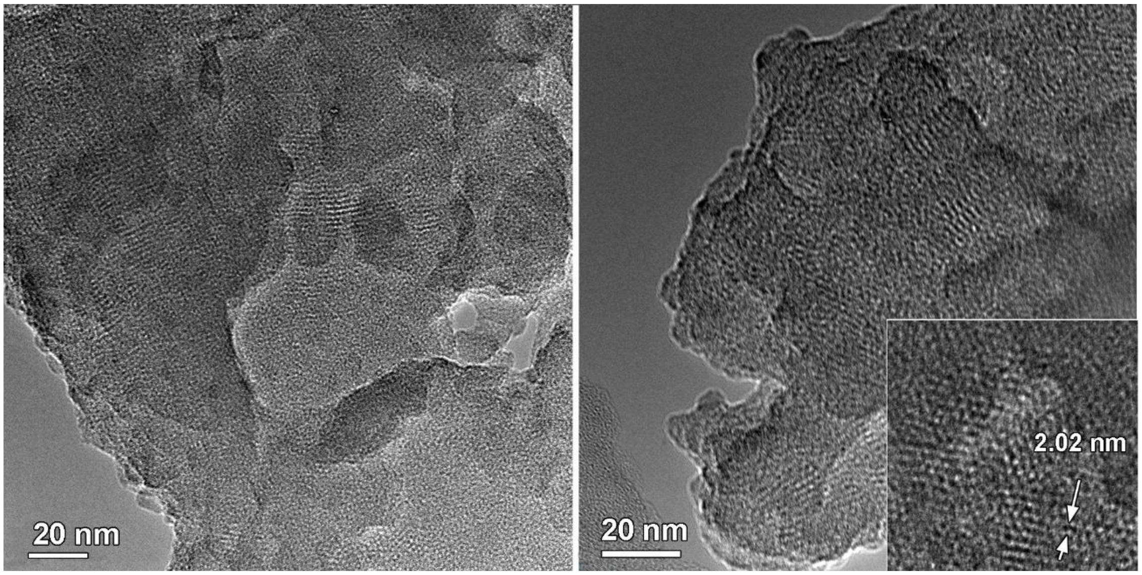


**Figure S58.** Bright field HR-TEM micrographs of different pieces of TFB-Phen$_{BM}$.

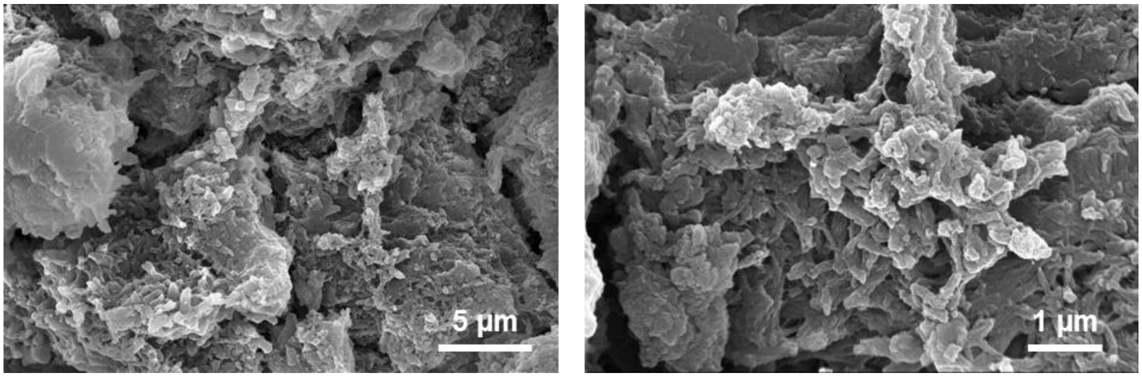


**Figure S59.** SEM micrographs of TFB-Phen$_{BM}$.

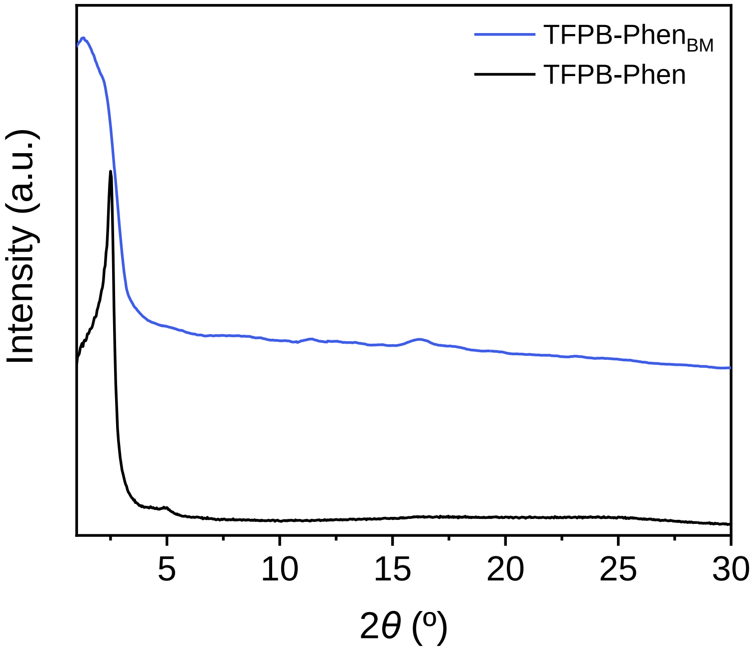


**Figure S60.** PXRD pattern of TFPB-Phen and TFPB-Phen$_{BM}$.

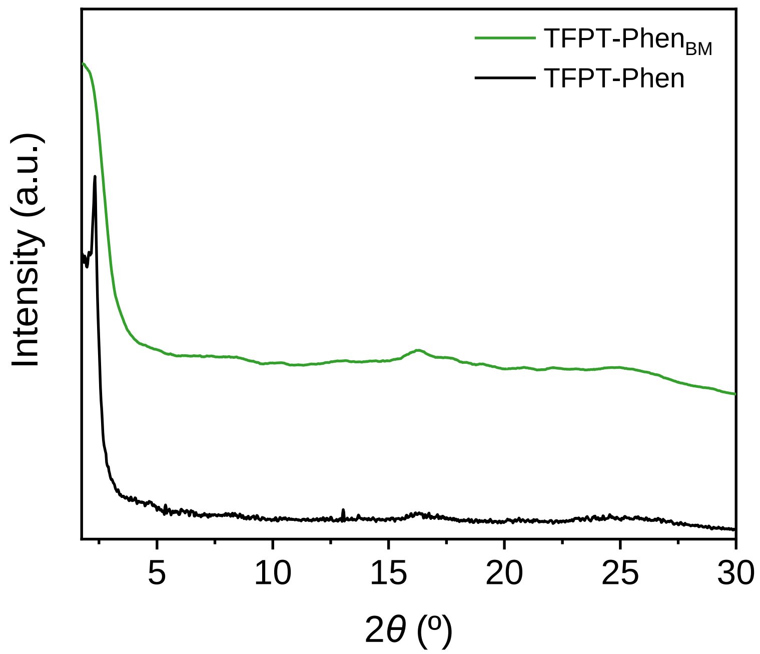


**Figure S61.** PXRD pattern of TFPT-Phen and TFPT-Phen$_{BM}$.

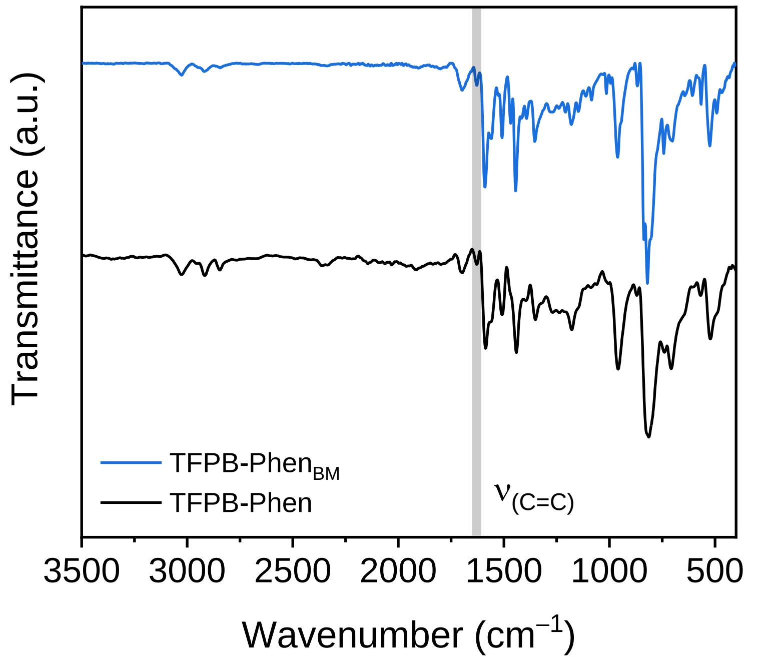


**Figure S62.** FT-IR spectra of TFPB-Phen and TFPB-Phen$_{BM}$.

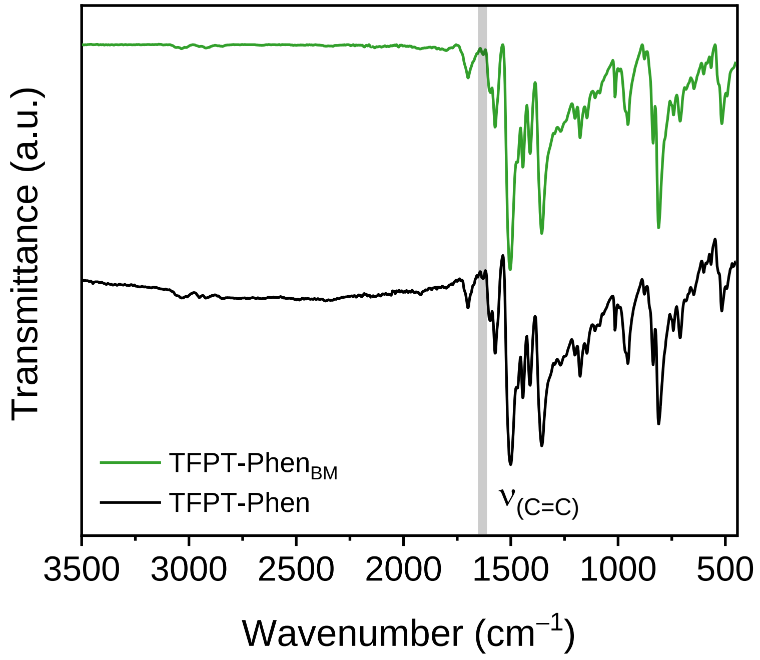


**Figure S63.** FT-IR spectra of TFPT-Phen and TFPT-Phen$_{BM}$.

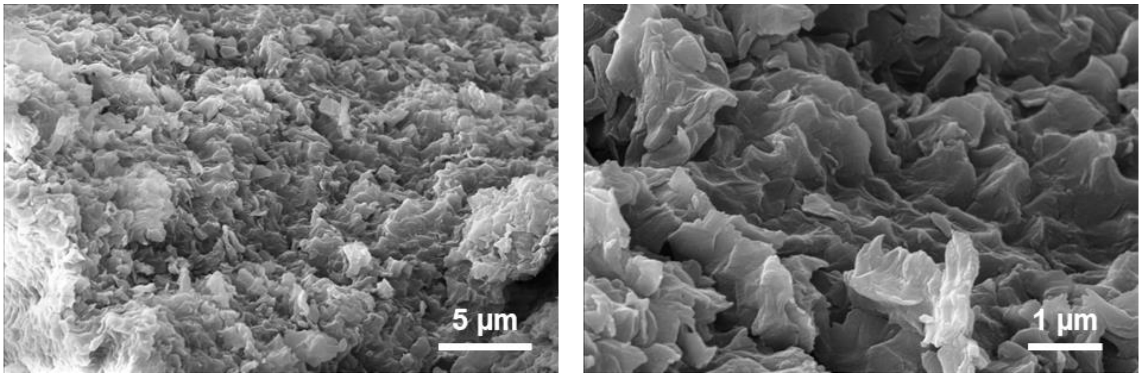


**Figure S64.** SEM micrographs of TFPB-Phen$_{BM}$.

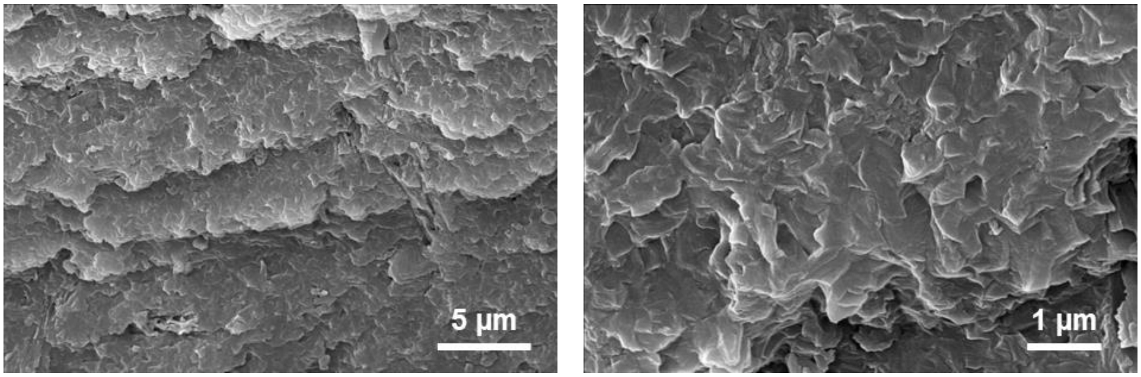


**Figure S65.** SEM micrographs of TFPT-Phen$_{BM}$.

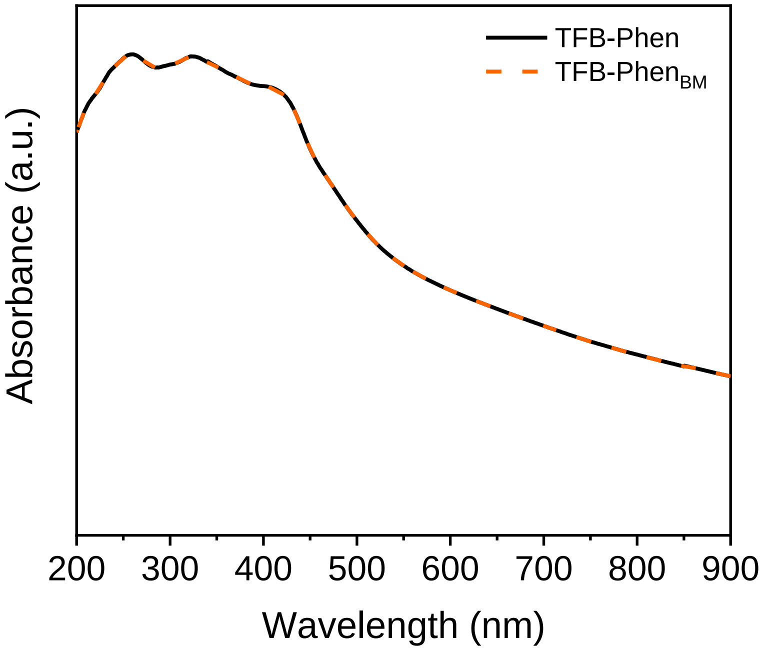


**Figure S66.** UV-vis DR spectra of TFB-Phen and TFB-Phen$_{BM}$.

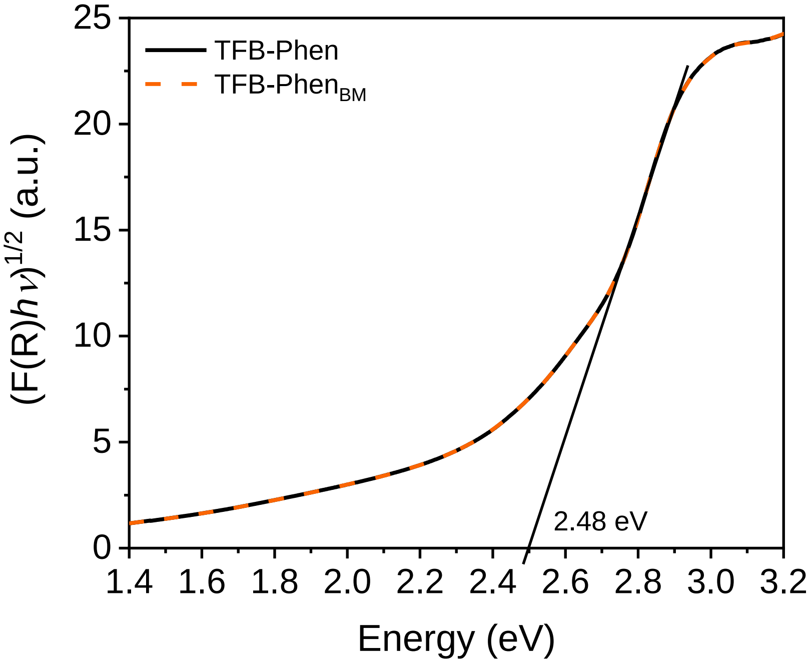


**Figure S67.** Band gap energy of TFB-Phen determined from the K–M transformed reflectance spectra before and after ball milling.

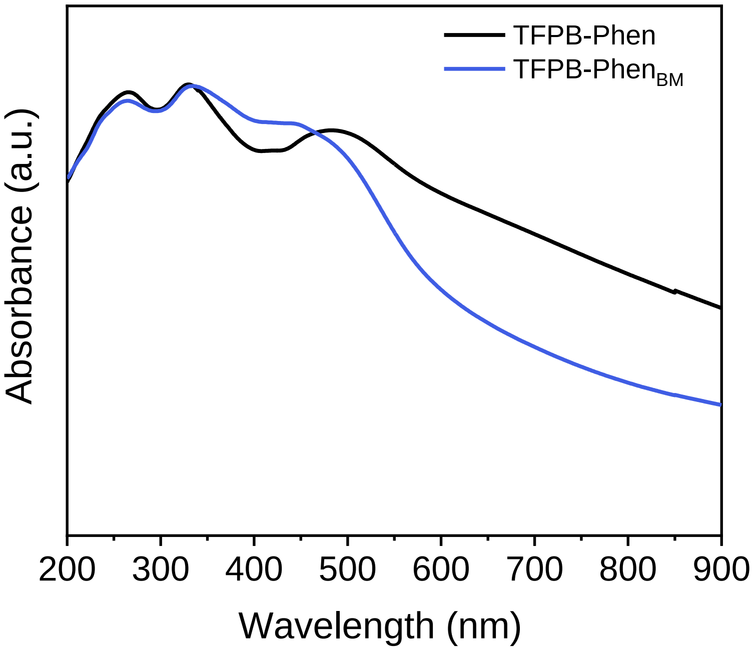


**Figure S68.** UV-vis DR spectra of TFPB-Phen and TFPB-Phen$_{BM}$.

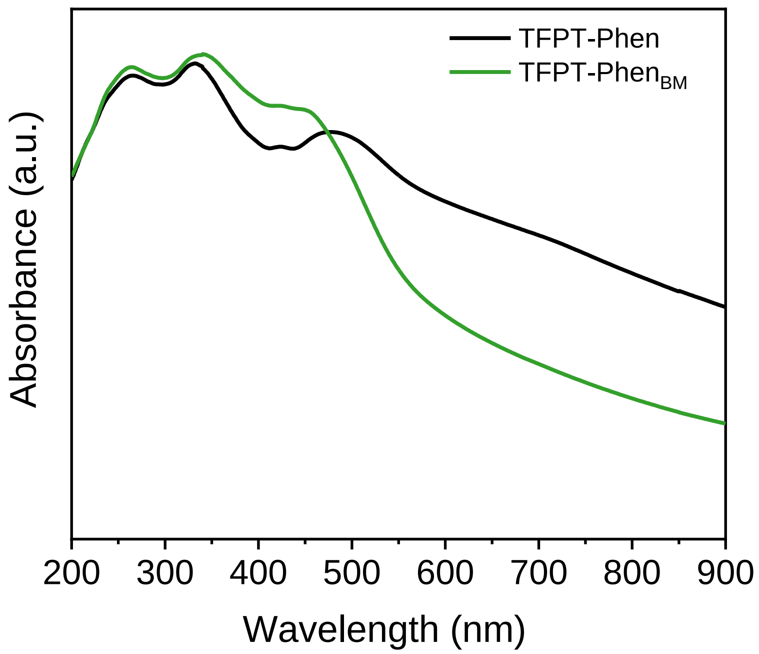


**Figure S69.** UV-vis DR spectra of TFPT-Phen and TFPT-Phen$_{BM}$.

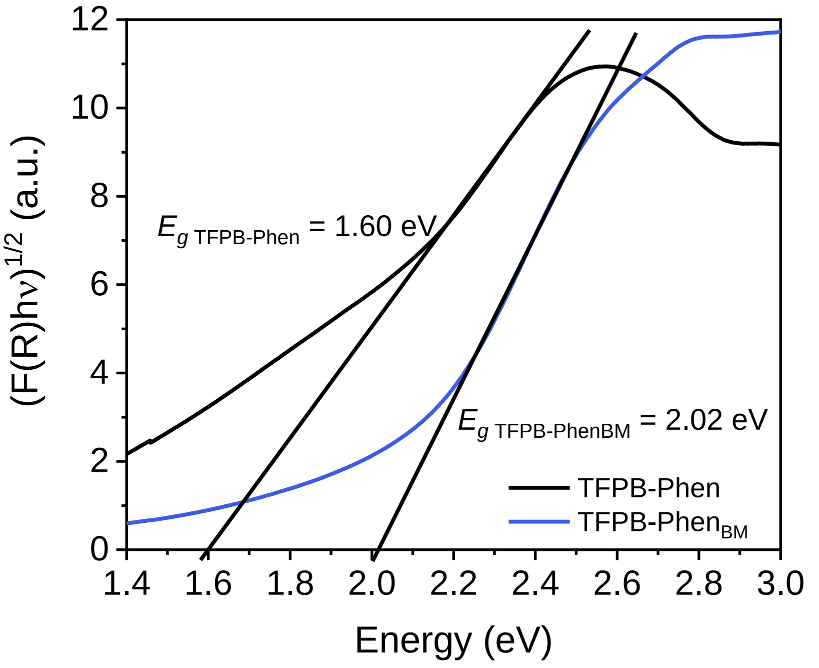


**Figure S70.** Band gap energy of TFPB-Phen determined from the K–M transformed reflectance spectra before and after ball milling.

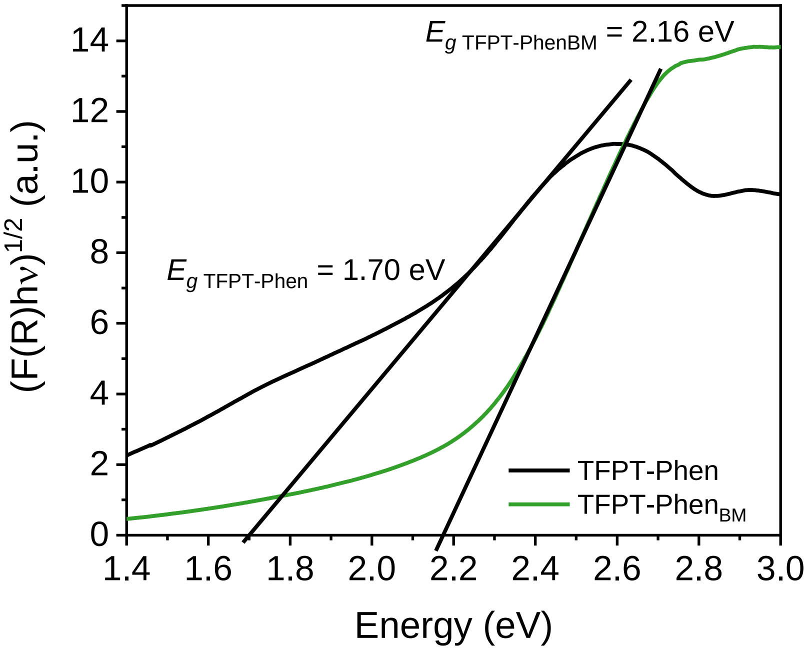


**Figure S71.** Band gap energy of TFPT-Phen determined from the K–M transformed reflectance spectra before and after ball milling.

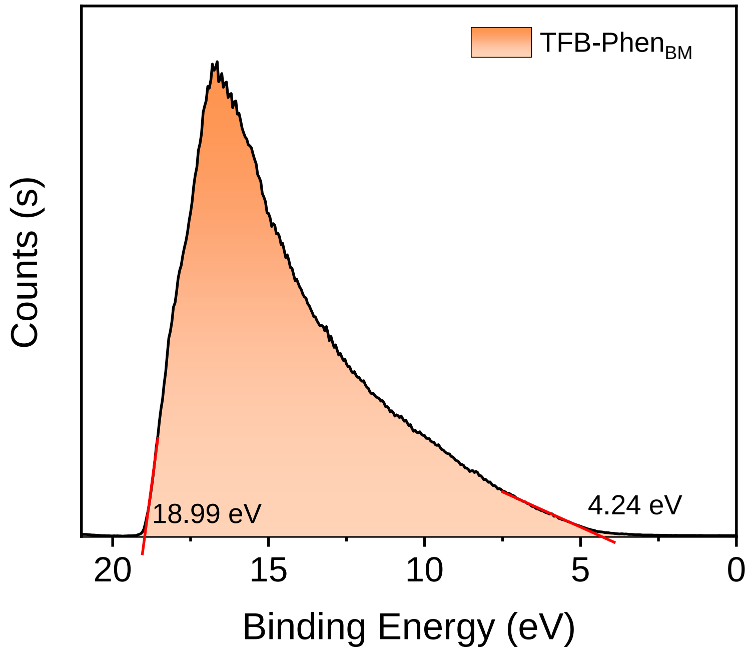


**Figure S72.** UPS spectrum of TFB-Phen$_{BM}$.

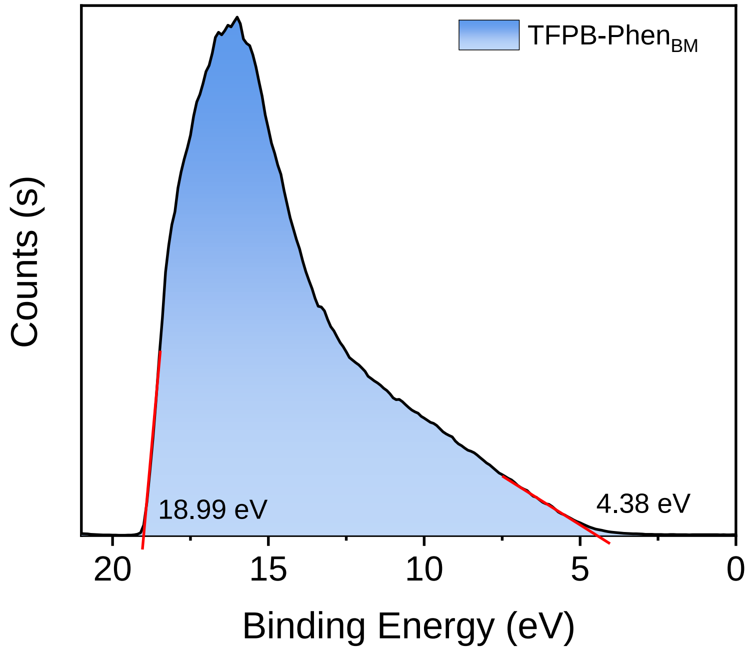


**Figure S73.** UPS spectrum of TFPB-Phen$_{BM}$.

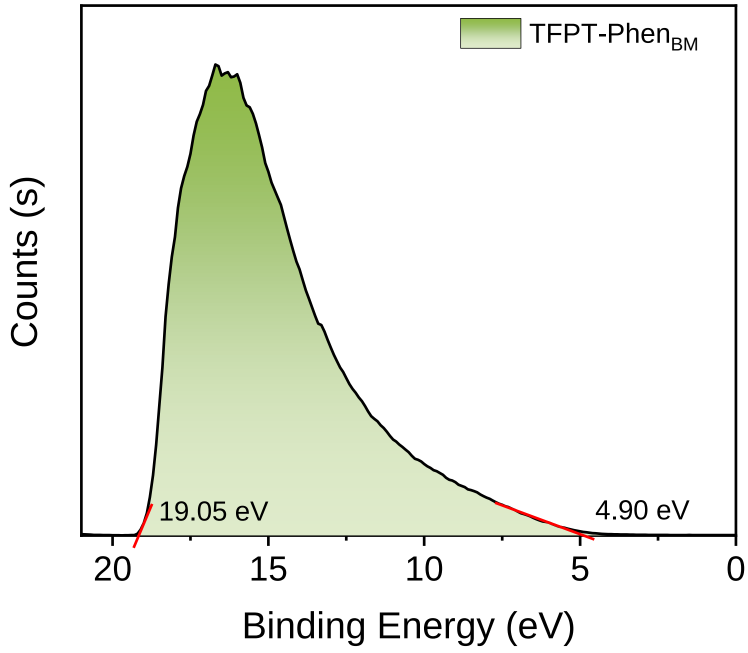


**Figure S74.** UPS spectrum of TFPT-Phen$_{BM}$.

# 9. Photodegradation of dyes

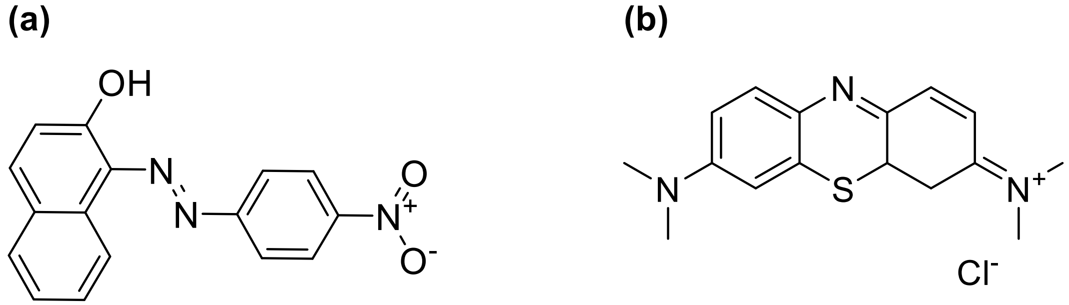


**Figure S75.** Chemical structure of (a) Para Red (PR) and (b) Methylene Blue (MB).

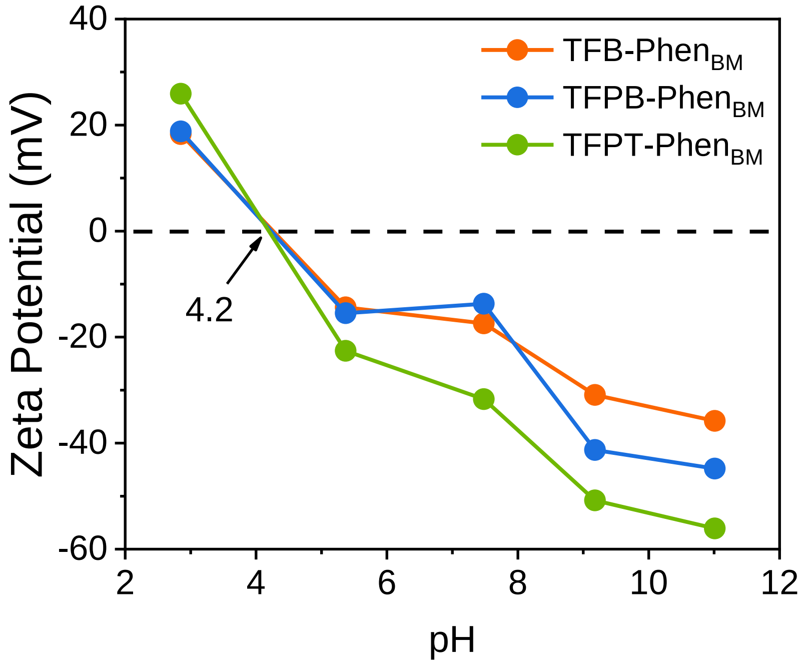


**Figure S76.** The isoelectric point measurement for TFB-Phen$_{BM}$, TFPB-Phen$_{BM}$, and TFPT-Phen$_{BM}$.

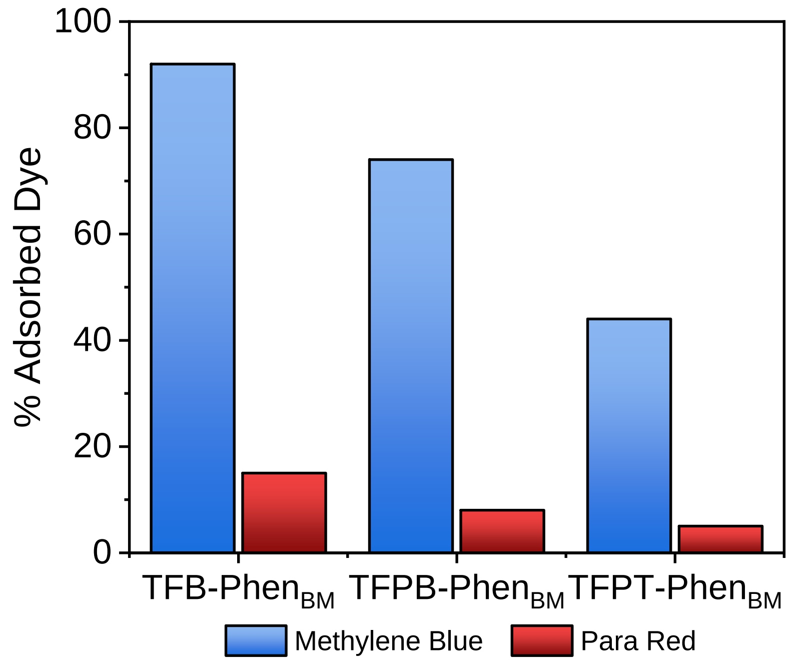


**Figure S77.** Comparison of adsorption efficiencies of MB and PR by TFB-Phen$_{BM}$, TFPB-Phen$_{BM}$, and TFPT-Phen$_{BM}$ materials. ([MB] = 10 ppm in water t = 2 h) and PR ([PR] = 10 ppm in $CH_3CN/H_2O$ 4:1, t = 4.5 h). Dosage = 0.8 mg/mL.

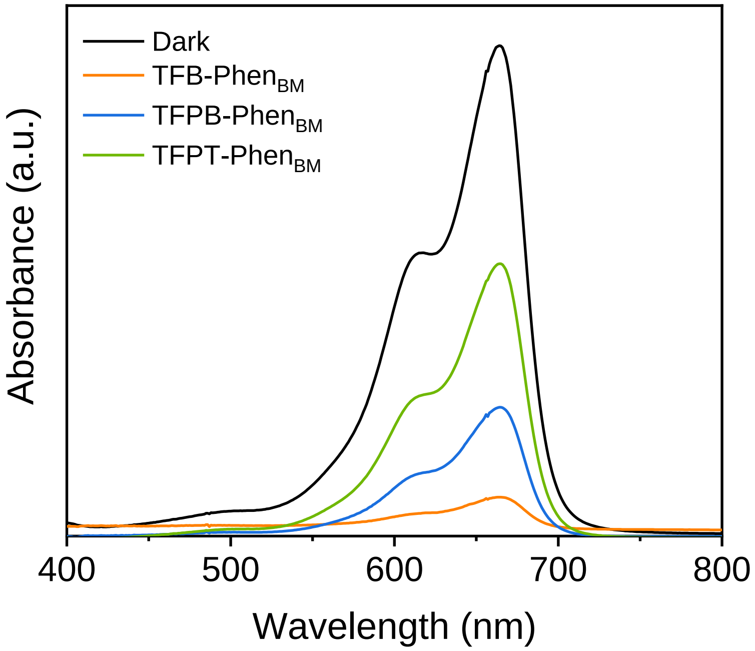


**Figure S78.** Adsorption of MB by TFB-Phen$_{BM}$, TFPB-Phen$_{BM}$, and TFPT-Phen$_{BM}$ materials.

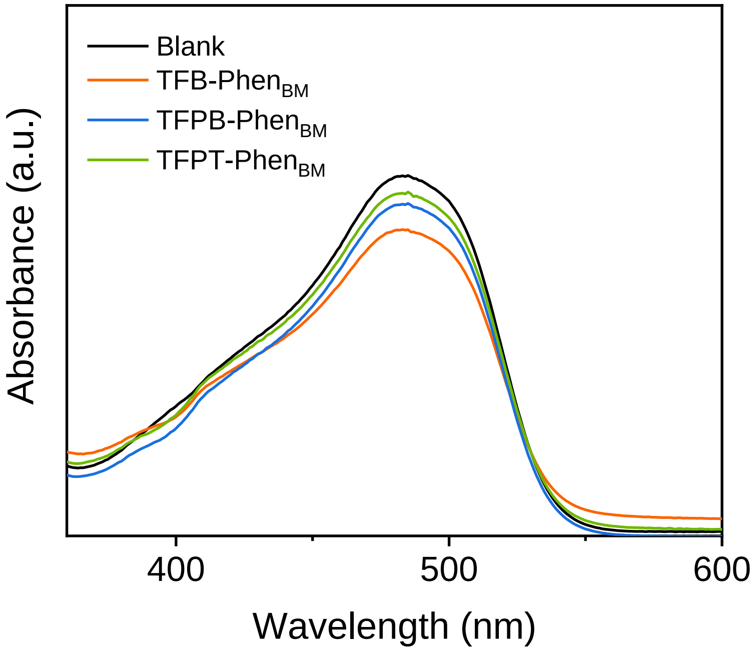


**Figure S79.** Adsorption of PR by TFB-Phen$_{BM}$, TFPB-Phen$_{BM}$, and TFPT-Phen$_{BM}$ materials.

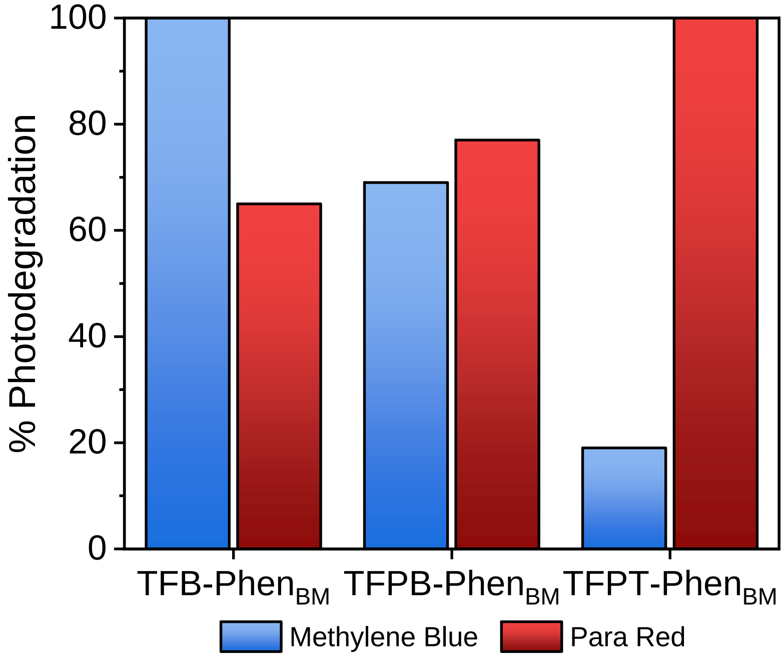


**Figure S80.** Comparison of photodegradation efficiencies of MB and PR by TFB-Phen$_{BM}$, TFPB-Phen$_{BM}$, and TFPT-Phen$_{BM}$ materials. ([MB] = 10 ppm in water t = 2 h) and PR ([PR] = 10 ppm in $CH_3CN/H_2O$ 4:1, t = 4.5 h). Dosage = 0.8 mg/mL.

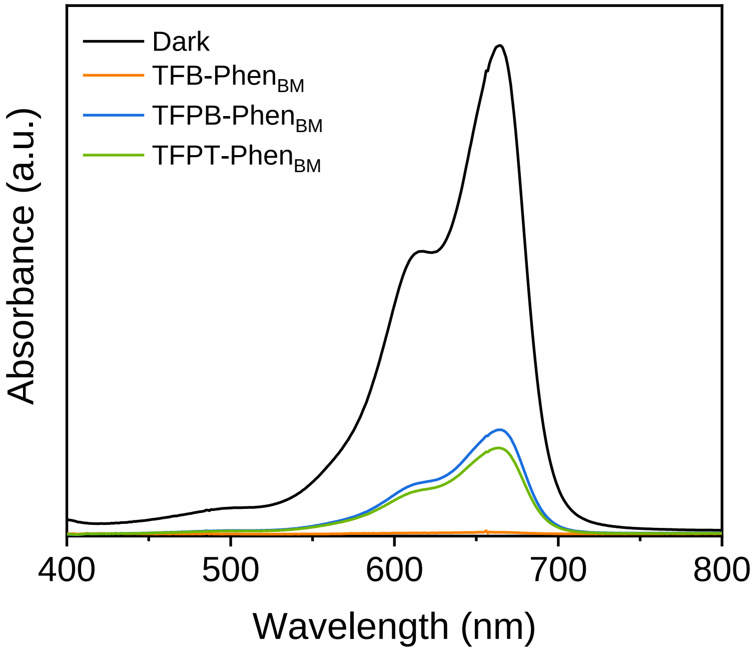


**Figure S81.** Photodegradation of MB by TFB-Phen$_{BM}$, TFPB-Phen$_{BM}$, and TFPT-Phen$_{BM}$ materials.

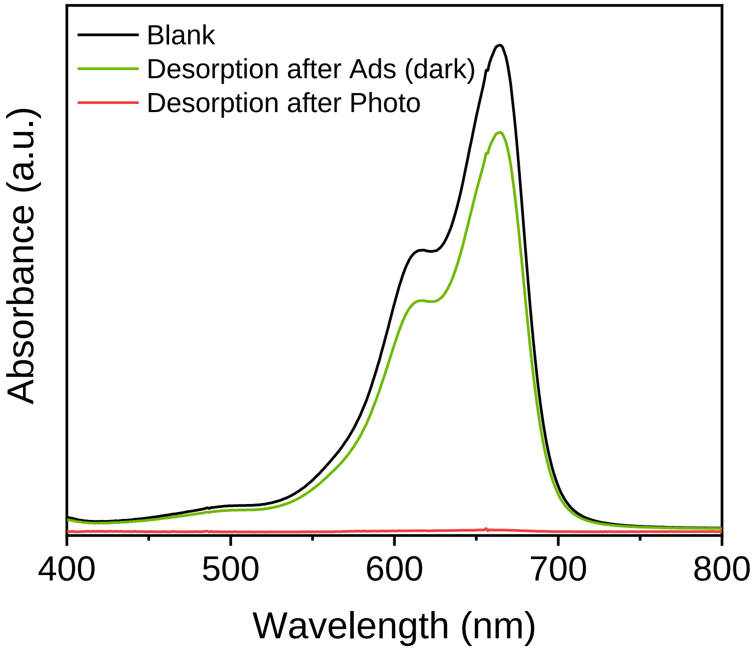


**Figure S82.** Desorption experiments of MB by TFB-Phen$_{BM}$ material after adsorption (Ads) and after photocatalysis (Photo).

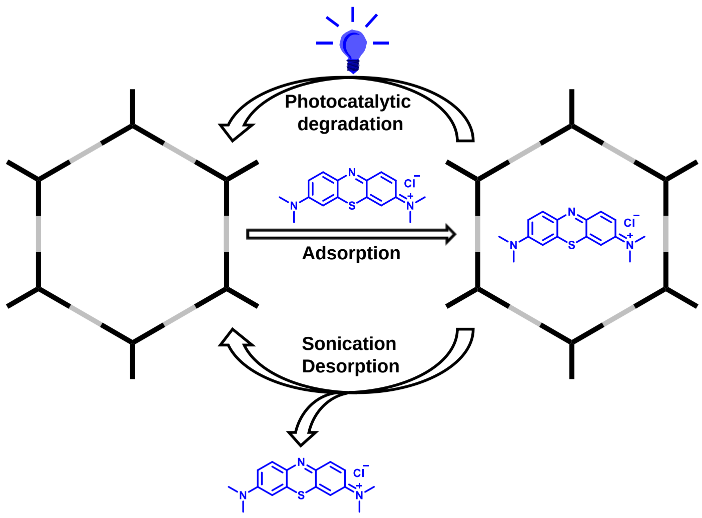


**Figure S83.** Schematic representation of adsorption, desorption, and photodegradation of MB mediated by TFB-Phen$_{BM}$.

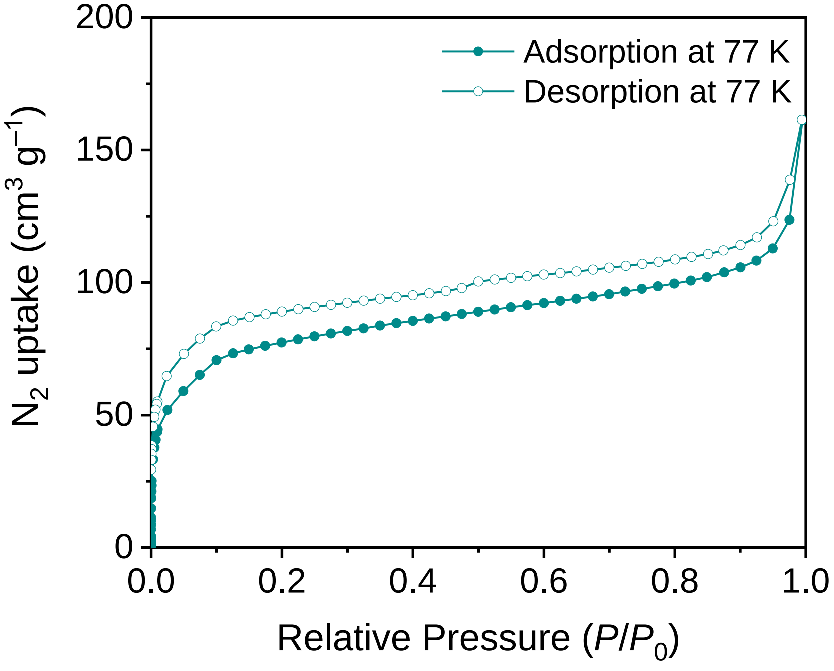


**Figure S84.** $N_2$ sorption isotherm profiles measured at 77 K of TFB-Phen$_{BM1\ h}$.

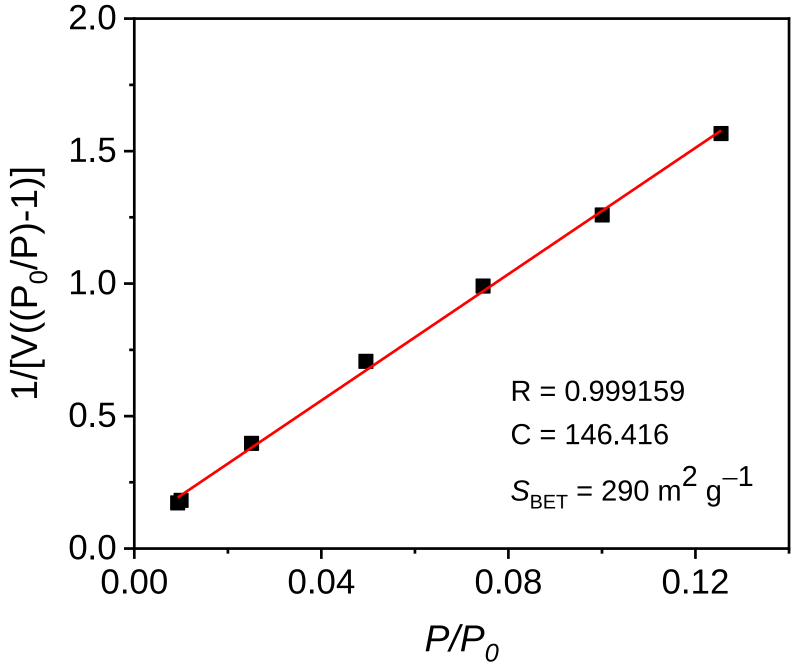


**Figure S85.** Multi-point BET plot and linear fit of TFB-Phen$_{BM1\ h}$. Using BETSI software $S_{BET}$ = 286 $m^2$ $g^{-1}$ ($R^2$ = 0.998189, C = 192.294).

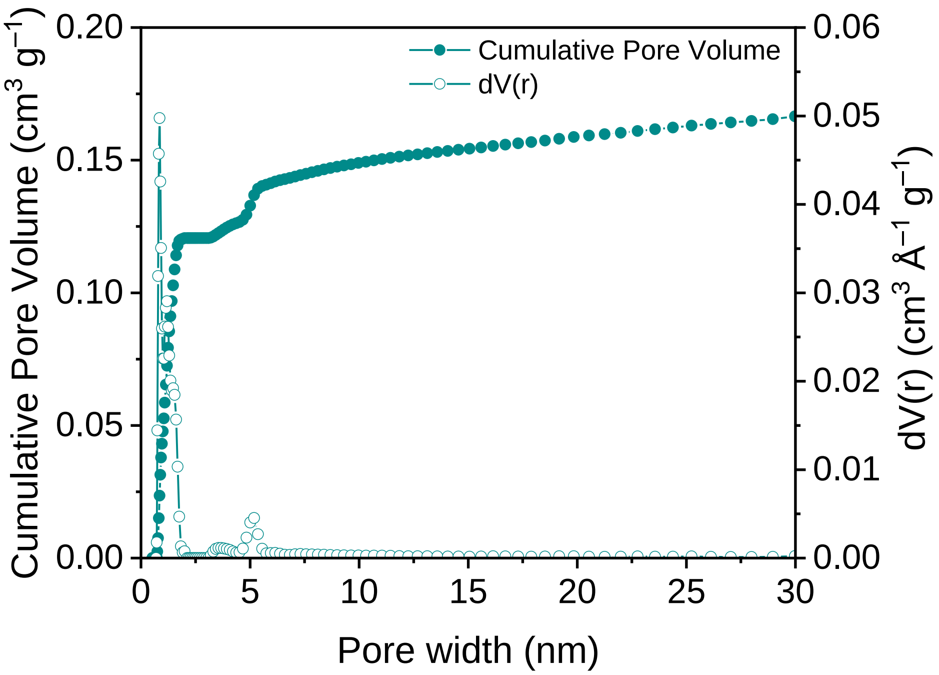


**Figure S86.** Pore size distribution (hollow spheres) and cumulative pore volume (filled spheres) profile of TFB-Phen$_{BM1\ h}$. QSDFT model for slit/cylindrical pores, fitting error of 0.749%.

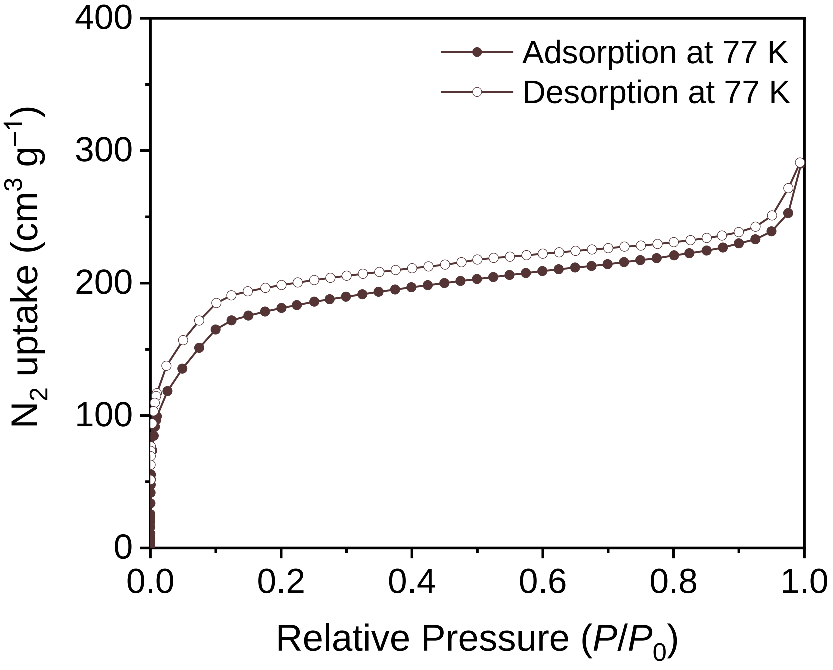


**Figure S87.** $N_2$ sorption isotherm profiles measured at 77 K of TFB-Phen$_{BM6\ h}$.

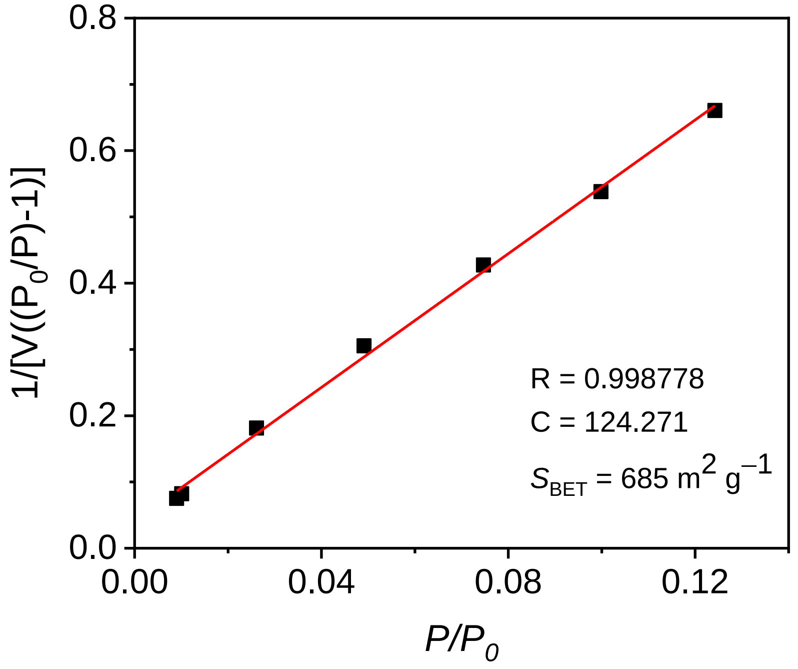


**Figure S88.** Multi-point BET plot and linear fit of TFB-Phen$_{BM6\ h}$. Using BETSI software $S_{BET}$ = 671 m$^2$ g$^{-1}$ ($R^2$ = 0.997270, C = 168.389).

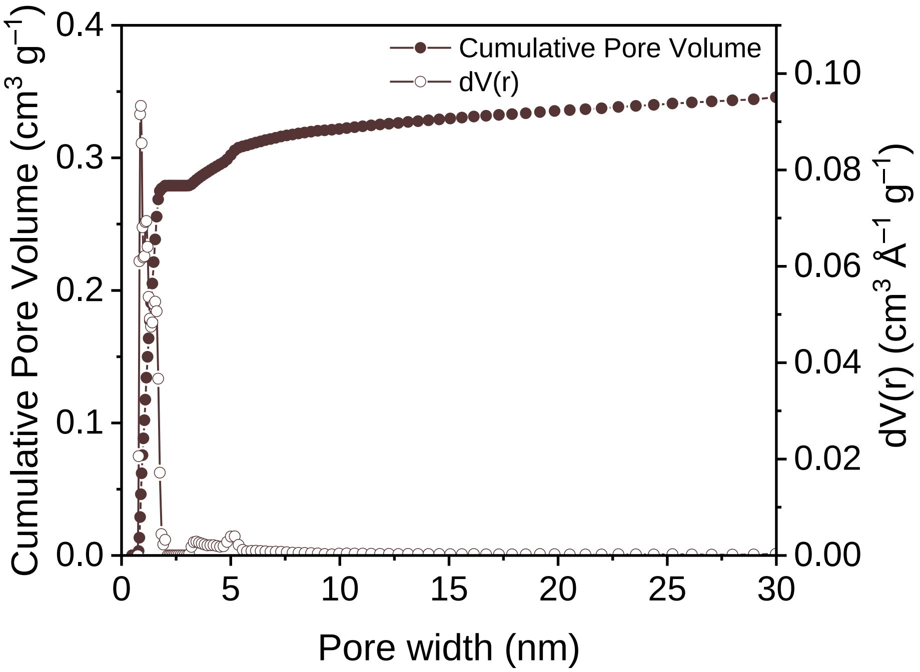


**Figure S89.** Pore size distribution (hollow spheres) and cumulative pore volume (filled spheres) profile of TFB-Phen$_{BM6\ h}$. QSDFT model for slit/cylindrical pores, fitting error of 0.483%.

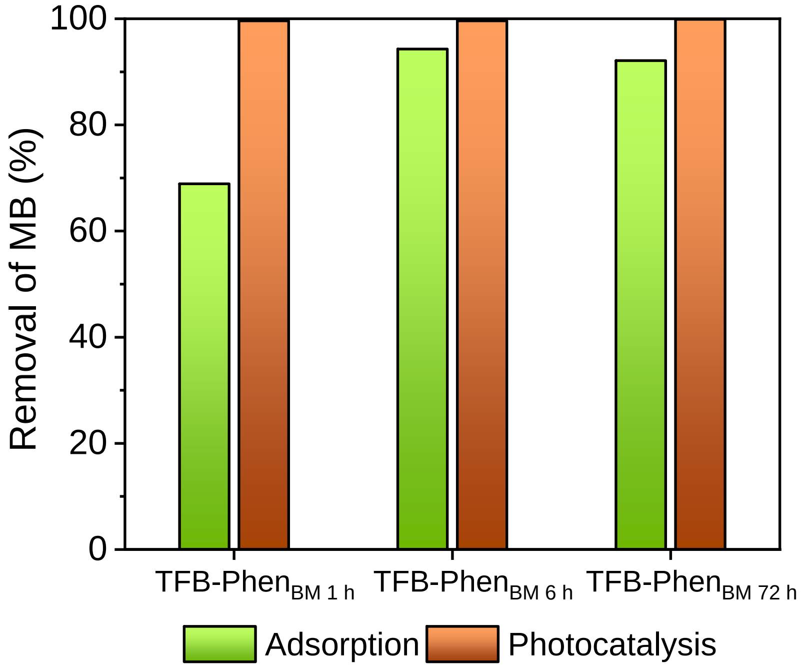


**Figure S90.** Removal efficiency of MB through adsorption and photocatalysis by TFB-Phen$_{BM}$ synthesized at different times with distinct $S_{BET}$ (1 h = 290 m$^2$ g$^{-1}$; 6 h = 685 m$^2$ g$^{-1}$; 72 h = 969 m$^2$ g$^{-1}$).

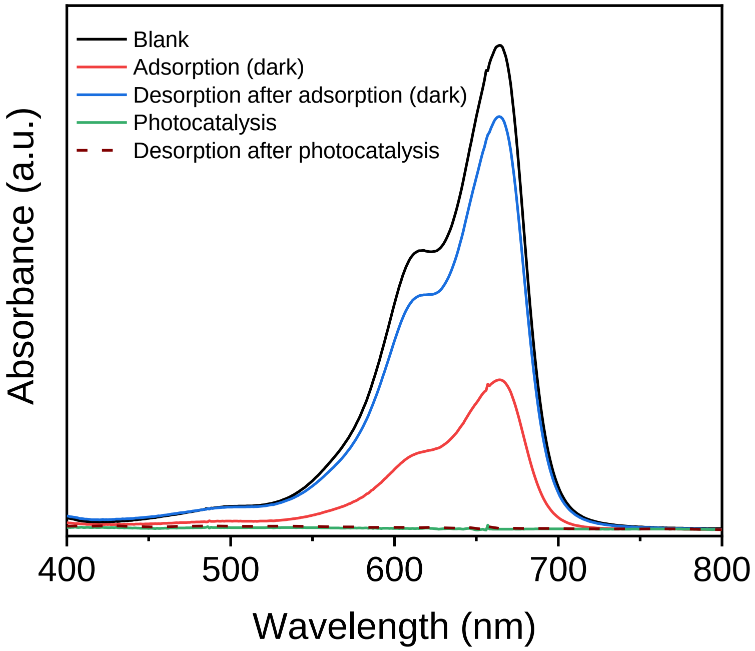


**Figure S91.** Adsorption and photocatalysis and respective desorption experiments of MB by TFB-Phen$_{\mathrm{BM1\ h}}$.

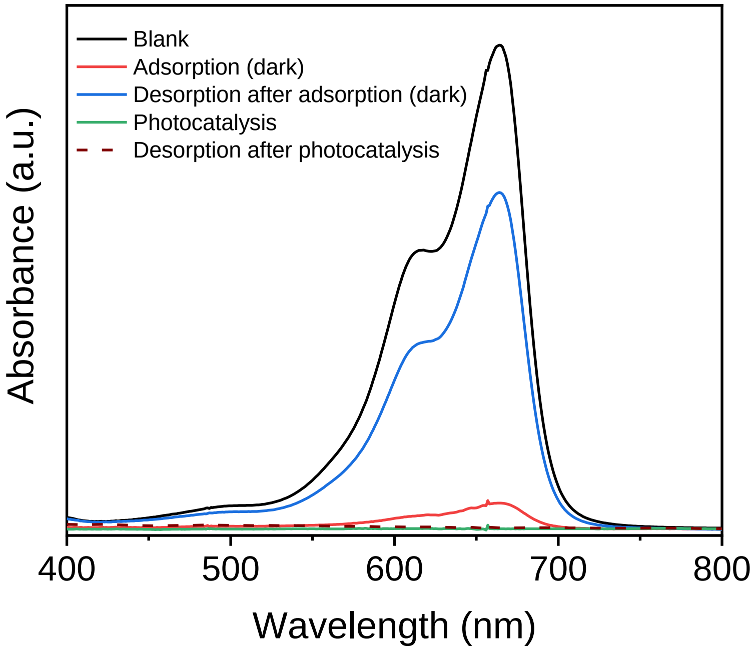


**Figure S92.** Adsorption and photocatalysis and respective desorption experiments of MB by TFB-Phen$_{\mathrm{BM6\ h}}$.

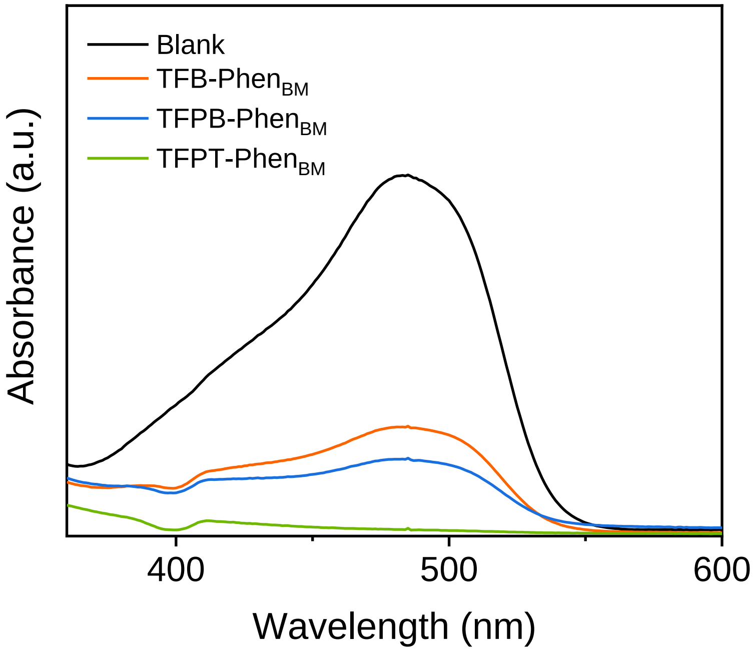


**Figure S93.** Photodegradation of PR by TFB-Phen$_{BM}$, TFPB-Phen$_{BM}$, and TFPT-Phen$_{BM}$ materials.

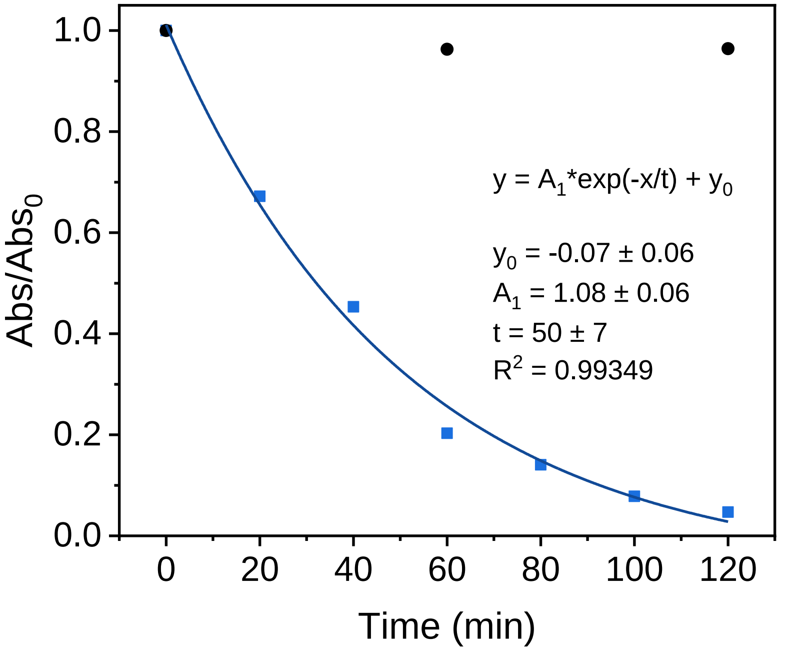


**Figure S94.** Kinetic experiment of photodegradation of MB by TFB-Phen$_{BM}$ (blue) and respective control under irradiation and in the absence of the catalyst (black).

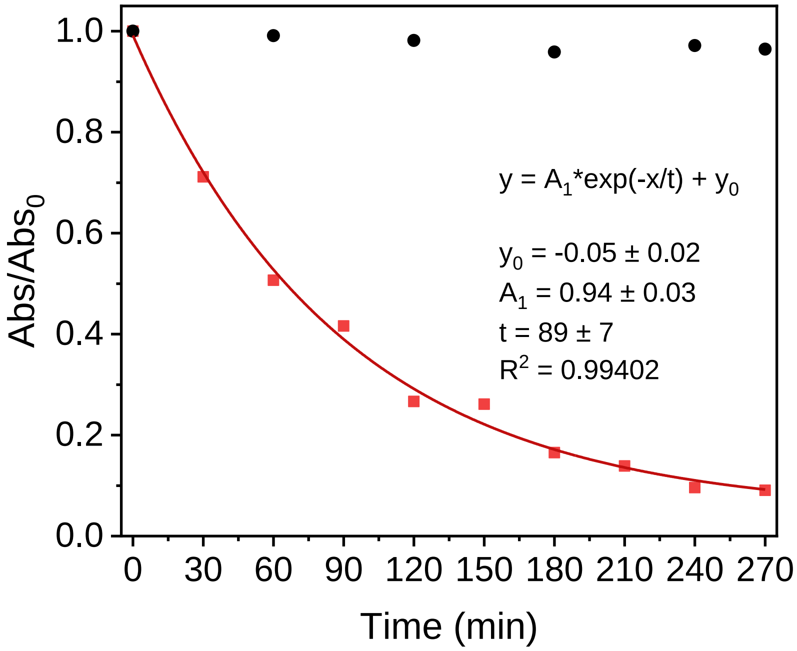


**Figure S95.** Kinetic experiment of photodegradation of PR by TFPT-Phen$_{BM}$ (red) and respective control under irradiation and in the absence of the catalyst (black).

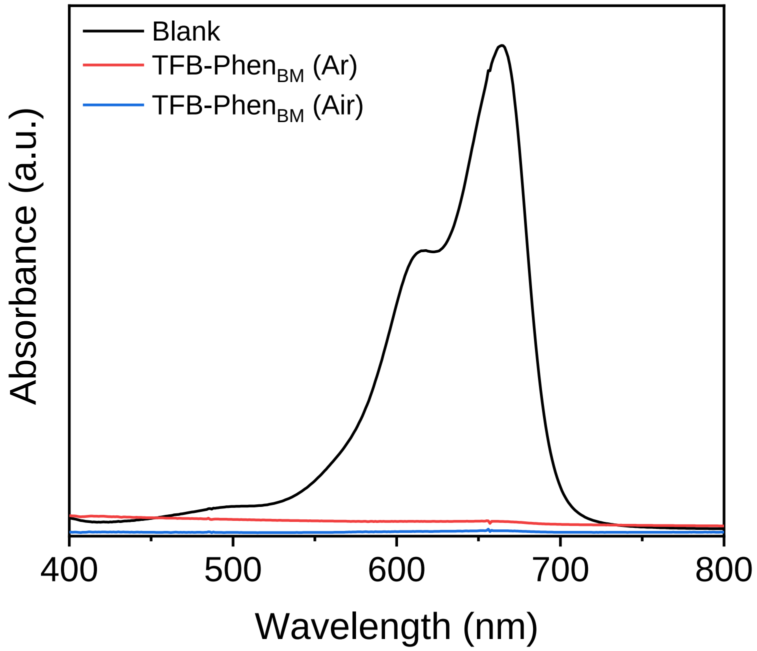


**Figure S96.** Irradiation experiments of MB by TFB-Phen$_{BM}$ in presence or absence of oxygen.

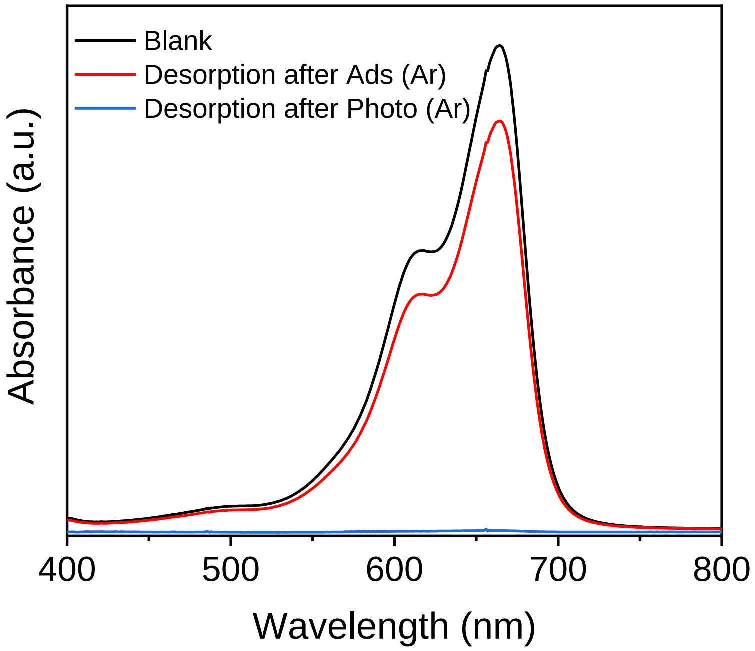


**Figure S97.** Desorption experiments of MB by TFB-Phen$_{BM}$ material in Ar atmosphere after adsorption (Ads) and after photocatalysis (Photo).

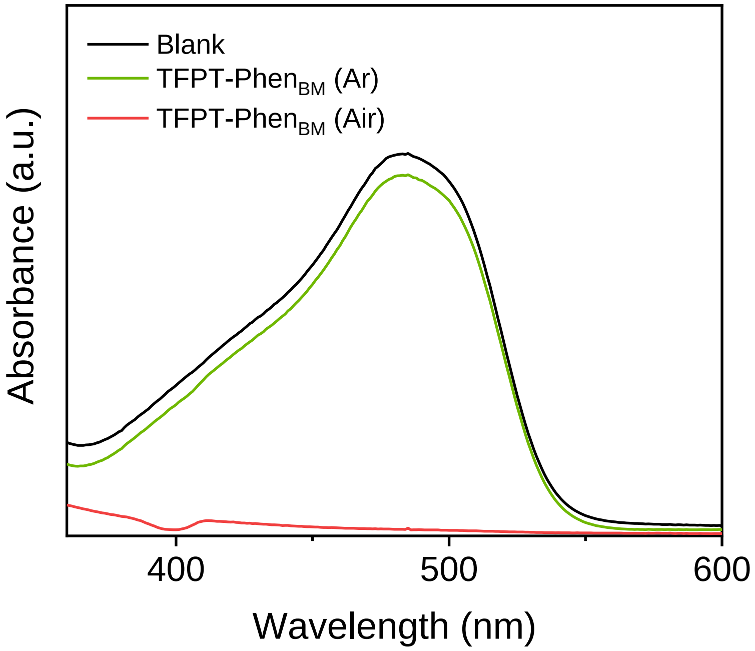


**Figure S98.** Irradiation experiments of PR mediated by TFPT-Phen$_{BM}$ in presence or absence of oxygen.

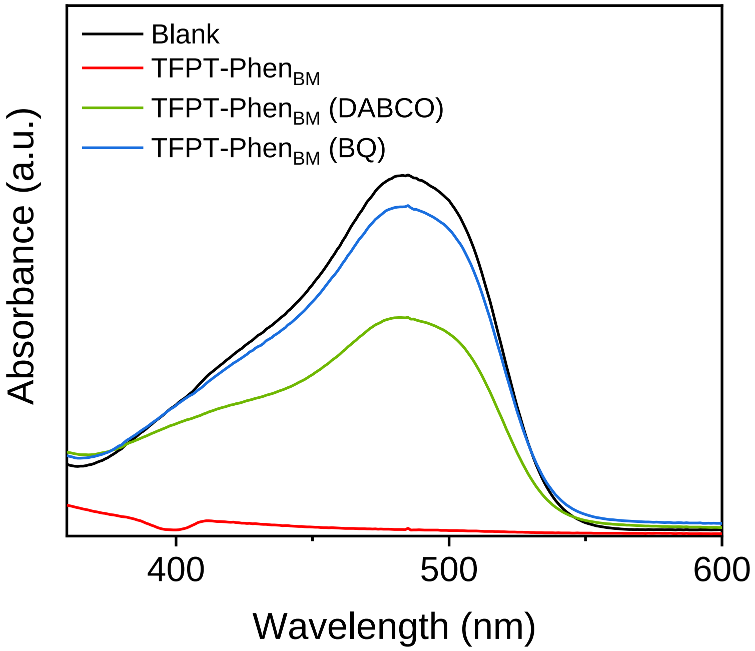


**Figure S99.** Irradiation experiments of PR mediated by TFPT-Phen$_{BM}$ material in presence or absence of selective quenchers (DABCO, singlet oxygen quencher; BQ, superoxide radical anion quencher).

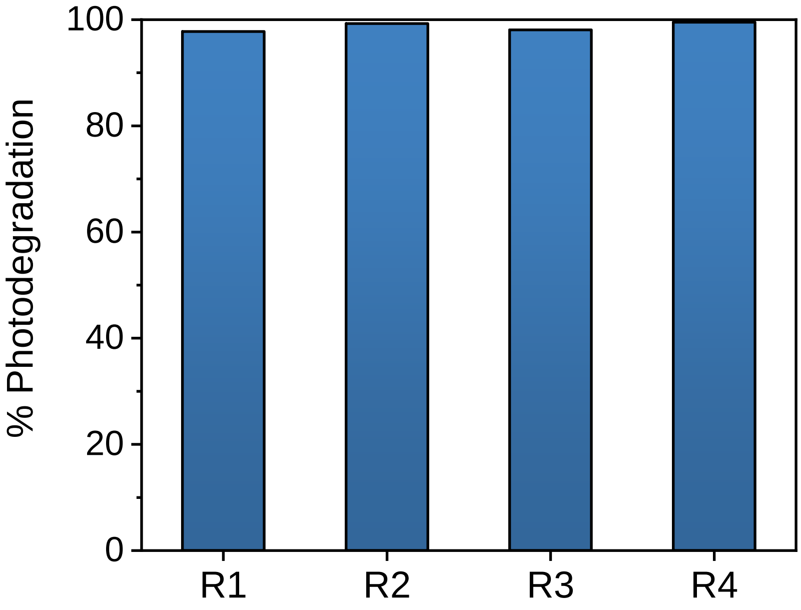


**Figure S100.** Recyclability experiments of TFB-Phen$_{BM}$ for MB photodegradation.

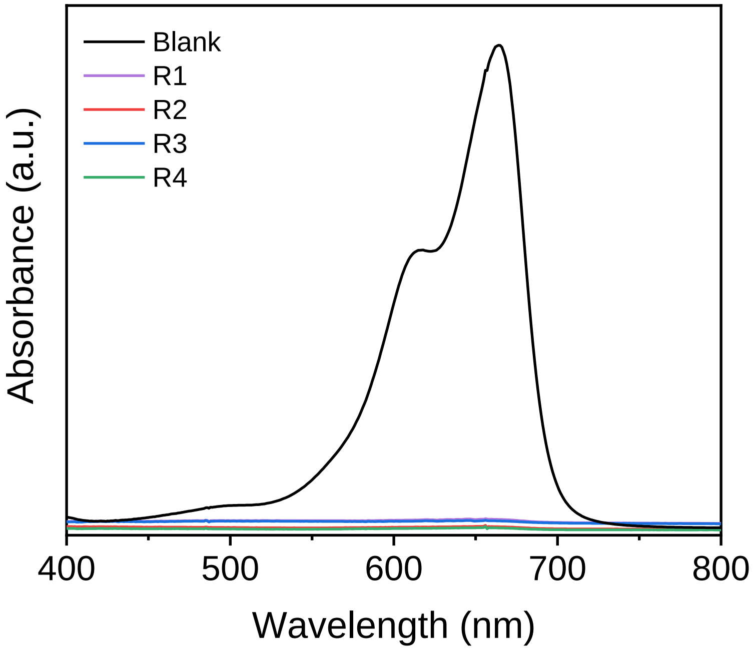


**Figure S101.** UV-vis spectra of the recyclability experiments of TFB-Phen$_{BM}$ for MB photodegradation.

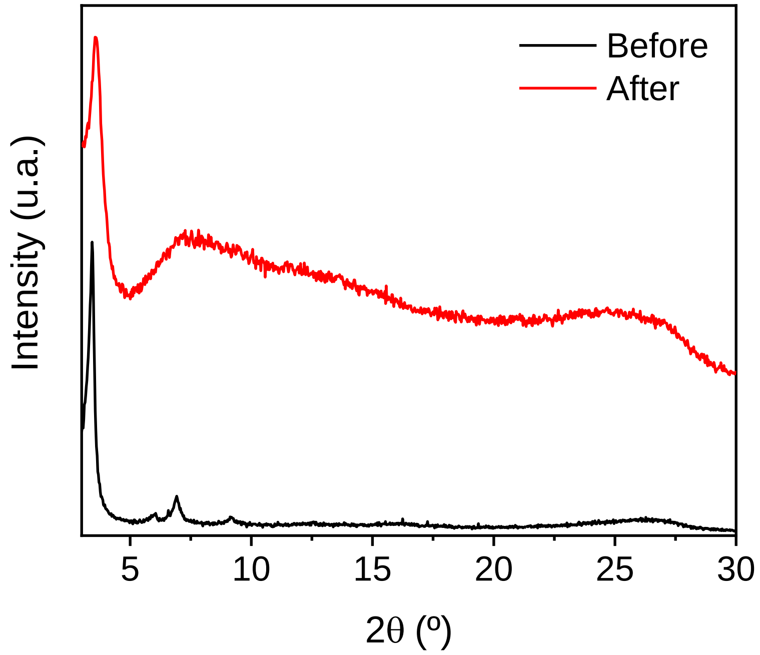


**Figure S102.** PXRD pattern of TFB-Phen$_{BM}$ before and after photocatalysis. The PXRD analysis after photocatalysis, performed by only drying the sample without further desorption and washing treatments, confirmed that crystallinity was largely maintained. The observed peak broadening may result from unwashed substances attached to the COF surface or within its pores. Additionally, since water was not replaced with a lower-boiling-point solvent, residual water molecules likely remain, also influencing the PXRD results.

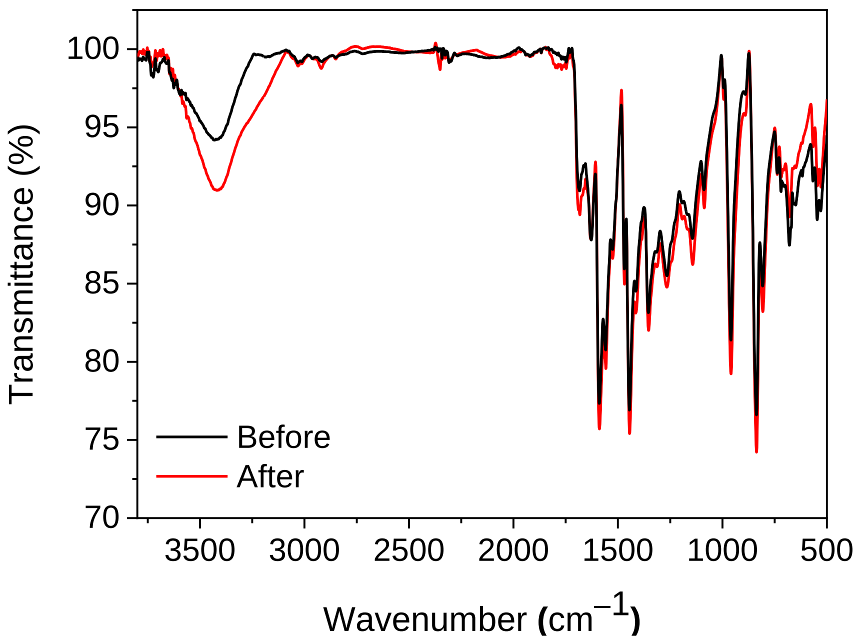


**Figure S103.** FT-IR of TFB-Phen$_{BM}$ before and after photocatalysis.

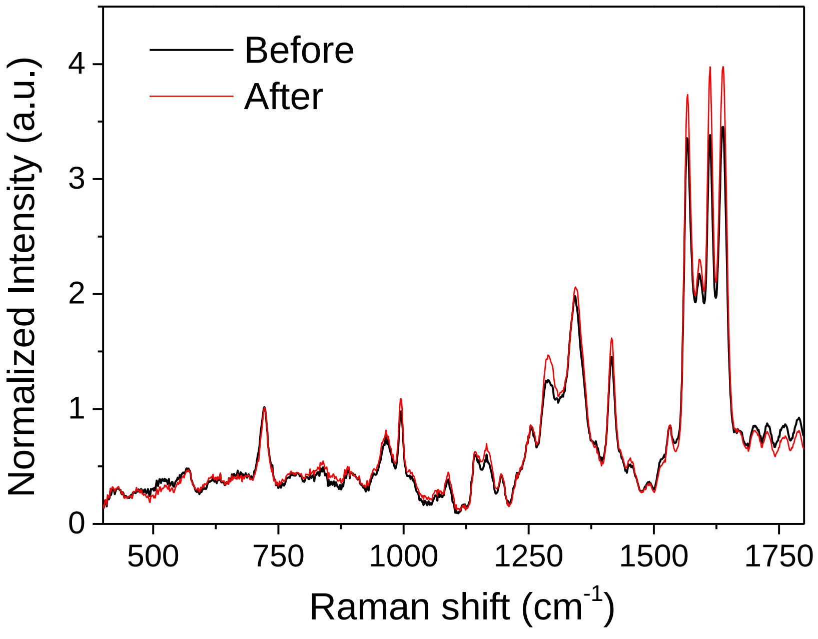


**Figure S104.** Raman analysis of TFB-Phen$_{BM}$ before and after photocatalysis.

In addition to PXRD, the order was analyzed by Raman spectroscopy, as previously reported.[1] Figure S104 shows the Raman spectra of TFB-Phen$_{BM}$ before and after

photocatalysis. Both spectra displayed the main characteristic peaks of Phen[8] (ring deformation: 1612, 1591, 1567, 1417, 1291, and 994 $cm^{-1}$; ring deformation + $CH_3$ scissoring: 1453 $cm^{-1}$; $CH_3$ wagging: 1343 $cm^{-1}$ and $CH_3$ rocking: 965 $cm^{-1}$) together with the characteristic Raman peak at 1637 $cm^{-1}$ for the C=C stretching vibration,[10] which confirmed that the high polycondensation degree and crystallinity were maintained during the photocatalytic experiments. A slight difference in the ratio of the peaks at 1417 (ring deformation) and 1343 $cm^{-1}$ ($CH_3$ wagging) (1.07 before catalysis *versus* 1.15 after catalysis) and the position of the peak at 1417 $cm^{-1}$ (ΔRaman shift = 2 $cm^{-1}$) was observed after catalysis.

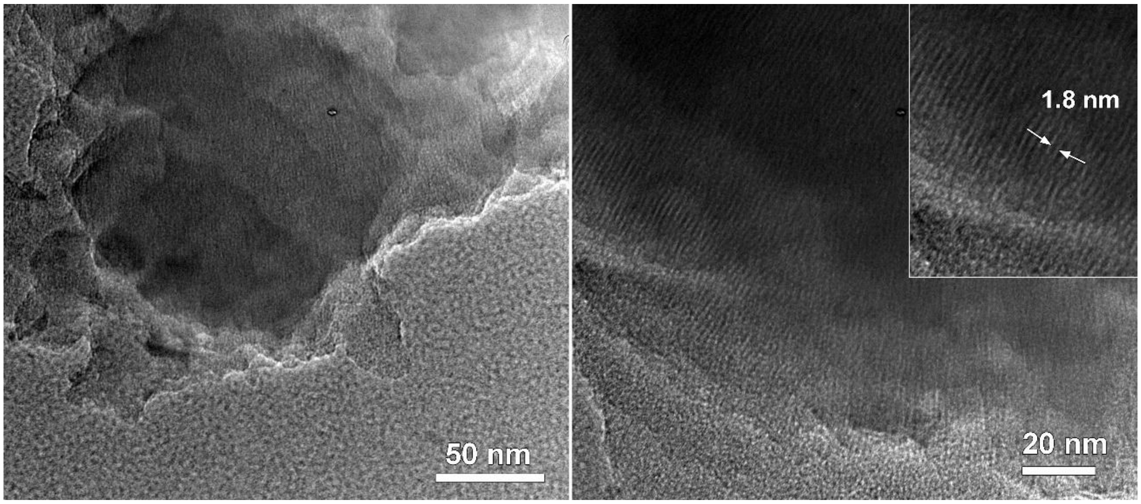


**Figure S105.** Bright field HR-TEM of TFB-Phen$_{BM}$ after photocatalysis.

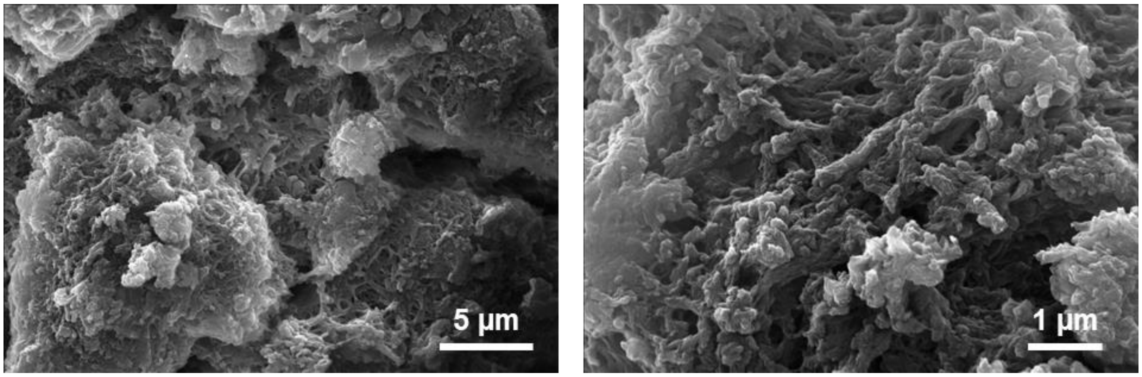


**Figure S106.** SEM micrographs of TFB-Phen$_{BM}$ after photocatalysis.

**Table S7.** Comparison of different materials for MB photodegradation.

| Material | Light source | [Material] mg mL$^{-1}$ | $C_0$ mg L$^{-1}$ | Efficiency | Ref. |
|---|---|---|---|---|---|
| Por-PD-COF | 300 W Xe lamp (λ ≥ 420 nm) | 0.05 | 5 | ~98%, 180 min | [9] |
| Acr-TFB-Zn | λ ≥ 420 nm | 0.2 | 6.4 | ~91%, 150 min | [10] |
| MOF-5 | Natural sunlight | 0.5 | 10 | ~64%, 390 min | [11] |
| MOF-5/GO10 | Natural sunlight | 0.5 | 10 | ~92%, 390 min | [11] |
| UiO-66 (Zr or $NH_2$) | 500 W Xe lamp (λ ≥ 420 nm) | 0.2 | 15 | 58% or 56%, 120 min | [12] |
| ZnTCPc/UiO-66 (Zr or $NH_2$) | 500 W Xe lamp (λ ≥ 420 nm) | 0.2 | 15 | 68% or 90%, 120 min | [12] |
| $Fe_3O_4$@MIL-100(Fe) | 500 W Xe lamp (λ ≥ 420 nm) | 0.1 | 40 | ~25%, 240 min | [13] |
| M-MIL-101(Fe) | 5 W visible light LEDs | 0.5 | 10 | ~75%, 120 min | [14] |
| brookite–rutile bi-crystalline phases $TiO_2$ (BCTi-4) | 8 W, λ ≥ 400 nm | 0.5 | 10 | ~100%, 36 h | [15] |
| NiO/Ag/$TiO_2$ | 400 W halogen lamp (λ ≥ 400 nm) | 0.2 | 5 | ~57%, 60 min | [16] |
| TFB-Phen-BM | 18 W Blue LEDs (λ = 465 nm) | 0.8 | 10 | ~100%, 120 min | This work |

# 10. References

(1) Gonçalves, L. P. L.; Garcia Ben, J.; Strutyński, K.; Rodriguez-Lorenzo, L.; Araújo, J.; Santos, A. S. G. G.; Soares, O. S. G. P.; Pereira, M. F. R.; Kolen'ko, Y. V.; Melle-Franco, M.; Salonen, L. M., Covalent organic frameworks as catalyst support: A case study of thermal, hydrothermal, and mechanical pressure stability of β-ketoenamine-linked TpBD-Me2. *Microporous Mesoporous Mater.* **2024,** *366*, 112916. DOI: 10.1016/j.micromeso.2023.112916.

(2) Zheng, Z.; Zhang, O.; Nguyen, H. L.; Rampal, N.; Alawadhi, A. H.; Rong, Z.; Head-Gordon, T.; Borgs, C.; Chayes, J. T.; Yaghi, O. M., ChatGPT Research Group for Optimizing the Crystallinity of MOFs and COFs. *ACS Cent. Sci.* **2023,** *9* (11), 2161-2170. DOI: 10.1021/acscentsci.3c01087.

(3) Osterrieth, J. W. M.; Rampersad, J.; Madden, D.; Rampal, N.; Skoric, L.; Connolly, B.; Allendorf, M. D.; Stavila, V.; Snider, J. L.; Ameloot, R.; Marreiros, J.; Ania, C.; Azevedo, D.; Vilarrasa-Garcia, E.; Santos, B. F.; Bu, X.-H.; Chang, Z.; Bunzen, H.; Champness, N. R.; Griffin, S. L.; Chen, B.; Lin, R.-B.; Coasne, B.; Cohen, S.; Moreton, J. C.; Colón, Y. J.; Chen, L.; Clowes, R.; Coudert, F.-X.; Cui, Y.; Hou, B.; D'Alessandro, D. M.; Doheny, P. W.; Dincă, M.; Sun, C.; Doonan, C.; Huxley, M. T.; Evans, J. D.; Falcaro, P.; Ricco, R.; Farha, O.; Idrees, K. B.; Islamoglu, T.; Feng, P.; Yang, H.; Forgan, R. S.; Bara, D.; Furukawa, S.; Sanchez, E.; Gascon, J.; Telalović, S.; Ghosh, S. K.; Mukherjee, S.; Hill, M. R.; Sadiq, M. M.; Horcajada, P.; Salcedo-Abraira, P.; Kaneko, K.; Kukobat, R.; Kenvin, J.; Keskin, S.; Kitagawa, S.; Otake, K.-i.; Lively, R. P.; DeWitt, S. J. A.; Llewellyn, P.; Lotsch, B. V.; Emmerling, S. T.; Pütz, A. M.; Martí-Gastaldo, C.; Padial, N. M.; García-Martínez, J.; Linares, N.; Maspoch, D.; Suárez del Pino, J. A.; Moghadam, P.; Oktavian, R.; Morris, R. E.; Wheatley, P. S.; Navarro, J.; Petit, C.; Danaci, D.; Rosseinsky, M. J.; Katsoulidis, A. P.; Schröder, M.; Han, X.; Yang, S.; Serre, C.; Mouchaham, G.; Sholl, D. S.; Thyagarajan, R.; Siderius, D.; Snurr, R. Q.; Goncalves, R. B.; Telfer, S.; Lee, S. J.; Ting, V. P.; Rowlandson, J. L.; Uemura, T.; Iiyuka, T.; van der Veen, M. A.; Rega, D.; Van Speybroeck, V.; Rogge, S. M. J.; Lamaire, A.; Walton, K. S.; Bingel, L. W.; Wuttke, S.; Andreo, J.; Yaghi, O.; Zhang, B.; Yavuz, C. T.; Nguyen, T. S.; Zamora, F.; Montoro, C.; Zhou, H.; Kirchon, A.; Fairen-Jimenez, D., How Reproducible are Surface Areas Calculated from the BET Equation? *Adv. Mater.* **2022,** *34* (27), 2201502. DOI: 10.1002/adma.202201502.

(4) Bi, S.; Meng, F.; Wu, D.; Zhang, F., Synthesis of Vinylene-Linked Covalent Organic Frameworks by Monomer Self-Catalyzed Activation of Knoevenagel Condensation. *J. Am. Chem. Soc.* **2022,** *144* (8), 3653-3659. DOI: 10.1021/jacs.1c12902.

(5) Perkampus, H.-H.; Müller, P.; Knop, J., Dipolmomente der Phenanthroline / Dipole-Moments of Phenanthrolines. *Zeitschrift für Naturforschung B* **1971,** *26* (2), 83-86. DOI: 10.1515/znb-1971-0205.

(6) Meanwell, N. A., The pyridazine heterocycle in molecular recognition and drug discovery. *Med. Chem. Res.* **2023,** *32* (9), 1853-1921. DOI: 10.1007/s00044-023-03035-9.
(7) Liu, Z.-Q.; Zhang, Q.; Liu, Y.-L.; Yu, X.-Q.; Chui, R.-H.; Zhang, L.-L.; Zhao, B.; Ma, L.-Y., Recent contributions of pyridazine as a privileged scaffold of anticancer agents in medicinal chemistry: An updated review. *Biorg. Med. Chem.* **2024,** *111*, 117847. DOI: 10.1016/j.bmc.2024.117847.
(8) Solovyeva, E. V.; Rakhimbekova, A.; Lanchuk, Y. V.; Myund, L. A.; Denisova, A. S., SERS investigation of neocuproine adsorption on silver: Influence of electrode potential on methyl groups. *Journal of Raman Spectroscopy* **2018,** *49* (2), 207-214. DOI: 10.1002/jrs.5265.
(9) Wu, S.; Li, Y.; Wang, T.; Li, H.; Wang, X.; Ma, L.; Zhang, N.; Yue, P.; Li, Y., Design and synthesis of dual functional porphyrin-based COFs as highly selective adsorbent and photocatalyst. *Chem. Eng. J.* **2023,** *470*, 144135. DOI: 10.1016/j.cej.2023.144135.
(10) Chen, Q.; Wang, Y.; Luo, G., Green and Rapid Synthesis of Acridine-Functionalized Covalent Organic Polymers for Photocatalysis by Combining Sonochemistry and Ion Induction. *Langmuir* **2023,** *39* (33), 11731-11740. DOI: 10.1021/acs.langmuir.3c01321.
(11) Bouider, B.; Haffad, S.; Bouakaz, B. S.; Berd, M.; Ouhnia, S.; Habi, A., MOF-5/Graphene Oxide Composite Photocatalyst for Enhanced Photocatalytic Activity of Methylene Blue Degradation Under Solar Light. *J. Inorg. Organomet. Polym. Mater.* **2023**, DOI: 10.1007/s10904-023-02668-y.
(12) Liang, Q.; Zhang, M.; Zhang, Z.; Liu, C.; Xu, S.; Li, Z., Zinc phthalocyanine coupled with UIO-66 (NH2) via a facile condensation process for enhanced visible-light-driven photocatalysis. *J. Alloys Compd.* **2017,** *690*, 123-130. DOI: 10.1016/j.jallcom.2016.08.087.
(13) Zhang, C.-F.; Qiu, L.-G.; Ke, F.; Zhu, Y.-J.; Yuan, Y.-P.; Xu, G.-S.; Jiang, X., A novel magnetic recyclable photocatalyst based on a core–shell metal–organic framework Fe3O4@MIL-100(Fe) for the decolorization of methylene blue dye. *J. Mater. Chem. A* **2013,** *1* (45), 14329-14334. DOI: 10.1039/C3TA13030D.
(14) Fattahi, M.; Niazi, Z.; Esmaeili, F.; Mohammadi, A. A.; Shams, M.; Nguyen Le, B., Boosting the adsorptive and photocatalytic performance of MIL-101(Fe) against methylene blue dye through a thermal post-synthesis modification. *Sci. Rep.* **2023,** *13* (1), 14502. DOI: 10.1038/s41598-023-41451-4.
(15) Kim, M. G.; Lee, J. E.; Kim, K. S.; Kang, J. M.; Lee, J. H.; Kim, K. H.; Cho, M.; Lee, S. G., Photocatalytic degradation of methylene blue under UV and visible light by brookite–rutile bi-crystalline phase of TiO2. *New J. Chem.* **2021,** *45* (7), 3485-3497. DOI: 10.1039/D0NJ05162D.
(16) Mohammed, W.; Matalkeh, M.; Al Soubaihi, R. M.; Elzatahry, A.; Saoud, K. M., Visible Light Photocatalytic Degradation of Methylene Blue Dye and Pharmaceutical

Wastes over Ternary NiO/Ag/TiO2 Heterojunction. *ACS Omega* **2023**, DOI: 10.1021/acsomega.3c01766.
